# Identification and Characterization of the Wilms Tumor Cancer Stem Cell

**DOI:** 10.1002/advs.202206787

**Published:** 2023-04-28

**Authors:** Astgik Petrosyan, Valentina Villani, Paola Aguiari, Matthew E. Thornton, Yizhou Wang, Alex Rajewski, Shengmei Zhou, Paolo Cravedi, Brendan H. Grubbs, Roger E. De Filippo, Sargis Sedrakyan, Kevin V. Lemley, Marie Csete, Stefano Da Sacco, Laura Perin

**Affiliations:** ^1^ GOFARR Laboratory Children's Hospital Los Angeles Division of Urology Saban Research Institute Los Angeles CA 90027 USA; ^2^ Keck School of Medicine University of Southern California Los Angeles CA 90033 USA; ^3^ David Geffen School of Medicine at UCLA – VA Healthcare System Los Angeles CA 90095 USA; ^4^ Department of Obstetrics and Gynecology Keck School of Medicine University of Southern California Los Angeles CA 90033 USA; ^5^ Genomics Core Department of Biomedical Sciences Cedars‐Sinai Medical Center Los Angeles CA 90048 USA; ^6^ Department of Pathology and Laboratory Medicine Children's Hospital Los Angeles Los Angeles CA 90027 USA; ^7^ Department of Medicine Division of Nephrology and Translational Transplant Research Center Recanati Miller Transplant Institute Icahn School of Medicine at Mount Sinai New York NY 10029 USA; ^8^ Children's Hospital Los Angeles Division of Nephrology Department of Pediatrics University of Southern California Los Angeles CA 90027 USA; ^9^ Department of Anesthesiology University of Southern California Los Angeles CA 90033 USA

**Keywords:** cancer, nephron progenitors, Wilms tumor

## Abstract

A nephrogenic progenitor cell (NP) with cancer stem cell characteristics driving Wilms tumor (WT) using spatial transcriptomics, bulk and single cell RNA sequencing, and complementary in vitro and transplantation experiments is identified and characterized. NP from WT samples with NP from the developing human kidney is compared. Cells expressing SIX2 and CITED1 fulfill cancer stem cell criteria by reliably recapitulating WT in transplantation studies. It is shown that self‐renewal versus differentiation in SIX2+CITED1+ cells is regulated by the interplay between integrins ITG*β*1 and ITG*β*4. The spatial transcriptomic analysis defines gene expression maps of SIX2+CITED1+ cells in WT samples and identifies the interactive gene networks involved in WT development. These studies define SIX2+CITED1+ cells as the nephrogenic‐like cancer stem cells of WT and points to the renal developmental transcriptome changes as a possible driver in regulating WT formation and progression.

## Graphical abstract

These studies show that SIX2+CITED1+ cells are cancer‐like stem cells of Wilms tumor (WT) and highlight that the interplay between ITG*β*1 and ITG*β*4 regulates their fate. Spatial transcriptomics, RNA‐seq, and in vitro and transplantation experiments point to the renal developmental transcriptome changes as a possible driver in regulating WT formation and progression.

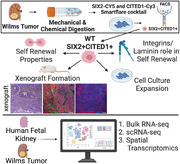

## 1 Introduction

Normal human nephrogenesis involves the reciprocal interaction between two embryonic layers: the branching ureteric bud (UB) and the surrounding cap mesenchyme (CM).^[^ ^1^ ^]^ Induction by the UB initiates condensation of the CM and the beginning of further renal development characterized by the formation of peritubular aggregates, followed by renal vesicles, C‐shaped and S‐shaped bodies, leading to full maturation into a functional nephron.^[^ ^1^ ^]^ Many studies confirm the presence of self‐renewing, uncommitted nephrogenic progenitors (NP) in a specific subdomain of the CM, co‐expressing the transcription factors SIX2 and CITED1, the master genes regulating nephrogenesis.^[^ ^2^, ^3^ ^]^ Importantly, proper nephrogenesis is regulated by these uncommitted progenitors expressing SIX2 and CITED1 and by the committed progenitors that lose CITED1 but still maintain SIX2 expression.^[^ ^1^, ^2^, ^3^ ^]^ Loss of CITED1 primes the cells to renal differentiation; thus, the tight regulation of SIX2 and CITED1 expression is critical during normal kidney development. In humans, kidney development ceases around 34–36 weeks gestational age (WGA)^[^ ^4^ ^]^ and these NP are absent in the mature postnatal kidney.

Growing evidence links Wilms tumor (WT), which accounts for 95% of all pediatric kidney cancers,^[^ ^5^ ^]^ to aberrant nephrogenesis, in which normal prenatal depletion of NP does not occur, and NP persists in the neonatal kidney. Histologically, WT resembles an embryonic kidney, with tumors containing multiple developing renal structures.^[^ ^6^, ^7^ ^]^ All three lineages of the developing kidney (blastema, epithelium, and stroma) can be identified in classic triphasic WT histology.^[^ ^8^ ^]^

The combination of surgery, radiation, and chemotherapy has increased the survival rate for many WT patients.^[^ ^9^ ^]^ However, patients with relapsed disease or initial unfavorable histopathology have a poor prognosis; thus, a better understanding of molecular mechanisms that regulate tumor development and progression is necessary to improve treatments for these WTs.^[^ ^9^ ^]^

Many studies have focused on characterizing the complicated genetic causes of WT, and ≈40 genes that drive diversity in the genetic landscape of WT have been identified.^[^ ^10^ ^]^ Even if many of these WT genes, including WT1 and *β*‐catenin, are known to play important roles in NP of the developing kidney,^[^ ^11^ ^]^ and the presence of cells characterized by the expression of SIX2 and CITED1 has been described in WT samples,^[^ ^12^, ^13^, ^14^, ^15^ ^]^ very few studies have compared NP of WT to NP of the human fetal kidney (hFK) to understand molecular changes responsible for the dysregulation of normal nephrogenesis in WT.

Based on the premise that NP are central to WT development, we isolated and characterized, for the first time, a pure population of live NP co‐expressing SIX2 and CITED1 from WT and normal hFK. We demonstrated that SIX2+CITED1+ cells from WT exhibited nephrogenic cancer stem cell (CSC) traits and, upon transplantation into NOD/SCID mice, formed consecutive xenografts with histological features similar to the WT of origin, as well as metastatic capabilities. By comparing SIX2+CITED1+ NP from different WT samples versus hFK, we showed that SIX2+CITED1+ cells from all these sources displayed NP characteristics, but important expression‐level and phenotypic differences distinguished WT and hFK‐derived cells. Trajectory inference analysis generated from scRNA‐seq data integration of SIX2+CITED1+ cells and cells dissociated from the WT tissue (from which SIX2+CITED1+ cells were isolated) and spatiotemporal mapping suggested that SIX2+CITED1+ cells drive WT development. We further showed that the interaction between these cells and the surrounding extracellular matrix (ECM) through integrin signaling influences NP self‐renewal and renal specification. Using spatial transcriptomic analysis, we defined specific gene map networks of the WT SIX2+CITED1+ cells and correlated these with the histological setting, thus analyzing the role of the microenvironment in defining their molecular and cellular properties. Our studies identified critical differences within the nephrogenic population between WT and hFK and highlighted the disruption of specific signaling pathways driving WT development.

## 2 Results

### 2.1 Isolation and Characterization of SIX2+CITED1+ Cells in WT and hFK

**Figure 1:**
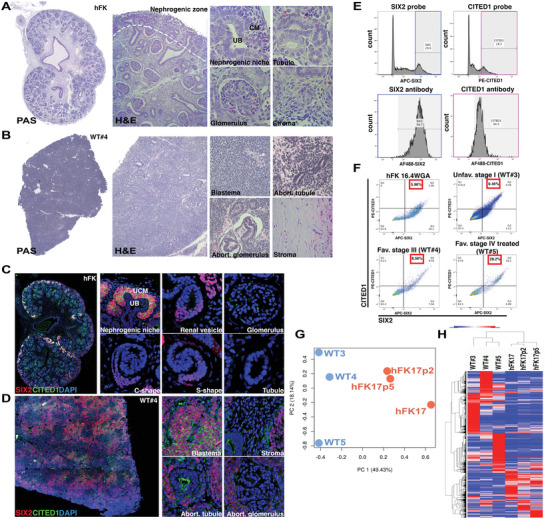
SIX2+CITED1+ cells in hFK and WT have different transcriptional signatures. A,B) Periodic acid Schiff (PAS, left, whole image) and H&E (right, close‐up images) staining of hFK (A,10 WGA) and WT#4 (B, favorable stage III) show the nephrogenic zone (white dotted line) and differentiating structures (second panel: ureteric bud, UB; cap mesenchyme CM; tubule, glomerulus, and stroma) of hFK, and unorganized WT histology with triphasic components (second panel, stroma, blastema, and epithelial structures including abortive glomeruli and tubules). 10× images acquired and composed using Photoshop DC (Adobe) for whole images, right panels of 20X images. C,D) SIX2 (red) and CITED1 (green) immunofluorescence staining of C) hFK 10 WGA and D) WT#4. SIX2+CITED1+ co‐expression in hFK (C, second panel) in the nephrogenic niche (uninduced cap mesenchyme, UCM) but absent within developing (renal vesicle, C‐shape, S‐shape) and mature (glomerulus and tubule) structures. SIX2+CITED1+ expression is dispersed throughout the WT (D, second panels) in blastema but not in stroma or abortive structures (glomerulus and tubule). Nuclei stained with DAPI (blue), 10× images acquired and composed using Photoshop DC (Adobe) for whole images, right panels of 20× images. E) SmartFlare technique validation by flow cytometry. SIX2‐Cy5 and CITED1‐Cy3 probes (top left and right panel respectively) were individually used to isolate cells from hFK (17.4 WGA). Flow cytometry confirmed that 99.7% of SIX2+ cells and 94.3% of CITED1+ cells co‐express both mRNA and protein (bottom left and right panels). F) FACS sorting (by Smartflares): 5.96% of cells from hFK 16.4 WGA are SIX2+CITED1+ cells, 0.46% from WT#3 (unfavorable stage I), 8.56% from WT#4 (favorable stage III) and 28.2% from WT#5 (favorable chemotherapy‐treated stage IV). G,H) Bulk RNA‐seq analysis of hFK (17, 17.2, and 17.5 WGA) and WT (*n* = 3, as in F). PCA (principal component analysis, G) describes 49.43% and 18.14% of the variability, along PC1 and PC2 respectively, within the expression data set. SIX2+CITED1+ cells from WT cluster independently of SIX2+CITED1+ cells from hFK. H) Hierarchical clustering of total gene expression in SIX2+CITED1+ cells from hFK and WT highlights higher similarity among SIX2+CITED1+ cells from different hFK versus higher divergence of SIX2+CITED1+ cells from different WT.

Histologic analysis was used to determine morphological differences and structural organization of developing hFK at different WGA and WT samples (**Table** **1**). Nephrogenesis through gestation can be followed in distinct renal compartments surrounded by organized stroma (**Figure** **1**A, Figure S1A–E, Supporting Information). By 10 WGA, in the hFK, all nephrogenic structures are identifiable, including the nephrogenic niche, mature glomeruli, and tubules. WT, in contrast, appeared histologically disorganized, without recognizable elements of normal renal architecture, and with structural heterogeneity based on the WT subtype (Figure 1B, Figures S1F–J and S2A–I, Supporting Information).

**Table 1:** Clinical descriptions of WT samples analyzed (FACS: fluorescence‐activated cell sorting, WB: Western blot)

#	COG staging	Sex	Anaplastic	Age	Chemo‐treated	Application
1	III	M	Yes	9 year	No	Histology/FACS
2	IV	F	No	2 year	No	Histology
3	I	M	Yes	6 year	No	Bulk RNA‐seq/histology/FACS
4	III	M	No	3 year	No	Bulk RNA‐seq/histology/FACS
5	IV	M	No	14 year	Yes	Bulk RNA‐seq/histology/FACS
6	III	F	No	5 month	No	Histology/FACS
7	I	M	No	1 year	No	Histology
8	II	F	No	7 year	No	In vivo/in vitro/Sc RNA‐seq/histology/FACS
10	III	M	No	3 year	Yes	Histology
11	II	F	No	2 year	No	FACS/WB
12	III	F	No	5 year	No	FACS/WB
13	II	F	No	5 year	No	FACS/WB
14	IV	F	No	3 year	No	Histology
15	II	M	No	2 year	No	Histology/WB
S6	III	M	Yes	9 year	No	Histology
S7	III	F	Yes	4 year	No	Histology
S8	I	F	Yes	6 year	No	Histology
S27	III	M	Yes	1 year	No	Histology

In hFK, SIX2+CITED1+ cells are found exclusively in the nephrogenic zone in CM near the branching UB (Figure 1C). As we have previously reported,^[^ ^2^ ^]^ CITED1 expression is not detected in renal vesicles, C‐shape or S‐shape bodies, where some cells still express SIX2. Mature hFK glomeruli and tubules also do not contain SIX2+CITED1+ cells. The distribution of SIX2+CITED1+ cells in WT depends on WT subtype and differs from hFK. These cells are not restricted to a developmental niche in WT, but rather are dispersed in multiple “blastema foci” and around abortive glomeruli and tubules (Figure 1D; Figure S2A–I, Supporting Information). SIX2+CITED1+ cells were detected in all WT samples included in this study. Using our validated SmartFlare method for live cell sorting^[^ ^2^, ^16^ ^]^ (Figure 1E, Figure S3A–E, Supporting Information), we isolated SIX2+CITED1+ cells from hFK and WT samples. We established that the abundance of SIX2+CITED1+ cells was the same in hFK samples of similar WGA but was highly variable among WT samples (Figure 1F and Figure S3F–H, Supporting Information), likely dependent on the number of blastema foci in each WT.

To study differences in gene expression profile, bulk RNA‐seq data on SIX2+CITED1+ cells from WT samples (WT#3 unfavorable stage I, WT#4 favorable stage III, and WT#5 favorable chemotherapy‐treated stage IV) were compared to hFK SIX2+CITED1+ cells at 17, 17.2, and 17.5 WGA (GEO: GSE176342 and GSE74450^[^ ^2^ ^]^). By principal component analysis (PCA, Figure  1G), hFK and WT samples clustered at opposite sides of the PC1 axis (49.43%). PC2 (18.14%) axis scattering was more pronounced for WT versus hFK SIX2+CITED1+ cells. Hierarchical clustering showed that hFK SIX2+CITED1+ cell gene expression at different WGA is similar, while WT SIX2+CITED1+ cell gene expression patterns were quite dissimilar between samples (Figure 1H).

### 2.2 In Vitro Expansion of hFK and WT SIX2+CITED1+ Cells

To study SIX2+CITED1+ cells, we implemented our existing protocol^[^ ^2^ ^]^ to maintain SIX2 and CITED1 expressing cells in culture. Stem and progenitor cells (including CSC) require a “niche” and specific interaction with the extracellular environment for survival, to maintain stemness, and to receive cues to exit the quiescent state when appropriate.^[^ ^17^ ^]^ During development, the extracellular matrix around NP is specifically organized to support renal differentiation.^[^ ^18^, ^19^ ^]^ Laminins play a key role in directing organogenesis^[^ ^20^ ^]^ including nephrogenesis.^[^ ^19^, ^21^ ^]^ Since LAM511 (laminin *α*5*β*1*γ*1) is critical for nephrogenesis^[^ ^22^, ^23^ ^]^ we focused on this specific laminin.

**Figure 2:**
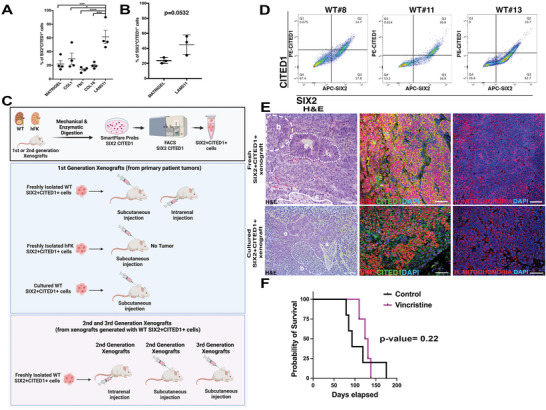
WT‐derived SIX2+CITED1+ cells can be expanded in vitro and generate in vivo xenografts. A flow cytometry analysis of SIX2+CITED1+ cells (%) from hFK (17 WGA) cultured for 5 days on matrigel, collagen I (COL1), fibronectin (FN1), collagen16 (COL16), or laminin511 (LAM511). *p < 0.05; ***p < 0.001; mean ± SEM. B) Flow cytometry analysis of SIX2+CITED1+ cells (%) from hFK (17 WGA) cultured for 28 d on matrigel or LAM511. C) Schematic representation of in vivo limiting dilution and serial xenografts experiments (created with Biorender). D) FACS sorting of SIX2+CITED1+ cells using Smartflare probes from different WT subtypes with favorable histology, stage II (WT#8: 14.7%, WT#11: 30%, and WT#13: 10.7%). E) Representative H&E staining (left panel), SIX2 (red) and CITED1 (green, center) and human mitochondria (red) immunofluorescence staining (right panel) of xenograft generated from freshly isolated WT#8 (favorable stage II)‐SIX2+CITED1+ cells (4 months after injection) and cultured WT#8‐SIX2+CITED1+ cells (passage 6, 4 months after injection). Classical triphasic WT structures are shown (b: blastema, e: epithelium, and s: stroma; white lines). Nuclei stained with DAPI (blue), scale bar = 50 µm. F) Survival analysis of mice after injection of cultured SIX2+CITED1+ cells from WT#8 (favorable stage II) either without treatment (control, *n* = 12) or after vincristine treatment (450 µg kg^−1^ vincristine sulfate IP every 4 days, *n* = 4).

We, therefore, tested if LAM511 can influence self‐renewal in SIX2+CITED1+ cells from both hFK and WT in vitro. First, we seeded SIX2+CITED1+ cells from hFK on different ECM substrates: matrigel, collagen1, fibronectin, collagen16, or LAM511 (Figure 2A; Figure S4A, Supporting Information). A higher percentage of SIX2 and CITED1 co‐expressing cells were present in LAM511 coating after 28 days, while matrigel (often used in nephrogenic culture systems^[^ ^24^ ^]^) had cells with mostly SIX2 expression with loss of CITED1 (Figure 2B; Figure S4B, Supporting Information). Cells cultured without substrate did not attach reliably or proliferate (Figure S4A, Supporting Information). Unlike cells cultured on matrigel, which expressed only cytokeratin (an epithelial cell marker^[^ ^25^ ^]^), cells cultured on LAM511 also maintained vimentin expression, a marker of mesenchymal progenitors^[^ ^26^ ^]^ (Figure S4C, Supporting Information). We also confirmed that this protocol is highly efficient in maintaining self‐renewal capacity and expression of SIX2 and CITED1 in WT SIX2+CITED1+ cell culture(Figure S4D,E, Supporting Information).

### 2.3 SIX2+CITED1+ Cells from WT Meet the criteria for Cancer Stem Cells (CSC)

CSCs are capable of self‐renewal, differentiation, and tumorigenicity in animal hosts and are defined by a specific set of criteria: small numbers of transplanted cells should generate a tumor in vivo, cells should be resistant to chemotherapeutics, and can be identified by specific markers reflecting the tumor of origin.^[^ ^27^, ^28^, ^29^, ^30^ ^]^ WT SIX2+CITED1+ cells tumor formation capabilities were assessed through a series of in vivo experiments (Figure 2C and Figure S5, Supporting Information). Freshly isolated SIX2+CITED1+ cells from different WT samples (Figure 2D) that were injected subcutaneously and intrarenally into NOD/SCID mice generated tumors (Figure 2E, upper panel, and Figure S6, Supporting Information). Limiting dilution experiments also demonstrated the capability of xenograft formation even at low concentrations if injected intrarenally (Figure S6A, Supporting Information). Importantly, culture‐expanded WT SIX2+CITED1+ cells (6–12 passages) injected subcutaneously also generated tumors in vivo (Figure 2E, lower panel). Serial transplantation experiments (2^nd^ and 3^rd^ xenograft generation) also demonstrated the capability of these cells to generate in vivo WT (Figure 2C, Figure S6B, Supporting Information). Transplanted WT SIX2‐CITED1—cells and WT SIX2+CITED1—did not generate tumors within 30 weeks (not shown). These data indicate that the cells responsible for tumor formation in our experimental conditions are the SIX2+CITED1+ cells.

Tumors generated by subcutaneous and intrarenal injection of freshly isolated or cultured WT SIX2+CITED1+ cells had classic WT morphology, including blastema, stroma, and epithelial structures containing SIX2+CITED1‐ cells and SIX2+CITED1+ cells (Figure 2E, Figure S6A,B, Supporting Information). Injected WT#8 SIX2+CITED1+ cells, which had deletions in 7p and 11q (common in relapsed patients), also generated a WT that metastasized to the liver detected 6 months after injection (Figure S6C, Supporting Information).

SIX2+CITED1+ cells from hFK did not form tumors, but in one case, the cells proliferated and generated a “mass” (<0.2 cm at 5 months) without histologic features of WT and no expression of SIX2 and/or CITED1 (Figure S6D, Supporting Information). Histologically, the “mass” appeared to contain primitive normal tubular structures. Some post‐transplantation proliferation of hFK NPs is expected since the cells have intrinsic proliferative potential^[^ ^31^, ^32^ ^]^ before committing to differentiation.

To evaluate resistance to chemotherapeutic drugs, we treated NOD/SCID mice injected with WT SIX2+CITED1+ cells with vincristine, commonly used clinically for WT.^[^ ^33^, ^34^, ^35^, ^36^ ^]^ Standard vincristine dosing protocols for mice^[^ ^36^ ^]^ did not delay or prevent tumor growth (Figure 2F), suggesting that WT SIX2+CITED1+ cells also manifest chemotherapy resistance. We recognize that different dosing regimens^[^ ^37^ ^]^ of vincristine might result in variations in response rates. Here we showed proof of the principle that xenografts generated from SIX1+CITED1+ cells (and not from WT tissue, as reported in previous publications ^[^ ^33^, ^34^, ^35^, ^36^, ^37^ ^]^) show resistance to this specific vincristine regimen.

### 2.4 The Role of ITG*β*1 and ITG*β*4 in Self‐Renewal and Renal Specification in SIX2+CITED1+ Cells

LAM511 is important in renal development, but together with LAM332 and LAM111, it can promote carcinogenesis.^[^ ^38^ ^]^ LAM511‐cell interaction is largely mediated by integrins ITG*α*3*β*1, ITG*α*6*β*1, and ITG*α*6*β*4.^[^ ^39^ ^]^ Though the role of ITGs in cancer progression is complex and still not fully elucidated,^[^ ^40^, ^41^ ^]^ induction of drug resistance in renal carcinoma cells is associated with changes in ITG*β*1 expression.^[^ ^42^, ^43^ ^]^ ITG*β*4 has been identified in aggressive breast CSC^[^ ^44^ ^]^ and ITG*β*4‐targeted immunotherapies showed some efficacy against both CSCs and non‐CSCs in breast and colon carcinoma mouse models.^[^ ^41^, ^45^ ^]^

**Figure 3:**
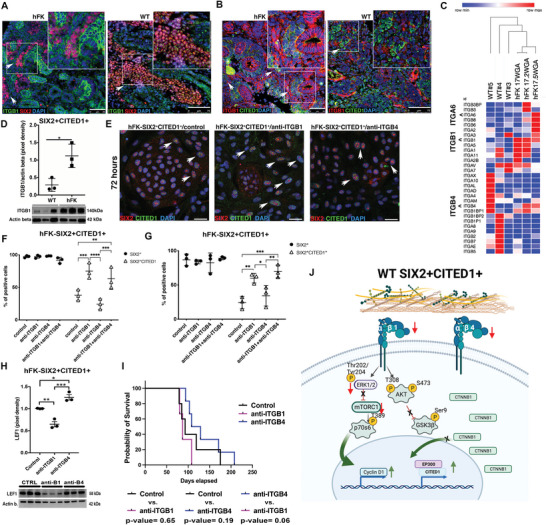
SIX2+CITED1+ cell self‐renewal: the role of the extracellular matrix niche. A,B) Representative immunofluorescence staining showing the distribution of ITG*β*1 (green) and SIX2 (red) in hFK (10 WGA) and WT (WT#12, favorable stage III) A) and for ITG*β*1 (red) and CITED1 (green) in hFK (10 WGA) and WT (WT#8 favorable stage II. B) Nuclei stained with DAPI (blue); scale bars 50 and 75 µm, respectively. C) Heatmap showing gene expression profile for integrins in SIX2+CITED1+ cells from hFK (17, 17.2, and 17.5 WGA) and WT (WT#3 anaplastic stage I, WT#4: non‐anaplastic, stage III, and WT#5: non‐anaplastic chemo‐treated, stage IV). D) Densitometric analysis by western blot (WB) of ITG*β*1 expression in freshly isolated SIX2+CITED1+ cells from WT (WT#8,11,12, favorable stage II, favorable stage III, and favorable stage II) versus SIX2+CITED1+ cells from hFK (15,16,18 WGA) showing higher expression of ITG*β*1 in hFK cells; *β*‐actin was used as housekeeping protein for normalization. WB bands are presented below the graph, *p < 0.05. E) Representative immunofluorescence staining of SIX2 (red) and CITED1 (green) in SIX2+CITED1+ cells from hFK (17 WGA) cultured for 72 h with/without 1 µg mL^−1^ anti‐ITG*β*1 or 0.5 µg mL^−1^ anti‐ITG*β*4 neutralizing antibody showing increased expression of CITED1 in cells treated with anti‐ITG*β*1. Nuclei stained with DAPI (blue). Scale bar = 50 µm. F,G) Percentage of SIX2+CITED1+ cells and total SIX2+ cells from hFK (17 WGA) by flow cytometry analysis after F) 5 d or G) 28 d of culture with/without anti‐ITG*β*1 or anti‐ITG*β*4 neutralizing antibody or a combination of both. *p < 0.05, **p < 0.01, ***p < 0.001, ****p < 0.0001; mean ± SEM. H) Densitometric analysis by WB of LEF1 protein expression in hFK SIX2+CITED1+ cells (17 WGA) cultured for 28 d with/without anti‐ITG*β*1 or anti‐ITG*β*4 neutralizing antibody *β*‐actin was used as housekeeping control. WB bands are presented below the graph. *p < 0.05. I) Kaplan‐Meier survival analysis of mice injected with WT SIX2^+^CITED1^+^ cells, without treatment (control, *n* = 4) or with the treatment of anti‐ITG*β*1 (*n* = 5) or anti‐ITG*β*4 (*n* = 4), endpoint tumor size 1.5 cm. J) Schematic representation showing the proposed role of ITG*β*1 and ITG*β*4 in WT SIX2+CITED1+ cells.

To examine the role of LAM511‐ITG binding in WT SIX2+CITED1+ cells, we first determined the localization of ITG*β*1 and ITG*β*4 in hFK and WT. ITG*β*1 and ITG*β*4 were expressed in various developing structures in the hFK, including UB and CM, in proximity to SIX2+ and CITED1+ cells, while expression of ITG*β*1 and ITG*β*4 in WT was more diffuse (**Figure** **3**A,B; Figure S7A, Supporting Information). Transcriptomic and protein analysis revealed different patterns of expression of these two ITG (Figure 3C,D), with *ITGβ1* being more highly expressed in hFK SIX2+CITED1+ cells compared to WT SIX2+CITED1+ cells.

We next tested if LAM511‐ITG*β*1 and LAM511‐ITG*β*4 signaling regulate the NP state in cultured hFK SIX2+CITED1+ cells. Antibody neutralization of ITG*β*1 increased the percentage of cells co‐expressing SIX2 and CITED1 but did not change the percentage of cells expressing SIX2 (at 72 h, 5 d, 28 d, Figure 3E–G). Similar results were achieved using treatment with an antibody specific to the active form of ITG*β*1 (9EG7),^[^ ^46^, ^47^ ^]^ while no changes in SIX2+CITED1+ cells were detected by activating ITG*β*1 with MnCl2, which induces localized conformational changes mimicking ligand‐receptor occupancy^[^ ^48^ ^]^ (Figure S7B, Supporting Information).

Neutralization of ITG*β*4 did not promote an increase in the percentage of SIX2+CITED1+ cells but did stimulate expression of LEF1, one of the first genes expressed in committed NP that determine cell cycle exit to differentiation.^[^ ^2^, ^3^, ^49^, ^50^ ^]^ On the contrary, LEF1 expression was significantly reduced by anti‐ITG*β*1 treatment (Figure 3H). Blockage of ITG*β*4 also activated components of WNT signaling (*CSNK2A1, WNT4, CSNK2B, WNT10A, CSNK1D*, and *CSNK1G2*), which are required for commitment (Figure S7C, Supporting Information).^[^ ^51^ ^]^ Finally, in mouse recipients of WT SIX2+CITED1+ cells, the speed of tumor growth tended to be enhanced by anti‐ITG*β*1 (Figure 3I) versus mice treated with anti‐ITG*β*4.

We next explored two major downstream pathways of integrin signaling: MAPK/ERK and PI3K‐Akt‐mTOR,^[^ ^52^, ^53^, ^54^, ^55^, ^56^ ^]^ both important during nephrogenesis^[^ ^57^ ^]^ (Figure S8, Supporting Information). ERK phosphorylation is efficiently prevented by ITG*β*1 blockade, whereas ITG*β*4 blockade is ineffective (Figure S8A, Supporting Information). In contrast, inhibition of ITG*β*1 signaling increases the number of SIX2+CITED1+ cells and causes limited activation of AKT (limited to Thr308) and increased cyclin D1 (Figure S8B–D, Supporting Information), which shortens G1, promoting self‐renewal. Interestingly, while inhibition of ITG*β*4 did not significantly affect ERK or AKT, it did increase expression of LEF1 (as shown above Figure 3H) and EP300 (Figure S8H, Supporting Information); both are typically upregulated during NP induction and commitment. Concomitant neutralization of ITG*β*1 and ITG*β*4 prevents differentiation (Figure 3F,G), suggesting that ITG*β*1 signaling prevails over ITG*β*4 since overexpression of CITED1 prevents EP300 binding to *β*‐catenin, blocking NP induction (Figure 3J, Figure S8 and Discussion S1, Supporting Information).

### 2.5 Transcriptomic Analysis Reveals a Paucity of Nephrogenic Differentiation Genes Expressed in WT SIX2+CITED1+ Cells

**Figure 4:**
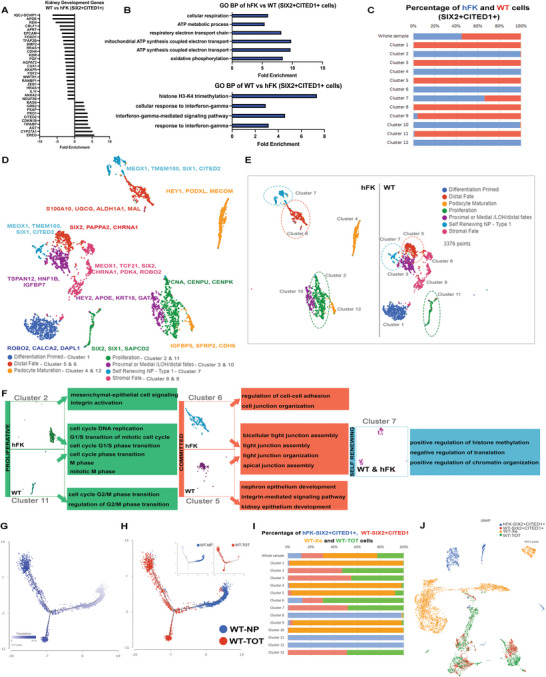
Bulk and scRNA‐seq analysis reveal heterogenicity of hFK and WT SIX2+CITED1+ cells. A) Ingenuity Pathway Analysis (IPA) of nephrogenic development‐specific genes in SIX2+CITED1+ cells derived from WT versus hFK. The graph shows significantly upregulated and downregulated genes in the nephrogenic development pathway in WT. B) Gene Ontology (GO) sets enriched in hFK SIX2+CITED1+ cells (top) and WT SIX2+CITED1+ cells (bottom) are visualized by fold enrichment score. *P* < 0.05. Upregulated DE genes were used for each comparison. C) Fraction of cells (% of cells; *x*‐axis; hFK, blue; WT, red) in each cluster (*y*‐axis). D) Uniform manifold approximation and projection (UMAP) of 3376 droplet‐based scRNA‐seq profiles of SIX2+CITED1+ cells from hFK (16 WGA) and WT#8 (favorable stage II), generated by unsupervised assignment of clusters. Clusters are labeled (bottom) by post‐hoc annotation based on relevant differentially expressed nephrogenic genes. E) Split‐by‐sample UMAP, highlighting how the different cell subpopulations are divided between WT and hFK samples. F) GO analysis for selected clusters shows shared enrichment for proliferative (clusters 2 and 11) and differentiating (clusters 5 and 6) gene sets. *P* < 0.05. Upregulated DE genes were used for each comparison. Common cluster 7 is highly enriched for genes involved in methylation and chromatin organization. G) Trajectory and H) pseudo‐time ordering of the integration of WT SIX2+CITED1+ cells and WT‐TOT (tumor of origin from which the SIX2+CITED1+ cells were obtained) arranged into a major trajectory bifurcating into two branches representing divergent differentiation paths. (H, top panel: blue: WT‐NP; red: WT‐TOT). I) Distribution of SIX2+CITED1+ cells from hFK (blue), SIX2+CITED1+ cells from WT (red), WT‐Xe (xenograft from freshly isolated and transplanted WT#8 SIX2+CITED1+ cells, yellow) and WT‐TOT (total primary WT) samples (green) to each cluster. J) UMAP of 10121 droplet‐based scRNA‐seq profiles from the integration of SIX2+CITED1+ cells from hFK (blue), SIX2+CITED1+ cells from WT (red), WT‐Xe (yellow) and WT‐TOT (green) samples.

Bulk transcriptomics of WT SIX2+CITED1+ samples versus hFK SIX2+CITED1+ samples were then analyzed using Ingenuity pathway analysis (IPA) for genes involved in renal development pathways.^[^ ^1^, ^2^, ^58^ ^]^ We identified that genes involved in renal specification and differentiation (*APOE, REN, FOXD1, BMP2, FGF2*) were under‐expressed in WTs, whereas genes associated with the uncommitted state, pluripotency, and proliferation (*CITED2, CDKN1B*, *EREG*) were highly expressed (**Figure** **4**A). Gene Ontology (GO) analysis of biological processes showed increased expression of cellular respiratory and ATP metabolic processes in hFK versus WTs and histone H3‐K4 trimethylation and INF‐gamma signaling pathway in WT versus hFK (Figure 4B). Individual samples were also analyzed (IPA and GO analysis, Figure S9 and Dataset S#1‐2, Supporting Information).

To study the heterogeneity of SIX2+CITED1+ cells, scRNA‐seq analysis (GSE175698) was performed on SIX2+CITED1+ cells from WT#8 (favorable stage II) and hFK (17 WGA, Figure S10, Supporting Information) after removing CD45+ immune cells from the analysis (Figure S11, Supporting Information).

By integration analysis, we identified 12 distinct cell clusters (Figure 4C,D). Except for cluster 7 (almost equally shared between the two samples), the clusters were exclusively or preponderantly represented by either hFK or WT cells (Figure 4C–E). WT SIX2+CITED1+ cell clusters were highly enriched in *WT1* (Figure S12A,B, Supporting Information) but devoid of *H19* (tumor suppressor gene) expression (Figure S12C–E, Supporting Information), confirming the previous findings.^[^ ^6^, ^59^, ^60^ ^]^ Since *SIX2* and *CITED1* expression (Figure S12F,G, Supporting Information) was very low and deeper sequencing was prevented by saturation of the samples, qPCR was performed to confirm *CITED1* and *SIX2* expression (Figure S12H, Supporting Information).

Using the cell characterization of the hFK by Lindström et al.,^[^ ^61^ ^]^ we stratified clusters based on the expression of specific nephrogenic genes (Figure 4D,E). We identified 7 different cell types, and interestingly, the clusters identifying early podocyte maturation (clusters 4 and 12) were present only in hFK, suggesting that WT SIX2+CITED1+ cells might not be able to initiate podocyte commitment correctly. Only two clusters (8 and 9) appeared committed, but they lacked a specific differentiation signature defined in;^[^ ^61^ ^]^ these clusters were enriched in angiogenesis and mesenchymal stem cell maintenance pathways, possibly reflecting a stromal phenotype. These results were also confirmed by GO enrichment, IPA analysis, and hierarchal clustering (Figure 4F, Figure S12I–L and Dataset S#3‐4, Supporting Information) and are further discussed in Discussion S2 (Supporting Information). We also detected by scRNA‐seq differences in *ITGβ1* and *ITGβ4* expression between hFK and WT SIX2+CITED1+ cells, thus further supporting their role in regulating these cells (Figure S12M–O, Supporting Information).

Cell cycle regulation is critical to balancing proliferation versus differentiation, FACS, and pathways involved in cell cycle regulation stratified distinctly between hFK and WT. Cluster 2 (hFK) expressed fewer G1/S checkpoint genes, while cluster 11 (WT) expressed fewer G2/M checkpoint control genes. To further understand the role of the cell cycle in SIX2+CITED1+ cells, we investigated the expression of phase‐specific genes^[^ ^62^ ^]^ within clusters (Figure 4F; Figure S13A–F, Supporting Information). Though most clusters expressed genes for G1/S (Figure S13A, Supporting Information), G2 (Figure S13B, Supporting Information), and M/G1 (Figure S13D, Supporting Information), WT proliferative cluster 11 almost exclusively expressed markers for G2/M progression (Figure S13C, Supporting Information) as well as symmetric cell division (Figure S13E, Supporting Information). Upregulation of G2/M progression pathways, confirmed by flow analysis showing increased numbers of cells in G2/M in WT samples (Figure S13F, Supporting Information), likely reflecting rapid proliferation in WT cells.

To decipher the renal commitment signature of the SIX2+CITED1+ cells, we perform trajectory analysis on the SIX2+CITED1+ cells together with the tumor from which they were originally selected (WT total, WT‐TOT) and the xenograft that they generated in vivo, identifying 3 distinct states. The distribution of the integrated samples along the trajectory is shown in Figure 4G. To assess the temporal progression to differentiation, we performed pseudotime analysis and determined the pseudotemporal ordering of the cells along the trajectory (Figure 4H), which produced a major trajectory (right side) bifurcating into two major branches identified as upper and lower. Interestingly, when samples are visualized along the pseudotime, WT SIX2+CITED1+ cells segregated toward the earlier stages of the developmental trajectory (right branch) with some cells scattered along the left lower bifurcation (Figure  4G, top panel; WT‐NP: blue, WT‐TOT: red). On the other hand, WT‐TOT cells were more scattered along the pseudotime scale, with a small cluster of cells localized together with WT SIX2+CITED1+ along the right‐side branch and a larger portion of the cells populating the two developmentally more mature branches (left side, upper and lower).

To further study the tumorigenicity of SIX2+CITED1+ cells from WT, we compared their gene expression pattern with that of WT‐TOT. scRNA‐seq data (Figure S14A–D, Supporting Information) revealed a strong overlap between the tumor of origin and the SIX2+CITED1+ cells (Figure S14A,B, Supporting Information). By PCA, WT SIX2+CITED1+ and WT‐TOT samples varied minimally (Figure S14C, Supporting Information). An exception was cluster 4, predominantly composed of WT‐TOT cells (Figure S14D, Supporting Information) and characterized by enrichment for genes involved in the cellular response to metal ions (Figure S14E, Supporting Information), including *MT1A* metallothionine and *SOD1* (Figure S14F,G and Dataset S#5, Supporting Information). While metallothionines have known roles in carcinogenesis, including in some renal carcinomas,^[^ ^63^ ^]^ this is the first association of these genes with WT and may suggest an acquired resistance of the tumor to metal toxicity. We also saw a consistent expression of superoxide dismutase 1 (*SOD1*, involved in detoxification of reactive oxygen^[^ ^64^, ^65^ ^]^) across WT clusters (Figure S14G and Dataset S#5, Supporting Information). Additionally, scRNA‐seq analysis showed enrichment of drug resistance genes such as *ABC* genes in WT versus hFK clusters (Figure S14H, Supporting Information), possibly explaining the drug resistance of the xenografts originated from these cells in our in vivo experiments (Figure 2F).

Integration of scRNA‐seq data (Figure 4I,J, GSE175698) showed distinct differences in gene expression between SIX2+CITED1+ cells from hFK and WT, WT xenografts and the tumor of origin (WT‐TOT); similarities between WT SIX2+CITED1+ cells and their tumor of origin (WT‐TOT) are evident. The transcriptomic analysis confirmed the high similarity of the xenografts generated from freshly isolated versus expanded SIX2+CITED1+ cells (Figure S15A,B, Supporting Information). Tumors generated from freshly isolated or cultured SIX2+CITED1+ cells exhibited minimal transcriptional differences from the primary WT, as visualized by PCA (Figure S15C,D, Supporting Information).

### 2.6 Spatial Transcriptomic (ST) Analysis Reveals Unique Transcriptomic Maps of WT versus hFK

To correlate histological organization with gene expression, we characterized WT samples using spatial maps of gene expression patterns with the Visium 10X Genomics Platform, using normal hFK as a reference (Figure S16, Supporting Information and Methods for details, GSE178349, GSM5388190, GSM5388191, and GSM5388192). A detailed spatial characterization of the samples individually is reported in Discussion S2, Figure S17, and Dataset S#6 (Supporting Information). Briefly, this analysis showed that, unlike the hFK in which genes reflecting all stages of nephrogenesis can be recognized in distinct clusters that histologically identified the nephrogenic niches and more differentiated renal structures, in WT these nephrogenic genes are spatially scattered without relation to clearly recognizable structures and mixed with areas characterized by gene expression of aberrant (particularly muscle) differentiation.

**Figure 5:**
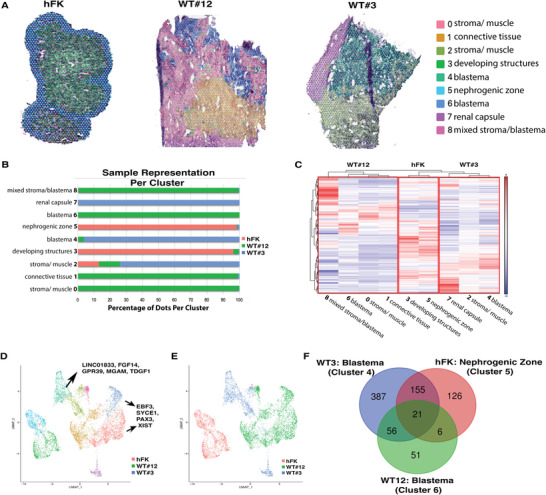
Spatial transcriptomics (ST) analysis of integrated data from hFK, WT#12 and WT#3. A) ST performed on integrated data of hFK (16.6 GWA), WT#12 favorable stage III, and WT#3 unfavorable stage I identified 9 clusters by unsupervised clustering. Histological identification of morphological regions within the integration analysis is shown. B) Fraction of spots (*x*‐axis) from each sample in each cluster (*y*‐axis). C) Hierarchical clustering of all identified genes within the 9 clusters stratified based on integrated samples. D) Uniform manifold approximation and projection (UMAP) of 7282 spot‐based ST from the integrated samples, colored by clusters generated by unsupervised assignment. Specific cluster genes are reported. E) UMAP of 7282 spot‐based ST from the integration of hFK (red), WT#3 (blue), and WT#12 (green). F) Venn diagram of differentially expressed genes (DEG) in hFK nephrogenic zone cluster 5 (red) and WT#3 (blue) and 12 (green) blastema clusters 4 and 6, respectively. Only DEG with average log fold change >0.5 or ←0.5 and adjusted p‐value <0.05 were included. The list and distribution of genes included in the VENN diagram can be found in Dataset S#8 (Supporting Information).

ST was then used to study differences between clusters in WT versus hFK (**Figure** **5**A–E) by integration analysis; 9 clusters were identified. Hierarchical clustering showed that clusters representative of each sample grouped together, with WT#3 clustering closer to hFK (Figure 5C) than WT#12.

Clusters 0, 1, 6, and 8 (mostly representing WT#12, histologically defined as favorable, Figure 5B) corresponded to histological compartments of stroma with marked rhabdomyomatous differentiation, blastema, connective tissue, and mixed stroma/blastema, respectively. By GO analysis (Figure S18 and Dataset S#7, Supporting Information), these clusters were highly enriched in non‐renal development pathways (specifically muscle and bone differentiation, heart, and vasculature development), metabolism, and cell cycle regulation but with a clear paucity of kidney development genes (glomerulus and tubule morphogenesis, UB and metanephric mesenchyme development). These data showed a lack of nephrogenesis specification genes in WT expressing aberrant non‐renal differentiation pathways, a feature shared by many tumors other than WT.^[^ ^66^, ^67^ ^]^

Cluster 4 (spatially a blastema, specific of WT#3, histologically defined as unfavorable) was highly enriched in kidney development genes (including those associated with metanephric mesenchyme, and UB branching), with downregulation of other tissue/organ differentiation, cell cycle, and metabolic pathways. Cluster 7 (WT capsule with fibrous tissue, also specific to unfavorable WT) highly expressed immunological response genes (adaptive, innate, and humoral) with downregulation of metabolic, IL12, and cell cycle pathways.

Cluster 2 (expressed by all samples with many spots from WT#3, with histology mixed between blastema and stroma) was highly represented by GO sets specific for mesonephric and ureteric development, possibly representing a “common” nephrogenic cluster shared by hFK and both types of WT (Figure S18, Supporting Information).

Clusters 3 and 5 (largely specific to hFK, with cluster 5 representing the CM and cluster 3 more differentiated structures) highly expressed GO sets typical of all stages of nephrogenesis (Figure S18, Supporting Information). As in Figure S19 (Supporting Information), though clusters 3 and 5 expressed multiple GO sets related to kidney development, specific expression patterns and tissue localization suggested that cluster 3 is more induced toward nephrogenic differentiation (expression of *NPHS1* and *PTPTO*), while cluster 5 is in a more uncommitted state (expression of *PAX2* and *SALL1*).

Next, we investigated the differences between the blastema of the WT patient samples (clusters 4 and 6) against the nephrogenic zone of the hFK (cluster 5). Even if these clusters shared some similarities (Figure 5F), distinct gene expression patterns were noted in WT blastema versus the hFK nephrogenic zone. GO analysis, identified by DE genes between the groups (upregulated in Figure S20A,B, Supporting Information; downregulated in Figure S20C,D, Supporting Information) showed enrichments of renal development pathways with reduced cell death regulation, response to heat and peptide metabolic processes in the hFK. WT#3 blastema showed enrichment of immune related pathways and the reduction of regulation of cell motility, extracellular matrix organization, and blood vessel formation, while WT#12 blastema showed enrichment of cell cycle, proliferation, and hypermethylation pathways and reduction in kidney development pathways. This data highlighted that although histologically similar, the blastema portions of the tumors differ from the hFK nephrogenic zone by a distinct set of genes and molecular pathways (Dataset S#8, Supporting Information).

We then compared the histologically represented blastema in both WT patient samples (clusters 6 and 4, Figure S19, Supporting Information). DE gene analysis between these 2 clusters revealed important differences in gene expression between WT samples, allowing us to identify specific blastema transcriptomic maps. We identified potential targets of interest specific to different WT blastema (Figure S21, Supporting Information), such as *CLEC4M* (Figure S21B, Supporting Information); these data were also confirmed by immunohistochemistry in multiple patient samples, as shown in Figure S21E–L, and Dataset S#7 (Supporting Information) and discussed in Discussion S2 (Supporting Information).

### 2.7 Spatial Analysis of the SIX2+CITED1+ Cells Reveals Intrinsic Transcriptomics Differences in WT versus hFK

**Figure 6:**
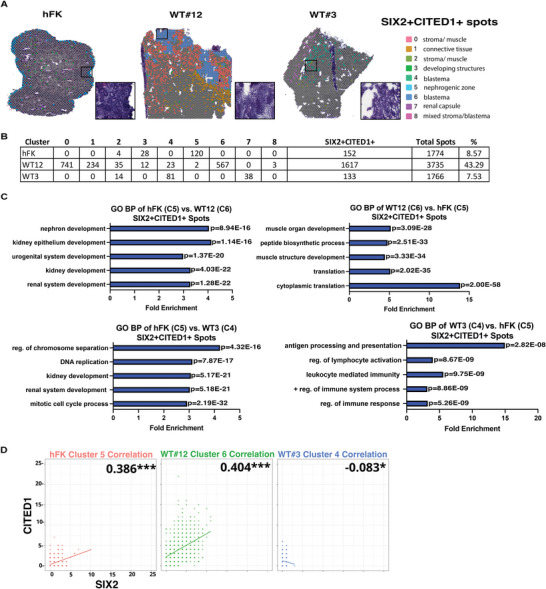
Spatial transcriptomics (ST) analysis of SIX2+CITED1+ spots from hFK and WT reveals molecular heterogeneity of histologically similar structures in different WT. A. ST visualization of SIX2+CITED1+ spots in hFK, WT#12, and WT#3 obtained by unsupervised clustering of ST performed on integrated data. The hFK SIX2+CITED1+ spots are localized in the nephrogenic zone (identified by cap mesenchyme as shown by the H&E, black box). Zoomed in H&E representing the cap mesenchyme in hFK and the blastema component in WT#12 and WT#3 that are spread throughout the WT.  B) Table summarizing the distribution of SIX2+CITED1+ spots per cluster and the percentage of total SIX2+CITED1+ spots compared to the total number of spots per sample (right side column) C) List of top gene ontology (GO) biological process of hFK nephrogenic zone (cluster 5, SIX2+CITED1+ spots) compared to WT#3 blastema (cluster 4) and WT#12 blastema (cluster 6) SIX2+CITED1+ spots (left panels) and in WT#3 blastema (cluster 4) or WT#12 blastema (cluster 6) SIX2+CITED1+ spots compared to hFK nephrogenic zone (cluster 5, SIX2+CITED1+ spots, right panels). Fold enrichment of each gene set is shown, *P* < 0.05; upregulated DE genes were used for each comparison. D) Pearson correlation between the expression of *CITED1* and *SIX2* was performed on SIX2+CITED1+ spots found in clusters of WT#3 (cluster 4), hFK (cluster 5), and WT#12 (cluster 6), with dots representing spots; dots color intensity indicating the number of spots superimposed with a similar expression of *SIX2* (x‐axis) and *CITED1* (y‐axis).

We next used ST data to identify the spatial localization of these cells in the WT samples versus hFK. **Figure** **6**A,B shows that spots expressing SIX2 and CITED1 were present mainly in cluster 5 in the hFK (as expected, since this cluster represents the nephrogenic zone), while in WT, expression of these two genes was present in multiple clusters that histologically identified the blastema (4 and 6) but also in clusters that represented connective tissue (cluster 1) and stroma (cluster 0); histological regions in which presence of uncommitted progenitors is not expected during normal nephrogenesis. Analysis of the DE and GO sets of the SIX2+CITED1+ spots in the hFK versus WT revealed that the SIX2+CITED1+ spots of the WT (specifically in the WT blastema clusters 4 and 6) were highly enriched in pathways related mainly to muscle differentiation and regulation of the immune system (Dataset S#9, Supporting Information). They also showed clear downregulation of pathways related to renal development (Figure 6C), thus highlighting that even if these spots express SIX2 and CITED1, they are intrinsically different in the hFK compared to WT.

Since regulation of SIX2 and CITED1 expression is key to guiding proper renal development,^[^ ^2^, ^3^ ^]^ we investigated the correlation between SIX2 and CITED1 expression in our samples (Figure 6D). Using the Pearson correlation coefficient, we determined that in the hFK (cluster 5) and WTs (clusters 4 and 6) there is a significant correlation between the expression of SIX2 and CITED1. In the hFK and WT#12, the correlation was positively linear (clusters 5 and 6). However, although in WT#3 (cluster 4) we identified a significant inverse correlation, the number of spots detected for this sample was limited, therefore the interpretation of this correlation requires further investigation.

Next, we also used ST to identify spots representing the committed NP population (*SIX2+CITED1*‐ cells (Figure S22 and Dataset S#9, Supporting Information). In the hFK, the spots with committed progenitors were mainly present in cluster 3, characterized by developing nephrogenic structures, while in WT, these spots were present in different clusters (in addition to the blastema clusters 4 and 6). Analysis of DE and GO sets showed that the committed progenitors in the hFK are indeed committed towards renal differentiation in contrast to WT, where the nephrogenic differentiation pathways were not expressed (Figure S22, Supporting Information). Of note, even if the majority of the *SIX2+CITED1*‐ spots were detected in cluster 3, ST also determined the presence of these committed progenitors in cluster 5 in regions that marked the early nephrogenic structures (renal vesicles) and not within the cap mesenchyme, where only *SIX2+CITED1*+ cells reside. Interestingly, the GO set analysis showed that even if the *SIX2+CITED1*‐ spots of clusters 3 and 5 share a nephrogenic signature, the cells in cluster 5 showed enrichment of earlier nephrogenic differentiation pathways (like metanephric mesenchyme regulation and mesenchymal‐epithelial transition) versus cluster 3 representing more differentiated pathways like blood vessel development or anatomical structure morphogenesis (Figure S22 and Dataset S#9, Supporting Information).

These ST observations highlighted that even if spots were identified by the same pattern of genes (*SIX2* and *CITED1*), their transcriptomic profile was different based on their morphological context.

Next, we used ST to identify spots characterized by uncommitted NP (*SIX2+CITED1*+) and committed (*SIX2+CITED1*‐) in relation to *ITGβ1* and *ITGβ4* expression (Figure S23 and Dataset S#9, Supporting Information). In hFK, the *SIX2+CITED1+ ITGβ1*+ or *ITGβ4*+ spots are localized in the CM (cluster 5) and the *SIX2+CITED1‐ITGβ1*+ or *ITGβ4*+ spots are present in both cluster 5 and 3; possibly suggesting that expression of these ITGs could be correlated with *SIX2* and *CITED1*.

DE gene expression in the hFK versus the WT samples of the *SIX2+CITED1*+ spots or *SIX2+CITED1‐ITGβ1*+ spots was represented in the hFK by pathways related to mainly to renal nephrogenesis in addition to the regulation of cell cycle, cell division, mitosis, and ECM reorganization. A very similar signature was also evident in the hFK in the spots (*SIX2+CITED*+ or *SIX2+CITED1*‐) characterized by the presence *ITGβ4*+. Transcriptomics (a pathways analysis) of these spots in the WT patient sample was different: in WT#12 gene expression identified pathways related to a commitment towards differentiation while in WT#3 gene expression was more representative of pathways of immune‐response and self‐renewal; but none of these spots (both in WT#3 and WT#12) were committed towards nephrogenesis.

One possible interpretation of this pattern in WT spots could suggest that in WT cells are not committed toward a mature (terminally differentiated) renal fate, despite the expression of *SIX2* and *CITED1*. This interpretation is supported by the absence of expression of the major regulator of renal development, *WNT*,^[^ ^3^, ^66^ ^]^ and the presence of *FGF14* (member of the FGF family, a major signaling pathway of renal development involved in self‐renewal,^[^ ^2^, ^3^ ^]^ Figure S24A–C, Supporting Information) in WT#3, for example. Furthermore, high expression of *SIX2* in WT#12 could also explain the prevalence of muscle‐like tissue since *SIX2* is a well‐recognized factor during muscle differentiation.^[^ ^66^ ^]^ The inability to lose *SIX2*, accompanied by a push toward differentiation caused by the loss of *CITED1*, may lead to aberrant maturation toward skeletal muscle phenotypes.^[^ ^68^ ^]^ On the contrary, in the WT#3, the unbalanced presence of CITED1 prevents differentiation, although a few cells appear to acquire a cardiac‐like phenotype, possibly driven by the high expression of CITED1, which normally promotes cardiac differentiation.^[^ ^69^ ^]^

In summary, ST data (based on gene expression) suggested (as the in vitro data which are based on ITG activity) that ITG*β*1 and ITG*β*4 are crucial to prime SIX2+CITED1+ and SIX2+CITED1‐ cells toward differentiation during normal development. In WT this process is altered: NP is maintained in a self‐renewal state or, if primed towards a differentiation process, they are not pushed toward nephrogenesis.

## 3 Discussion

This study provides an extensive characterization of the WT SIX2+CITED1+ cells and, for the first time, their spatial gene profiles. The presence of NP and the expression of SIX2 or CITED1 in WT tumor cells has been confirmed^[^ ^12^, ^13^, ^14^, ^15^ ^]^ but unlike previous studies of human WT NP/CSC in which limited subtypes of WT were examined,^[^ ^70^ ^]^ or analysis was performed in chemotherapy‐treated samples,^[^ ^70^, ^71^ ^]^ or focused only on SIX2+ committed NP,^[^ ^13^ ^]^ our study incorporated several unique design features. First, uncommitted NP expressing both SIX2 and CITED1 were derived from several WT samples. Second, naïve SIX2+CITED1+ cells from hFK served as a critical reference (control). Third, the WT samples analyzed were not exposed to any chemotherapy, which is well known to alter CSC's expression pattern and differentiation status.

Our analysis, based on the developmental biology of the human kidney^[^ ^2^ ^]^ shows that SIX2+CITED1+ cells present with WT CSC characteristics. Limiting dilution experiments showed that when these cells are injected into immunodeficient mice, they form xenografts that recapitulate histologic features of the tumor of origin, demonstrate metastatic potential, and resistance to a chemotherapeutic drug. Further, pseudotime trajectory analysis points to SIX2+CITED1+ cells as the root cells that give rise to WT. We also demonstrated that WT SIX2+CITED1+ cells differ from hFK in the expression of genes regulating self‐renewal, commitment/differentiation, and proliferation, likely changing the ultimate fate of the cells and preventing normal depletion of NP from the nephrogenic niche before birth. We also found a signature of muscle/stroma differentiation in two clusters composed mainly of WT SIX2+CITED1+ cells. The same signature was found in more differentiated cells in the tumor of origin and the tumor generated by the cells after transplantation.

Other methods of selecting nephrogenic CSC (for example, NCAM1+ALDH1+ cells^[^ ^70^, ^72^ ^]^) have not clearly defined their uncommitted states, such as coexpression of SIX2 and CITED1. NCAM1+ALDH1+ cells can generate WT xenografts with triphasic morphology (even at lower concentrations) only if isolated from propagating WT fragments but do not reliably generate xenografts if freshly isolated from WT tissue and transplanted (30%‐10% success rate).^[^ ^36^, ^37^, ^70^, ^72^ ^]^ Moreover, these cells lose xenograft generation capacity completely when cultured.^[^ ^70^ ^]^ We used ST data to identify spots positive for *NCAM1* and *ALDH1* (transcript variant *ALDH1A2* was used for analysis based on high expression of this variant in many WT as indicated in https://target‐data.nci.nih.gov/Public/WT/mRNA‐seq. *ALDH1A2* is also expressed in high‐risk WTs^[^ ^73^ ^]^) and compared these spots with the *SIX2* and *CITED1* positive spots shown in Figure 6A (Figure S25, Supporting Information). In the hFK, *NCAM1+ALDH1*+ spots were highly expressed in the developing structures (cluster 3); in WT, these spots were not always present in the blastema portion if compared with the *SIX2+CITED1*+ cells. Even if the comparison of these putative classes of CSC needs more studies (not the focus of this work), our preliminary ST analysis suggests that the *NCAM1+ALDH1*+ cells might represent a more committed population of cells compared to the *SIX2+CITED1*+ cells, in addition to the observation that NCAM1 is expressed in cells mainly expressing SIX2 during the first stages of epithelization and not in uncommitted NP.^[^ ^74^ ^]^ This may be why the generation of xenografts from NCAM1+ALDH1+ cells obtained from primary digested tumors is difficult.

We defined that LAM511 and ITG*β*1 and ITG*β*4, the major LAM511 binding integrins, are crucial to prime uncommitted NP (SIX2+CITED1+ cells) and committed NP (SIX2+CITED1‐ cells). LAM511 was a rational candidate to explore since it is involved in the maintenance of pluripotency in other systems.^[^ ^55^ ^]^ When SIX2+CITED1+cells are cultured on LAM511, expression of SIX2 and CITED1 is maintained for prolonged periods, both facilitating in vitro study of normal nephrogenesis and offering, for the first time, a stable in vitro system for studying the WT CSC.

We also confirmed that hFK regions with uncommitted *SIX2+CITED1*+ NP cells also express *ITGβ1* (or *ITGβ4*) and are mainly found in the nephrogenic zone, while histologic areas with committed (*SIX2+CITED1*‐) cells expressing *ITGβ1* are found in developing structures. In WT, areas with uncommitted and committed cells and *ITGβ1* expression are found across blastema and stroma with no specific histological characteristics suggestive of a nephrogenic niche. Unlike hFK, none of the WT clusters express *WNT9b* or *WNT7b*, the most important genes for initiating renal differentiation.^[^ ^3^, ^75^ ^]^ *WNT9b* secreted from cells at the tip of the UB induces the formation of the renal vesicles, the first step of nephrogenesis.^[^ ^3^ ^]^ These results support the idea that proper nephrogenic differentiation requires a specific pattern of ITG*β*1 and ITG*β*4 expression in both uncommitted and committed NP. This nuanced regulation of differentiation is lost in WT CSC, leading to the loss of the signaling to receive proper instruction to complete differentiation, as evidenced by the dispersed irregular renal structures in WT histology.

This study also correlated gene expression patterns with histological features using spatial transcriptomics. Analysis of the ST data, supported by bulk and sc‐RNAseq data, clearly revealed that the transcriptional profiles of the WT samples have a paucity of genes involved in later steps of renal differentiation, correlating with the histologic absence of mature kidney structures like tubules or glomeruli. WT areas outside blastemic foci also contained SIX2+CITED1+ cells and included clusters primed for non‐renal fate, suggesting that some WT CSC can deviate from normal renal differentiation. Based on our analysis, we think that in some instances in WT, NP seems capable of commitment to differentiation but incapable of the typical mesenchymal‐to‐epithelial transition of renal differentiation. Alternatively, induction of NP to undergo nephrogenesis could be deficient, and in the absence of WNT signaling and the presence of high FGF expression, cells seem fixed in an uncommitted pre‐renal state.^[^ ^1^, ^3^, ^76^ ^]^ We believe these different unbalanced NP gene expression patterns have important implications in guiding our understanding of tumor initiation and progression since different mutations can lead to the development of different specific‐subtype of WT.^[^ ^10^ ^]^

Most importantly, our studies show that histologically similar blastema regions characterized by the expression of *SIX2* and *CITED* from WTs had very different gene expression profiles. We identified genes predominantly expressed in unfavorable WT blastema (like *CLEC4M*, histologically validated in multiple WT of the same subtype classification) that may ultimately be useful in deeper molecular characterization of WT for purposes of staging and prognosis.

In conclusion, highlights of this study are that i) SIX2+CITED1+ cells are the nephrogenic CSC at the origin of WT, ii) an interplay between ITG*β*1 and ITG*β*4 regulates NP state, and iii) important differences in gene expression distinguish WT samples even in histologically similar regions. Given the heterogeneity of WT from sample to sample, we recognized that more studies are needed to characterize WT fully. Nevertheless, we believe that our extensive transcriptomic studies, together with in vivo and in vitro experiments, might help the development of novel strategies, such as manipulating CSC‐ECM‐niche interaction signaling or targeting the nephrogenic signature in WT CSC that could limit cancer development or spread.

## 4 Experimental Section

### Sample Collection and Cell Suspension

Wilms tumor (WT) surgical explants were collected after informed consent under a protocol approved by the IRB of CHLA. Human fetal kidney (hFK) samples from elective terminations at 10–20 weeks gestational age (WGA) were obtained from the CHLA Tissue Bank and approved by the CHLA and USC IRB. WT samples were provided to the lab after examination by the CHLA Pathology Department (Table 1). WT staging was determined by the CHLA Pathology department per Children's Oncology Group (COG) staging guidelines^[^ ^9^ ^]^ and standard CHLA clinical practice.

Tumor and hFK samples were transported on ice at 4 °C in RPMI‐1640 (Gibco, #11875093) and processed within 1–6 h. Portions of samples were fixed in 4% paraformaldehyde (Santa Cruz Biotechnology, #sc‐281692), Tissue‐Tek O.C.T. Compound (Sakura Finetek, #4583), or stored in radioimmunoprecipitation assay (RIPA) buffer supplemented with protease and phosphatase inhibitors (ThermoFisher Scientific) for western blotting or used fresh.

Cell suspensions were prepared as previously published.^[^ ^2^ ^]^ After mechanical dissociation, cells were digested with 125 U mL^−1^ collagenase I (Worthington, # LS004197) in RPMI‐1640 (Gibco, #11875093) at 37 °C for 35 min, then passed through a 100 µm cell strainer and a 40 µm cell strainer (Corning, #352360, 352340) with washes of 1x PBS (Gibco, # 14190144). Undigested glomeruli and tubules from hFK samples did not pass through the filters. The filtrate suspension was then centrifuged at 1500 rpm for 5 min and erythrocytes were eliminated using a red blood cell lysis kit (Miltenyi Biotec, #130‐094‐183). Aliquots of isolated cells were stored in CryoStor cell cryopreservation medium (Sigma‐Aldrich, #C2874) for later use and the rest were used for flow cytometry analyses, cell isolation using Smartflare RNA probes, scRNA‐seq, bulk RNA‐seq, or transplantation into NOD/SCID mice (xenograft studies) and in vitro experiments.

### Flow Cytometry Analysis of SIX2+CITED1+ Cells from hFK and WT

hFK and WT SIX2+CITED1+ cells were isolated using SIX2‐Cy5 and CITED1‐Cy3 Smartflare RNA probes (AuraSense LLC, SIX2_CY509092019, and CITED1_Cy3012020) following manufacturer's instructions as previously reported.^[^ ^2^, ^16^ ^]^ Cells were incubated overnight (O/N) at 37 °C with both RNA probes diluted 1:20 in PBS and then diluted (25 µL mL^−1^) in RPMI‐1640 (Gibco, #11875093) supplemented with 5% fetal bovine serum (FBS, Gibco, #26140079), and 0.2% antimicrobial agent Primocin (InvivoGen, #ant‐pm‐1). Cell sorting was performed using a FacsAria sorter (BD Biosciences). Flow cytometry analysis was performed using a FacsCanto flow cytometer (BD Biosciences). For this purpose, cells were fixed in 4% paraformaldehyde (Santa Cruz Biotechnology, #sc‐281692) for 10 min and permeabilized with 0.05% saponin. Cells (10^7^/100 µl) were blocked in a human IgG 1X solution (Sigma, #I2511) for 10 min and incubated with antibodies (**Table** **2**) conjugated with Zenon labels (Thermofisher, #Z25002, Z25308, and Z25055). The analysis was done on a BD FACSDiva 5.0.1 flow cytometry system. The gating strategy was performed as described in Figure S3 (Supporting Information). Histogram plots and sorting charts were obtained using FlowJO software.

**Table 2:** List of antibodies used in the experimental procedures (IF: immunofluorescence, WB: Western blot)

Antibody	Company	Dilution
CITED1	Abnova # H00004435‐M03	1:100 IF 1:500 WB
Vimentin	Abcam # 92547	1:100 IF
Pan cytokeratin	Abcam # 6401	1:150 IF
SIX2	Proteintech Group – 11562‐1‐AP	1:100 IF 1:500 WB
Integrin beta 1	HycultBiotech #HM2033	In vivo
Integrin beta 4	Novus Bio. NBP2‐34507	In vivo
B‐catenin	Cell Signaling Tech. #8480	1:1000 WB
Non‐phospho (active) *β*‐catenin	Cell Signaling Tech. #8814	1:1000 WB
Phospho‐p44/42 MAPK (Erk1/2) (Thr202/Tyr204)	Cell Signaling Tech. #9101	1:1000 WB
p44/42 MAPK (Erk1/2)	Cell Signaling Tech. #9102	1:1000 WB
Phospho‐Akt (Thr308)	Cell Signaling Tech. #9275	1:1000 WB
Akt	Cell Signaling Tech. #9272	1:1000 WB
Phospho‐Akt (Ser473)	Cell Signaling Tech. #9271	1:1000 WB
Phospho‐GSK‐3*β* (Ser9)	Cell Signaling Tech. #9336	1:1000 WB
GSK‐3*β* (27C10)	Cell Signaling Tech. #9315	1:1000 WB
Cyclin D1	Abcam # 16663	1:1000 WB
LEF1 (C12A5)	Cell Signaling Tech. #2230	1:1000 WB
Phospho‐p70 S6 Kinase (Thr389) (108D2	Cell Signaling Tech. ##9234	1:1000 WB
p70 S6 Kinase (49D7)	Cell Signaling Tech. #2708	1:1000 WB
ITGB1	Cell signaling # 4706	1:1000 WB
B‐actin	GeneTex # GTX109639	1:1000 WB
Anti‐integrin beta 1 antibody [EP1041Y]	Abcam # 52971	1:100 IF
Anti‐integrin beta 4 antibody [EPR8558(2)	Abcam # 168386	1:100 IF
Histone H3 (3H1)	Cell Signaling # 9717	1:1000 WB
Anti‐mitochondria antibody	Sigma‐Aldrich # MAB1273	1:50 IF
CLEC4M polyclonal antibody	Proteintech Group # 22003	1:50 IF
Anti‐mouse	Life Technology # A31570	1:500 IF
Anti‐rabbit	Life Technology # A31572	1:500 IF
Anti‐goat	Life Technology # A21432	1:500 IF

### In Vitro Culture of SIX2+CITED1+ Cells

NPEM media^[^ ^2^ ^]^ was used in all in vitro cultures of hFK and WT SIX2+CITED1+ cells. To assess expression of SIX2 and CITED1 after culture on different ECM coatings, cells were plated on tissue culture plates (Corning, #CLS3516) or 12‐well Chambered Cell Culture Slides (Falcon, #354108) coated with either Matrigel (Corning Matrigel Growth Factor Reduced, #354230), laminin 511 (Laminin iMatrix‐511, #AMS.892 012), collagen 1 (Corning, #354236), fibronectin (ThermoFisher, #PHE0023) or collagen 16 (MyBioSource, #MBS2031389) following manufacturer's instructions for 5 or 28 d. Samples were then fixed and processed for either flow cytometry or immunofluorescence staining to detect expression of SIX2 and CITED1.

### In Vitro Integrin Blockage

To assess expression of SIX2 and CITED1 after integrin neutralization, hFK‐SIX2+CITED1+ cells were cultured on laminin 511 with or without the addition of either anti‐ITG*β*1 antibody (1ug/ml, clone BV7, Hycult Biotech, #HM2033b), 0.2 × 10^−3^ m MnCl_2_ (Sigma, #M‐3634), 0.6 µg mL^−1^ 9EG7 (BD Pharm, #553715) or 0.5 µg mL^−1^ anti‐ITG*β*4 antibody (clone UM‐A9, Novus Biological, #NBP2‐34507). Media and antibodies were changed every 72 h. All in vitro experiments were conducted as triplicates and repeated at least three times. Protein and cells were collected after 5 or 28 d in culture.

### In Vivo Transplantation (Xenograft) Experiments and Integrin Blockage

All animal studies were approved by the IACUC of CHLA. Immunodeficient male NOD.Cg‐Prkdscid IL2rgtm1Wjl/SzJ (NSG) mice (The Jackson Laboratory, #005557) age 2 months, were used for transplantation studies. Tumorigenicity of freshly isolated or cultured WT‐SIX2+CITED1+ cells from primary tumor cells or xenografts generated from SIX2+CITED1+ WT cells was tested. Limiting dilution studies were performed to examine the tumor initiation ability of freshly isolated WT‐SIX2+CITED1+ cells by injecting different numbers of cells 1. subcutaneously: 1000 (WT#8); 10000 (WT#8), 10000 (WT#8 1^st^ generation xenograft), 250000 (WT#8), 500000 (WT#13 and WT#8), 1 × 10^6^ (WT#13 and WT#8), 2 × 10^6^ (WT#11), 2.5 × 10^6^ (WT#13), 4 × 10^6^ (WT#8), and 6 × 10^6^ (WT#8) or 2. Intrarenal: 1000 (WT#8), 1000 (WT#8 2^nd^ generation xenograft), 10000(WT#8), 10000 (WT#8 2^nd^ generation xenograft). As a negative control, freshly isolated hFK‐SIX2+CITED1+ cells, 500 000 (WGA 17.4) and 1 × 10^6^ (WGA 17.5) were used, and as positive control, 6 × 10^6^ WT‐TOT (WT#6) cells. Prior to subcutaneous injection, all cells were suspended in 100 µL Matrigel Growth Factor Reduced (Corning, # 354230) or for intrarenal injection in 100 µL of PBS and then injected subcutaneously below the shoulder blades or intrarenal in the left kidney. After injection, mice were observed for tumor growth weekly, then twice a week once tumor was detected. Tumor volume was estimated using caliper measurements calculated by the modified ellipsoidal formula. Once the subcutaneous tumors diameter reached 1.5 cm (maximum size allowed by IACUC) the mice were euthanized, tumors removed and processed for histology. Intrarenal tumors were removed after 4 months. For scRNA‐seq of xenografts, tumors were removed and digested as described above, then Mouse Cell Depletion Kit (Miltenyi Biotec, #130‐104‐694) was used to remove mouse cells following manufacturer's instructions. ITG*β*1 neutralizing antibody (0.4 mg/kg/72 h, clone BV7, Hycult Biotech. # HM2033b), ITG*β*4 neutralizing antibody (0.4 mg/kg/72 h, Novus Biological, c# NBP2‐34507), or vincristine 450 µg kg^−1^ (vincristine sulfate, NDC PACKAGE CODE 61703‐309‐16) was administered intraperitoneally in 100 µL PBS, every 4 d once a 0.2 cm tumor was detected (4–6 weeks after initial cell transplant). A total of 78 mice were used for the in vivo studies.

### Immunocytochemistry and Histochemistry

Histological, immunohistochemistry (IHC), and immunofluorescence (IF) analyses were performed respectively on paraffin‐embedded hFK, WT, xenograft 4 µm sections (Leica, Rotary Microtome RM2235) and on cells cultured in chamber slides following fixation with 4% paraformaldehyde (Santa Cruz Biotechnology c# sc‐281692). Periodic acid‐Schiff (Sigma‐Aldrich), Sirius Red/Fast Green FCF (Sigma, #365548) and hematoxylin & eosin (Abcam, #245880) staining were performed following manufacturer's protocols. Images of WT and hFK histology were processed with a Leica DMI6000B equipped with a 5×/0.12 N PLAN Ph0 lens and DFC295 color camera (Leica Microsystems). Tiles were acquired with 10% overlap, shading‐corrected and stitched together with the Mosaic Merge function using LAS X software. For IF analysis, tissue sections and cells were permeabilized with 0.1% Triton X100 in PBS for 10 min. Additional heat‐mediated antigen retrieval using a citrate‐based antigen unmasking solution (Vector Labs, #H‐3300‐250) was performed on tissue samples. All samples were blocked in 5% bovine serum albumin (BSA, Jackson ImmunoResearch Lab #001‐000‐162) in PBS for 30 min prior to 1 h incubation at room temperature or O/N incubation at 4 °C with primary antibodies (Table 2) diluted in 2.5% BSA in PBS. Samples were then incubated for 30 min with secondary anti‐mouse, anti‐rabbit or anti‐goat AlexaFluor antibodies (Table 2) and mounted with DAPI mounting medium (Vector Laboratories, #H‐1200). For IHC analysis of CLEC4M, the endogenous peroxidase activity was blocked by incubation in PBS with 3% H2O2 (Sigma, #H1009) for 10 min. Additional heat‐mediated antigen retrieval using a citrate‐based antigen unmasking solution (Vector Labs, #H‐3300‐250) was performed. All samples were blocked in 5% bovine serum albumin (BSA, Jackson ImmunoResearch Lab #001‐000‐162) in PBS for 30 min prior to 1 h incubation at room temperature or O/N incubation at 4 °C with primary antibody, followed by 30 min incubation with ImmPRESS HRP Universal Antibody (ImmPRESS, #MP‐7500). Samples were visualized with ImpactDAB Kit (Vector Laboratories, #SK‐4100) followed by counter‐staining with hematoxylin. Images were acquired with either a Leica DM5500 B Microscope System and composition of whole images was performed with Photoshop DC (Adobe), or by confocal microscopy (Zeiss 710 microscope) and processed using the ZEN10 software.

### Western Blotting

Western blotting for protein quantification was performed as previously published.^[^ ^16^ ^]^ Membranes were blocked for 1 h at RT in either 5% skim milk (w/v) or 5% BSA (w/v) in tween‐20 tris‐glycine buffer solution (TBS‐T), depending on the primary antibody used, and probed with primary antibodies at 4 °C O/N. Horseradish peroxidase (HRP)‐conjugated secondary anti‐rabbit and anti‐mouse antibodies (Sigma‐Aldrich) were used at 1:20000 and 1:30000 dilutions respectively in 2.5% skim milk in TBS‐T. Membranes were developed using the SuperSignal West Femto Western Blotting detection reagents (Thermo Scientific, #34096) or SuperSignal West Pico (Thermo Scientific, #34577) and impressed on Biomax Light Films (GE Healthcare, #66302). Nuclear fraction protein isolation was performed using a Nuclear Extraction Kit (Abcam, #113474) following manufacturer's protocols. Data from 3 independent experiments were quantified by densitometry (all normalized against a housekeeping gene, *β*‐actin or H100).

### qRT‐PCR

qRT‐PCR was performed as published.^2,16^ RNA was extracted using RNeasy Micro Kit (Qiagen, #74004) following manufacturer's instructions and quantified with the Nanodrop system (Thermo Scientific, Waltham, MA). cDNA was obtained using the RT2 First Strand Kit (SABiosciences, QIAGEN, Valencia, CA) following manufacturer's instructions. The cDNA of each sample was then added to the KAPA SYBR Green Fast qPCR Master Mix (Kapa Biosystems #KK4610) and run on a Roche Light Cycler 480 (pre‐incubation: 95 °C, 3 min; amplification: 40 cycles of 95 °C 10 s, 60 °C 20 s, 72 °C 1 s; melting curve: 95 °C 5 s, 65 °C 1 min, 97 °C 5/°C; cooling 40 °C 10 s. Fold change as a measure of relative gene expression was calculated using the ΔΔCt method.

Primers:

### LncRNA H19

F:TCAGCTCTGGGATGATGTGGT R:CTCAGGAATCGGCTCTGGAAG.

### SIX2

F:CCAAGGAAAGGGAGAACAACG R:GCTGGATGATGAGTGGTCTGG

### CITED1

F:CTACTCCAACCTTGCGGTGAA R:CCTATTGGAGATCCCGAGGAA.

### Primer: 18S

F:AAATCAGTTATGGTTCCTTTGGTC R:GCTCTAGAATTACCACAGTTATCCAA.

### Propidium Iodide Staining

hFK and WT samples were used to assess cell cycle phase distribution of SIX2+CITED1+ and SIX2+CITED1‐ cells. Briefly, total digestates of hFK and WT samples were suspended in 1 mL PBS (1 million cells) and incubated for 15 min in ‐20 °C ethanol while vortexing. Next, cells were centrifuged and incubated for 15 min in 5 mL PBS. Cells (10^7^ cells/100 µL) were blocked in a human IgG 1× solution for 10 min and incubated with antibodies against SIX2 and CITED1 conjugated with Zenon labels (Life Technology, #647R‐Z25308 and 488M‐Z25002,) for 1 h. Cells were then washed and counterstained with 1.5 × 10^−6^ m propidium iodide – FluoroPure Grade solution (Thermo‐Fisher, #P21493). Flow cytometry analysis of propidium iodide staining and co‐staining for SIX2 (Alexa‐Fluor APC) and CITED1 (Alexa‐Fluor 488) was performed on a FacsCanto BD Biosciences instrument.

### Bulk RNA‐Seq

RNA extraction was performed immediately after sorting of SIX2+CITED1+ cells (passage 0) using the RNeasy Micro Kit (Qiagen, #74004) following manufacturer's recommendations. After cDNA production and construction of DNA libraries, the samples were run on an Illumina NextSep500 sequencer. Differential gene expression was analyzed using ERCC ExFold probes with the Remove Unwanted Variation R/Bioconductor software package combined with edgeR^2^. WT datawere deposited in Gene Expression Omnibus (GEO) under accession number (GEO for WT: #GSE176342 and GEO for hFK: #GSE74450) and processed as previously described.^[^ ^2^ ^]^ Differentially expressed (DE) genes between samples were determined by calculating fold change using the Reads Per Kilobase of transcript, per Million mapped reads (RPKM) values. DE genes with fold change >1.5 or ←1.5, for upregulated and downregulated genes respectively, were considered for downstream analysis using the Gene Ontology (GO) resource and Ingenuity pathway analysis (IPA).

### Single Cell Analysis: scRNA‐Seq

ScRNA‐seq was performed on:

1) SIX2+CITED1+ cells from a 16 WGA hFK
2) SIX2+CITED1+ cells from WT#8
3) xenograft generated in mice from cultured SIX2+CITED1+ cells (Passage 6) from WT#8
4) xenograft generated in mice from freshly isolated SIX2+CITED1+ cells from WT#8
5) total cells from WT#8 (WT‐TOT).

DAPI 1 µg mL^−1^ staining solution was used to sort out dead cells prior to library construction. Single cells were captured on a 10x Genomics Chromium system using a 10× Genomics Single Cell 3’ Gene Expression kit v2, (10× Genomics, # PN‐120237) with the target output of 3000 cells per sample. Single‐cell libraries were constructed according to the manufacturer's protocol. Final library concentrations were determined using a Qubit High Sensitivity DNA assay Kit (Thermo Fisher Scientific, #Q32854), and library qualities were verified using the Agilent Bioanalyzer High Sensitivity DNA Kit (Agilent, # 5067‐4626).

Final sequencing libraries were run and analyzed on an Illumina HiSeq 4000 sequencing system, PE150, and 625 M total reads/lane (QuickBiology). Approximately, 300 million reads/samples were sequenced. Using Partek Flow software, cells that contained fewer than 1600 expressed genes or >10% mitochondrial transcripts were removed from analyses. For each cell, the expression of each gene was normalized to the sequencing depth of the cell, scaled to a constant depth (10 000), and log‐transformed. To filter out immune cell sequences, following *k*‐means (*k* = 5) clustering, the cluster containing immune cells (based on CD45 expression) was filtered out. However, for analysis of WT‐Xe and WT‐Xe from cultured NP, mouse cells were removed prior to processing, so the elimination of CD45 cells was not necessary for those samples (Figure S11, Supporting Information). After appropriate filtering, unsupervised graph‐based clustering was performed and dimensionality reduction and visualization using the UMAP algorithm.^[^ ^77^ ^]^

DE genes for each cluster versus all other clusters were analyzed by ANOVA using the Partek software. Genes with a fold change >2 or ←2, for upregulated and downregulated genes respectively, were considered for further analysis on the GO platform or IPA. Trajectory inference analysis was performed using Monocle2 algorithm within the Partek Flow scRNAseq toolbox. The root of the trajectory for the pseudotime analysis was based on expression of the nephrogenic signature (as described by Lindström et al.^[^ ^61^ ^]^).

### Transcriptomic Analysis: Gene Ontology Enrichment Analysis, Ingenuity Pathway Analysis and Heatmap Generation

The GO resource platform (http://geneontology.org) was used to determine the enrichment of biological processes in samples of interest.^[^ ^78^, ^79^, ^80^ ^]^ DE genes (refer to bulk RNAseq, scRNA‐seq, and spatial gene expression analysis for gene expression cutoff) were uploaded onto the GO platform and GO sets were generated, powered by the PANTHER (Protein analysis through evolutionary relationships) classification system. GO sets with an enrichment score >2 and *p*‐value < 0.05 were considered significant and biologically relevant.

IPA software (Qiagen) was used to investigate biologically significant processes in samples of interest and determine the activation of canonical pathways based on bulk and scRNA‐seq data (http://www.ingenuity.com). Fold‐change values were uploaded onto the IPA software and overlaid with the global gene network in the Ingenuity Knowledge Base. Specifically, the pluripotency pathway, nephrogenic development, and WT‐associated genes were explored between samples from the bulk RNA‐seq experiments (refer to the bulk‐RNA seq section for data processing). Significant differentially regulated functional clusters or single pathways were further grouped by the indicated functional classes and compared by the enrichment score. Pathways of interest were selected as the representative for each sample and cluster if significantly enriched (*p*‐value < 0.05) or fold‐difference >1.5 patterns emerged from analysis. Z‐score >2 was considered to predict potential activation/inactivation of pathways.

Morpheus software (https://software.broadinstitute.org/morpheus) was used to generate heatmaps for visualization of either total gene expression or specific gene sets. Heatmaps were generated by uploading raw values [reads per kilobase of transcript/million reads (RPKM) for bulk RNAseq data, least squares mean (LSMean) for scRNA‐seq data]. Hierarchical clustering was applied to rows (gene lists) and columns (samples) based on a “one minus Pearson correlation” metric. Gene expression values are mapped in colors using the minimum (blue) and maximum (red) of each row (gene) independently. No cutoffs were applied when generating heatmaps.

### Spatial Gene Expression

OCT embedded 16 WGA hFK, WT#12, and WT#3 samples were sectioned to 10 µm thickness (Leica, Rotary Cryostat CM1510). RNA was isolated using the Qiagen RNeasy Mini kit (Qiagen, Hilden, Germany) and RNA quality was assessed using the 2100 Bioanalyzer (Agilent Technologies, Santa Clara, CA). All RNA tissues had RNA integrity (RIN) above 9. Tissues were then cryosectioned onto pre‐equilibrated Visium tissue optimization or gene expression slides (10× Genomics, Pleasanton, CA). These sections were then fixed in chilled methanol, stained with hematoxylin and eosin, then imaged with an Aperio AT Turbo (Leica Biosystems, Buffalo Grove, IL) at 20× magnification. Automated real‐time stitching of tiled images yielded a final image of the whole slide which was imported to image analysis software (ImageScope, Leica Biosystems).

Using visium spatial gene expression reagent kits ‐ tissue optimization user guide (10× Genomics), tissues on optimization slides were permeabilized in a time course experiment and reverse transcription was performed using fluorescently‐labeled nucleotides, resulting in fluorescent cDNA bound to the capture areas. Tissues were enzymatically removed, and fluorescence imaging was performed using a Zeiss Axio Scan.Z1 (Carl Zeiss Microscopy, White Plains, NY) digital slide scanner with a Texas Red filter set. Whole slide scanning was carried out at 20× with 150 ms exposure per image frame. Sequentially imaged frames were automatically stitched by the data acquisition software (Zen 3.0).

After H&E staining and imaging of tissue sections on gene expression slides, the sections were permeabilized for 18 min to release poly‐adenylated mRNA from overlying cells onto the capture areas of the slide. (A permeabilization time of 18 min resulted in the maximum fluorescence signal with the lowest signal diffusion.) Following the manufacturer's user guide (10× Genomics), the bound mRNA was then reverse transcribed, resulting in spatially barcoded, full‐length cDNA. Second‐strand synthesis was performed, followed by denaturation and transfer of the cDNA from the slide to a PCR tube. qPCR (KAPA SYBR FAST qPCR Master Mix, Roche Sequencing and Life Science, KAPA Biosystems, Wilmington, MA) was used to determine the number of cDNA amplification cycles required. After cDNA amplification, fragment analysis of the cDNA was performed on a 4200 TapeStation (Agilent Technologies). Library construction, performed on a portion on the cDNA, consisted of enzymatic fragmentation of the cDNA, end‐repair, and A‐tailing, followed by double‐sided size selection. Libraries were sequenced on a NovaSeq 6000 using a custom paired‐end sequencing protocol, consisting of: read 1, 28 cycles; index 1, 10 cycles; index 2, 10 cycles; read 2, 90 cycles.

Using the Visium 10× Genomics Platform we generated spatial maps of gene expression in favorable and unfavorable WT and compared them with the hFK map. Three samples (WT3, WT12 and hFK) were sequenced at the sequencing depths of 288 M, 696 M and 352 M, and detected 1766, 3735, and 1774 spots in the samples, respectively. This corresponded to a median of unique molecular indices (UMIs) per spot of 6805, 15 369, and 12 442, and a median of genes per spot of 3123, 4826, and 5326.

### ST Data Analysis

Raw sequencing data were demultiplexed and converted to fastq format by using bcl2fastq v2.20. Space Ranger software v1.0.0 (10× Genomics) was used for read alignment, tissue detection, fiducial detection, and barcode/UMI counting with pre‐defined default parameters. Briefly, raw reads were aligned to the human reference genome and transcriptome, then reads with the same barcode, UMI and gene annotation were grouped for UMI counting. For image processing, the slide barcoded spot pattern was manually aligned to the input slide image. Then, tissue and background in the slide image were discriminated. Finally, a UMI count matrix was generated consisting of the gene identities as rows and spatial barcodes as columns.

Seurat v3.2 in R v3.3.1 was used for advanced downstream data analysis. The matrices were loaded into Seurat to create a Seurat object, then normalized using sctransform^[^ ^81^ ^]^ in order to account for variance in sequencing depth across data points. To obtain 2D projections of the population dynamics, principal component analysis (PCA) was first run on the normalized gene‐barcode matrix of the top 5000 most variable genes, to reduce the number of features. These genes were identified based on their mean and dispersion as described by Macoscko et al.^[^ ^82^ ^]^ After PCA, dimensionality was reduced by uniform manifold approximation and projection (UMAP) technique which enabled further visualization of cells in a 2D space. Based on their relative positions in the UMAP plot, an unsupervised graph‐based clustering was performed to group cells for the clustering analysis.^[^ ^83^ ^]^ To identify molecular features that correlate with spatial location within a tissue, differential expression was performed based on pre‐annotated anatomical regions. Alternatively, features were searched exhibiting spatial patterning which models spatial transcriptomic data as a mark point process and computed a “variogram” to identify genes expression levels dependent on their spatial location. Data from each sample were visualized for exploration via Loupe Cell Browser, including generation of heatmaps. Significantly DE genes (*p*‐adj value < 0.05 and avg_log fold‐change >0.25 or ←0.25) were considered biologically relevant and used for downstream GO analysis. For Venn analysis of the ST data a *p*‐adj value < 0.05 and avg_log fold‐change >0.5 or ←0.5 was used.

### Statistical Analysis for In Vitro and In Vivo Experiments

All comparisons between groups were analyzed using either a two‐tailed unpaired Student's t‐test or Mann‐Whitney U‐test depending on the normality of distribution. Comparisons between more than two groups were performed by one‐way ANOVA with multiple comparisons or two‐way analysis of variance with Bonferroni correction for multiple comparisons, as applicable. All results are represented as the mean ± standard error of the mean. Data from in vivo experiments comparison of the probability of survival were analyzed using the Gehan‐Breslow‐Wilcoxon test. Data analysis was performed with the Prism Software (version 8.0; GraphPad). Significance was expressed as *p*‐value (*p < 0.05; **p < 0.01; *** *p* < 0.001; **** *p* < 0.0001).

## Conflict of Interest

The authors declare no conflict of interest.

## Author Contributions

L.P. and S.D.S. contributed equally to this work. A.P.: assistance with study design, significant contribution to data acquisition, preparation of the manuscript and revision; V.V. and P.A.: significant contribution to data acquisition and revision of the manuscript; M.E.T.: bulk RNA‐seq data acquisition and with BHG: provided tissue for analysis and manuscript revision; Y.W. and A.R.: analyzed ST data; S.Z.: provided WT histology/pathology analysis and classification; S.S.: ST data analysis, and preparation of the manuscript. R.E.D.F., K.V.L., and P.C.: data analysis and preparation of the manuscript; M.C.: reviewed results, drafted and edited the manuscript; L.P. and S.D.S.: overall direction of project design, data interpretation, manuscript preparation, and revision.

## Supporting information

Supporting Information

Supporting Tables

## Data Availability

The data that support the findings of this study are openly available in Gene Expression Omnibus at https://www.ncbi.nlm.nih.gov/geo/, under following reference numbers Bulk RNA‐Seq: (GEO for WT: #GSE176342 and GEO for hFK: #GSE74450), scRNAseq analysis (GSE175698), and Spatial Transcriptomic (GSE178349, GSM5388190, GSM5388191, and GSM5388192).
