# Supplementary material for: Identification and Characterization of the Wilms Tumor Cancer Stem Cell
Source: Adv Sci (Weinh). 2023 Apr 28;10(20):2206787. doi: 10.1002/advs.202206787 (PMC10369255; doi:10.1002/advs.202206787)
Supplement: Supplementary file 1 — Supporting Information [file ADVS-10-2206787-s002.pdf]

ADVANCED SCIENCE
Open Access

Supporting Information

for *Adv. Sci.*, DOI 10.1002/advs.202206787

Identification and Characterization of the Wilms Tumor Cancer Stem Cell

Astgik Petrosyan, Valentina Villani, Paola Aguiari, Matthew E. Thornton, Yizhou Wang, Alex Rajewski, Shengmei Zhou, Paolo Cravedi, Brendan H. Grubbs, Roger E. De Filippo, Sargis Sedrakyan, Kevin V. Lemley, Marie Csete, Stefano Da Sacco and Laura Perin**

Supplementary Discussion #1: Molecular signaling activation in SIX2+CITED1+ cells

Supplementary Data and Discussion for Figure 3 are summarized here.

PI3K-Akt-mTOR and MAPK/ERK pathways in the regulation of $SIX2^{+}CITED1^{+}$ cells

Integrins are the main receptors that bind cells to their extracellular matrix (ECM), mediating bidirectional signaling between cells and their immediate environment[1]. Integrin β chain activation leads to downstream activation of MAPK and PI3K pathways in many cell types[1–4]. We examined how PI3K-Akt-mTOR and MAPK/ERK pathways determine the balance between self-renewal vs. differentiation in SIX2+CITED1+cells in hFK.

Activation of ERK signaling in pluripotent cells inhibits self-renewal and induces differentiation[5,6]. During murine development, the MAPK/ERK pathway regulates the balance between the maintenance of nephron progenitors (NP) and their differentiation[7]. MAPK/ERK signaling is not critical after peritubular aggregate induction but is needed to prime the renal vesicle for nephron differentiation via the WNT signaling-mediator LEF1, the cell cycle regulator cyclin D1, and the NOTCH-ligand, JAG1[7].

Our studies represent the first report of a parallel role for MAPK/ERK signaling in human fetal NP. We showed that neutralization of ITGβ1, but not ITGβ4, led to a decrease in phosphorylated ERK (pERK) in SIX2+CITED1+ NP cells (Fig.S8A). Previous studies of ovarian cancer cells cultured on laminin substrate showed that ITGβ1 inhibition leads to ERK inhibition but no effect on AKT signaling[8]. MAPK/ERK signaling is also essential for the commitment of osteoblasts to differentiate. Lai et al. transduced human osteoblasts with Erk1 dominant negative protein, which inhibited the ERK/MAPK activity, and decreased expression and synthesis of β1, β3, and β5 integrin subunits and osteoblast differentiation[9]. These results support our finding that ITGβ1 neutralization may directly reduce ERK signaling and maintain SIX2 and CITED1 expression.

We also investigated connections of ITGβ1 and ITGβ4 to the PI3K/AKT/mTOR pathway, which modulates nephrogenic commitment vs. differentiation during renal development[10–13] and is typically activated in WT[14,15]. AKT's activation depends on the phosphorylation of either of two residues: threonine 308 (Thr308) or serine 473 (Ser473) [16]. In our in vitro studies of NP, neutralization of ITGβ1, but not of ITGβ4, resulted in a significant increase in AKT phosphorylation at Thr308 (Fig.S8B), but Ser473 phosphorylation was not affected by either neutralizing antibody. Phosphorylation at Thr308 does correlate with AKT protein kinase activity in some human cancers, promoting cell proliferation and survival[16,17]. Though no data are

available about its role during renal development, our studies suggest that AKT may play a crucial part in regulating normal nephrogenesis and maintaining NP.

The mTORC1 complex, a main downstream effector of AKT, integrates input signals from many factors involved in cell growth including growth factors, energy status, oxygen levels, and amino acids[18]. In the adult kidney, mTOR is critical for podocyte homeostasis and tubular transport[19]. During development, mTOR activity regulates the balance between self-renewal and differentiation: a reduction of mTORC1 and inhibition of PI3K lead to β-catenin-induced differentiation of (murine) NPs[20]. PI3K inhibition also blunts the glycolytic flux necessary for NP self-renewal[21]. To examine the activation of mTORC1, we investigated the phosphorylation of downstream kinase p70S6K1 (S6K1) at Thr389 in cultured SIX2+CITED1+ cells. Interestingly, neutralization of ITGβ1 resulted in a significant increase in p70S6K1 phosphorylation, while neutralization of ITGβ4 showed some increase (not significant) in p70S6K1 phosphorylation, specifically at Thr389 (Fig.S8C).

One of the primary functions of the PI3K-Akt-mTOR signaling pathway is control of cellular proliferation, largely through the regulation of c-Myc[22,23]. Notably, *MYC* was highly expressed in the self-renewing cluster 7, identified within the scRNA-seq aggregation analysis of WT and hFK SIX2+CITED1+ cells (Fig.S12I-L), as well as in cluster 12, and the proliferative cluster 2 (mainly a hFK cluster, Fig.4), along with *CCND1* (CYCLIN D1).

Cyclin D1 is essential for progression through the G1 phase of the cell cycle[24]. Pluripotent cells in particular, integrate various signals during G1 that determine whether they progress through the cell cycle and divide or withdraw from the cell cycle and differentiate. Higher expression of cyclin D1 likely indicates a short duration of G1 phase, skewing the cells toward a self-renewal state, rather than toward differentiation[25,26]. Integrin control of the cell cycle by regulating expression of cyclin D1 is well-known[27]. In our studies, ITGβ1 neutralization--but not ITGβ4 neutralization--led to a marked increase in cyclin D1 expression (Fig.S8D). An increase in cyclin D1 is consistent with the activation of S6K seen in our cells, as S6K is also an inducer of cyclin D1 expression. Activated S6K also inhibits glycogen synthase kinase 3 beta (GSK3β) activity, and GSK3β in turn, is a potent inhibitor of cyclin D1 activity. Interestingly, following ITGβ1 activation and AKT activation of NP in vitro, we did not detect a statistically significant change in GSK3β phosphorylation (only a trend in decreased expression, Fig.S8E), suggesting a limited role for GSK3β in the integrin-Akt/mTOR-Cyclin D1 axis controlling NP self-renewal.

Tight control of β-catenin activity is also essential for balancing self-renewal of the NP and induction of nephrogenesis[28,29]. During kidney development, β-catenin plays two different roles:

it acts as a mediator of Wnt4 to activate Fgf8, Pax8, and Lhx1 and initiates peritubular aggregate formation, but its suppression is required for further progression of nephrogenesis beyond induction[29]. β-catenin has also been shown to convert TCF/Lef1 factors from transcriptional repressors into transcriptional activators, ultimately promoting transcription of WNT target genes[29]. Importantly, β-catenin mutations or dysregulation are frequently found in WT, likely reflecting both its critical role in early nephrogenesis and the divergence of WT NP from normal trajectories early in nephrogenesis. Plisov et al. [30] showed that CITED1 regulates β-catenin transcription-dependent signals involved in epithelial induction by competing for binding to the transcriptional co-activator EP300[30], limiting β-catenin activation indirectly and subsequently preventing cell cycle exit and differentiation. In our in vitro studies, β-catenin expression (Fig.S8F-G) was not significantly affected by the neutralization of ITGβ1 or ITGβ4, but neutralization of ITGβ4 increased the expression of EP300 (Fig.S8H). These preliminary studies suggest that the interplay between ECM, integrins, and PI3K/Akt/mTOR pathway is critical for regulating self-renewal vs. cell cycle exit and differentiation in uncommitted NP. These findings are summarized in Fig.3J. The complex interactions contributing to the choice between self-renewal vs. differentiation of human NP require further study to enhance understanding of normal hFK development, as well as the development of WT.

Supplementary Discussion #2: GO analysis of scRNA-seq data, Spatial Transcriptomic (ST) cluster analysis, and gene expression signature in WT.

To identify transcriptional signatures that distinguish different subtypes of WT, we performed GO analysis and Spatial Transcriptomics (ST) of hFK as well as WT#12 (characterized by favorable histology) and WT#3 (characterized by unfavorable histology) To decipher the transcriptional signatures of hFK and WT SIX2+CITED1+ cells, we carried out sc-RNAseq and performed gene ontology (GO) analysis between the different clusters. We identified cluster distributions and specific patterns of gene expression. Supplementary Data and Discussion for Figures 4 and Figure 5 are summarized here.

GO analysis of scRNA-seq data

scRNA-seq was performed on SIX2+CITED1+ cells from WT#8 (stage II favorable WT) and hFK (16.6 WGA) to investigate heterogeneity. 12 distinct clusters were identified in the combined data. Results were also compared with hFK data generated by Lindström et al. [31]. GO analysis was performed on DE genes for each cluster to confirm the findings (Fig. 4F,

SupplementaryDataset#4). GO analysis highlighted that proliferative clusters 2 (hFK) and 11 (WT) are highly enriched for cell cycle regulation genes. Committed clusters 5 (WT) and 6 (hFK) are highly enriched for genes essential in the organization and regulation of tight junctions, typical of mature renal structures. hFK clusters 4, 6, and 10 exclusively express genes involved in late-stage nephrogenic commitment and differentiation, not found in WT clusters (1, 3, 5, 8, 9, and 11; Fig. 4C-D), which is consistent with the absence of fully differentiated renal structures in WT histology. Self-renewing cluster 7 (represented by both hFK and WT) was highly enriched in genes involved in epigenetic regulation (i.e. histone methylation, a hallmark of stem cell maintenance[32], and negative regulation of translation and chromatin organization, tightly regulated processes in undifferentiated cells[33]; Fig. 4F).

ST of hFK and WT

We performed and compared ST (Visium 10x Genomics) on WT#12 (stage III favorable), WT#3 (stage I unfavorable), and 16.6 WGA hFK samples. Here we describe the results of the analyses for each tissue sample separately. Discussion of the results following transcriptomic data integration of the three samples is reported in the Results section of the main manuscript.

hFK clusters identified with ST

Histological analysis of hFK revealed nephrogenic niches, developing renal structures (including primitive glomeruli, distal and proximal tubules), vasculature, and stroma (Fig.S17A). ST analysis identified 7 clusters (Fig.S17B-C, SupplementaryDataset#6). As expected, these clusters represented various stages of kidney development and spatially overlapped with morphologically recognizable developing renal structures (including the nephrogenic niche) and mature renal structures (Fig.S17B). Differences between cluster 6 and the other clusters accounted for most of the variance between clusters. GO analysis further confirmed that hFK (16.6 WGA) simultaneously expresses genes from early, maturing, and mature stages of renal development (Fig.S17D)

In hFK, cluster 0, spatially overlapping with the anatomic nephrogenic zone, expressed (almost exclusively) *PAX2*, *SALL1*, *EYA1*, *RET*, as well as *CITED1* and *SIX2.* Clusters 1 and 3 exhibit high expression of glomerulus-specific markers (*PODXL*, *NPHS1*, *NPHS2*, *WT1*, *EHD3*, *COL4A3* and *COL4A4),* and tubular markers (*SLC48A1 and TFPI)* [34]*.* Cluster 1 also expresses genes characteristic of early glomerular development (*PAX2, PAX8*). Cluster 2 identified distal tubule and loop of Henle cells, with an expression of *CLDN16*, *SLC12A1*, *MAL*, *IRX2*[34]. Cluster 4, spatially localized between nephrogenic cluster 0 and more mature clusters, expresses *JAG1*

and *LHX1*, associated with renal vesicles and developing glomeruli. Both clusters 5 and 6 highly express stromal markers (*UMOD*, *DCN*, *COL1A1*, *OGN*, *MGP*, *TAGLN* and *ACTA2),* with cluster 6 also enriched for non-glomerular endothelial markers (*CDH5*, *EHD2, PECAM1* and *CAV1*).

WT#3 clusters identified with ST

Histological analysis of WT#3 (stage I unfavorable) confirmed the presence of the three typical histologic components (blastema, tubular structures, and stroma) and a tumor capsule (Fig.S17E, SupplementaryDataset#6). Cluster specific gene expression and GO are presented in Fig.S17F-H. Taken together, the spatial mapping suggests that clusters consistent with differentiating renal tissue (1 and 2) are scattered throughout the tumor, as clusters with aberrant non-renal expression patterns (0).

In WT#3, cluster 0, histologically stroma, contains regions highly expressing stromal cell markers associated with WT and other cancers (*CTGF*, *VIM*, *NREP* and *SPARC)* [35,36], but lacks expression of renal specific genes like *CITED1*. Clusters 1 and 4 co-localize with areas of blastema histology. Cluster 1 shows expression of several early renal development markers (*PAPPA2*, *PAX2* and *MEOX1*) along with proximal tubule markers *ATP1A1* and *KCNJ15*[34,36]*,* but also muscle markers like *NACA*[37] and *CMYA5*[38,39]. Cluster 4 exhibits a signature consistent with tubule cells, with several *HSPA* and *HSPB* genes[40] and *CALCA*[34]. Cluster 2, spatially localized to the tumor capsule, is enriched for endothelial genes (*VWF*, *PECAM1*, *VCAM1)* as well as cell adhesion markers, including integrins and other ECM components, suggesting the presence of vascularization. Cluster 3, mapping to an area of tubule-like structures (and overlapping with cluster 0), does not exhibit a signature pointing to a specific renal fate but shows a weak fibrotic signature-low expression of *SPARC*, *ENO1*, *VIM,* and higher expression of *CCDC88C*, an inhibitor of the WNT/Fzd pathway critical for NP induction and differentiation[41]. GO analysis further confirmed the co-existence of different renal (cluster 2) and non-renal (clusters 1 and 4) signatures within WT#3.

WT#12 clusters identified with ST

Histological analysis of a WT#12 (stage II favorable) also identified distinct anatomic compartments, including blastema, stroma with rhabdomyomatous differentiation, connective tissue, and tubular components (Fig.S17I-J, SupplementaryDataset#6). The clusters were not spatially localized to a nephrogenic niche and did not express a specific renal differentiation signature, correlating with a histological absence of mature renal microanatomy. The definition

of spatially resolved clusters, specific gene expression and GO analysis are presented in Fig.S17J-L.

WT#12, cluster 0 is highly enriched in genes expressing matrix proteins like *COL3A1* and *COL1*[42] as well as *ELN*, *DCN* and *LUM* and muscle-specific genes including *MYBPH*, *MYL4*, *MYH8*. Muscle fibers are not uncommon in WT histopathology[43,44]. Cluster 1, which has features of connective tissue, displays an enriched expression of mitochondrial genes, with an underrepresentation of muscle development genes. Nephrogenesis genes are highly represented in Cluster 2, including *HOXA* genes as well as *SIX2*, *GDNF*, *SALL1* and *MEOX1*. GO analysis shows enrichment of gene sets relative to proliferation and cell cycle. Cluster 3, which spatially overlaps with areas of stroma and stunted tubules, could not be assigned to a specific cell type. Comparison of clusters 3 and 1 (most similar based on UMAP-based visualization of the data), indicates a stromal gene expression pattern (*TAGLN2*, *DES* and *PDGFRB)*, with lower expression of *HMGA2*, a regulator of proliferation and mesenchymal differentiation in cluster 3. Of note, HMGA2 is negatively regulated by H19 through let-7, so the absence of H19 in WT would be expected to increase HMGA2[45] and suppress epithelialization and nephron formation. Expression of cardiac muscle troponins and myosins characterizes the signature of cluster 4, confirmed by GO analysis[46,47]. Cluster 5 is highly enriched for immune cell markers, including HLAs, and monocyte markers *CD68*, *LYZ* and *CD14*. GO analysis confirmed that a muscle signature is preponderant in clusters 0, 3 and 4 while proliferation pathways are enriched in nephrogenic cluster 2.

From the integration data in Fig. 1, we also evaluated DE genes between nephrogenic clusters 5 and 3 specific to hFK, and cluster 4 specific to WT#3 (Fig.S19). WT cluster 4, compared to the more uncommitted hFK cluster 5, shows downregulation of important genes involved in activation of committed NP (Fig.S19G). Compared to cluster 3 (the more committed cluster of hFK, Fig.S6G), cluster 4 exhibits a higher expression of uncommitted genes (like CITED1). These data can be interpreted as demonstrating an ‘uncommitted NP’ profile characteristic of WT#3 (as compared to hFK). In addition, cluster 6 (specific to WT#12) showed high expression of myogenic genes (*MYL1*, *MYH3*, *MYL4*, SupplementaryDataset#7) compared to cluster 5, and higher expression of *CITED1*, *SIX2*, and *SALL3* compared to cluster 3, reflecting both a muscle differentiation-prone and more uncommitted state than cluster 3 (Fig.S19G).

Gene expression signature: WT#12 vs. WT#3

To identify genes differentially expressed in areas of WT#12 vs. WT#3 identifying the blastema, we used ST and performed analysis on aggregated data as shown in Fig.S19F. Our analysis

also identified genes that are DE in specific clusters or groups of clusters (SupplementaryDataset#7) that further distinguish WT#12 vs. WT#3.

Genes predominately expressed in the WT#3 blastema (cluster 4) were *LINC01833, FGF14, GPR39, MGAM and TDGF1*, while genes predominately expressed in the WT#12 blastema (clusters 0 and 6) were *EBF3, SYCE1, PAX3, and XIST. DDX3Y, CLEC4M, RPS4Y1 and KLK6* were exclusively DE expressed in WT#3, while *MYL1, SULT1E1, SMPX, MYH8, SALL3, COX6A2* were DE expressed only in WT#12, and only *NPHS2* and *HBG2* DE expressed in hFK. These comparisons thus unmasked some interesting new gene associations for WT#3, including DE genes in cluster 4 associated with poor prognosis in multiple other cancers, like *CLEC4M*[48,49] (Fig.S21E-L,Table 3, SupplementaryDataset#7).

Genes expressed predominately in WT#3

The WT#3 (stage I unfavorable), a male patient with a pathological diagnosis of epithelial-predominant with diffuse anaplasia tumor, with loss of chromosome 22 and chromosome 17p, and a TP53 missense mutation which is found in up to 75% of unfavorable WTs[50]. TP53 gene allows the production of P53 protein, which acts as a tumor suppressor by preventing uncontrollable cellular proliferation. The top DE genes expressed in cluster 4 (WT#3 blastemal region) were *LINC01833, FGF14, GPR39, MGAM,* and *TDGF1*. *LINC01833* is over-expressed in lung adenocarcinoma and significantly enhances the EMT process by inhibiting miR-519e-3p expression, thus promoting metastasis and invasiveness[51]. *LINC01833* is a long noncoding RNA located near the gene *SIX3*, a location suggesting that this non-coding transcript may interact with the Wnt/β-catenin pathway[52]. *SIX3* binds to the *Wnt1* promoter region resulting in repressed *Wnt1* expression in breast cancer. *SIX3* also acts as a co-repressor of *Wnt* transcription[53,54]. *FGF14* is an intracrine FGF which acts in an FGFR-independent manner regulating the function of voltage gated sodium channels[55]. In colon cancer, *FGF14* behaves like a tumor suppressor gene, inhibiting the PI3K/AKT/mTOR pathway[56]. In our in vitro studies, inhibition of the PI3K/AKT/mTOR pathway led to the upregulation of *CITED1,* which is also highly expressed in WT#3.

The Zn^{2+}-sensing G-protein coupled receptor *GPR39 is* upregulated in normal hFK and stem-like WT xenografts[57,58]. In breast cancer, high expression of *GPR39* acts as an upstream regulator of cancer cell proliferation to promote more aggressive tumors[59]. *MGAM* (maltase-glucoamylase alpha-glucosidase), is upregulated in various cancers such as oral squamous cell carcinoma[60] and is thought to promote tumor growth by altering carbohydrate metabolism through a breakdown of dietary starches and sugars into glucose[61]. Altered glucose

metabolism including glycolysis and oxidative phosphorylation are common in cancer cells[62] and GO analysis also identified several other metabolic pathway alterations in the WT#3. Teratocarcinoma-derived growth factor 1 (*TDGF1* or *CRIPTO*-1) plays important roles in stem cell and embryonic development, and in the growth (and poor outcome) of several human cancers[63], so *CRIPTO-1* overexpression likely drives tumor growth through expansion of cancer stem cells. Several investigators have proposed targeting CRIPTO-1/NODAL signaling to directly target cancer stem cells or undifferentiated stem-like tumor initiating cells[63]. Overexpression of *CRIPTO-1* is associated with lower expression of Netrin-1 (*NTN1*), part of a family of laminin-related secreted proteins that promote the reduction of vimentin and upregulation of e-cadherin[64]. *NTN1* is a key negative regulator of *CRIPTO-1*. Our analysis showed no difference in *NTN1* expression in WT#3 vs. WT#12, suggesting that the brake on *CRIPTO-1* activity via *NTN 1* may not be a major factor in differentiating WT subtypes (i.e., other controls on *CRIPTO*-1 may be operant in WT#3).

We also identified *CLEC4M, DDX3Y* and *KLK6* as exclusively expressed in WT#3. *CLEC4M* (homologue DC-SIGN), a Ca2+-dependent C-type lectin, is correlated with tumorigenesis and poor outcome in lung cancer patients (resistance to cisplatin-based chemotherapy)[49], colon/gastric cancers (promoting liver metastasis) [65,66], and hepatocellular carcinoma, where it is associated with increased microvascular invasion, larger tumor size, absence of tumor encapsulation, less tumor differentiation, lower overall survival, and increased risk of reoccurrence.[48] DEAD-box RNA helicase 3 (*DDX3*), a highly conserved family member of DEAD-box proteins and 40S ribosomal protein S4, has two homologs, *DDX3X* and *DDX3Y*[67]. *DDX3X* is located on the X-chromosome and *DDX3Y* on the Y-chromosome. We found no difference in *DDX3X* expression in WT#12 (female) and WT#3(male) explants, however *DDX3Y* was upregulated in the WT#3. High expression of *DDX3Y or DDX3X* is associated with an aggressive phenotype in human malignancies including thyroid, lung, colorectal, stomach, prostate cancers, and melanoma, but is associated with good prognosis in head and neck, pancreatic, and gastric cancers[68,69]. *DDX3* inhibits expression of cyclin-dependent kinase inhibitor p21 (also known as p21WAF1/Cip1), leading to uncontrolled cell growth[70]. In addition, the WT#3 we studied also had a TP53 mutation, which mediates the DNA damage-induced checkpoint through the transactivation of apoptosis regulators such as p21(p53-dependent G1 growth arrest) [71]. *KLK6*, a kallikrein-related peptidase protein, was also overexpressed in WT#3. *KLK6* overexpression is associated with upregulation of the EMT marker vimentin and loss of e-cadherin[72], as well as increased aggressiveness, metastasis, and poor prognosis of multiple cancers, including colorectal cancer[73].

Genes expressed predominantly in WT#12

The WT#12 (stage II favorable), a female patient with a pathological diagnosis of favorable histology with no evidence of anaplasia, with loss of heterozygosity for 11p and 3p, a deletion in 10q, and WT1 frameshift mutation along with a hotspot mutation in CTNNB1 (beta-catenin). It has been reported that a high percentage of differentiated muscle cells are found in chemotherapy-treated WT1-mutant tumors, suggesting that cells with these mutations have an intrinsic ability to differentiate *in vivo*[44]. Indeed, spatial transcriptomics data shows that WT#12 SIX2+CITED1+ or SIX2+CITED1- cells (Fig.S22) express GO terms related to muscle development. The top DE genes in clusters 0 and 6 (WT#12) were: *EBF3, SYCE1, PAX3*, and *XIST*. *EBF3* is an early B-cell factor; EBFs are DNA-binding transcription factors that regulate cellular differentiation in all three embryonic germ layers[74]. In cancers (breast, bone, lung, gastric, and colorectal cancers), *EBF3* acts as a tumor suppressor and is usually methylated, leading to tumor initiation and metastasis[75]. *EBF* also has an inhibitory effect on p300/CREB-binding protein through a direct interaction[76], which is vital for renal development. Synaptonemal complex central element protein 1 (SYCE1 or CT76) is a member of the synaptonemal complex, which links homologous chromosomes during meiosis (prophase I), and is required for initiation and elongation of the synapse[77]. Expression of *SYCE1* is associated with lung adenocarcinoma[78] and with prostate cancer reoccurrence after radical prostatectomy [79].

The transcription factor *PAX3* is an upstream regulator of myogenesis during development. *PAX3* is found in the developing kidney metanephric mesenchyme and stroma, and in WT containing WT1 mutations. Likely, *PAX3* is normally suppressed by WT1 during the mesenchymal to epithelial transition to allow nephrogenesis, but mutations in WT1 result in aberrant *PAX3* expression in some WTs, promoting a myogenic phenotype[80]. Indeed, the WT#12 examined had a WT1 frameshift mutation, and histology consistent with muscle phenotypes, and upregulated GO pathways associated with muscle development. The X inactive specific transcript (*XIST*) is the main regulator of X chromosome inactivation in mammals, and is highly expressed in the very malignant tumor, non-small cell lung carcinoma[81]. *XIST* expression promotes proliferation, migration, invasion, and EMT in these cancer cells. The WT#12 highly expressed the basic helix-loop-helix transcription factor *TWIST 1* compared to WT#3. TWIST 1 is vital for EMT progression[82,83]. *TWIST* is also critical for muscle development and may reverse muscle cell differentiation through binding and down-regulation of myogenin[84]. We also determined that *COX6A2, SALL3, MYH8, SMPX, SULT1E1*,

and *MYL1* are exclusively expressed in WT#12. *COX6A2* is a mitochondrial oxidative phosphorylation (OXPHOS) gene which promotes energy metabolism, and inhibits oncogenicity in cancer stem cells[85]. Spalt like transcription factor 3 (*SALL3*) is predominantly silenced by DNA methylation in cancer, leading to aberrant methylation of other tumor-related genes (*TET1, TET2*, and *DNMT3A*), and high disease reoccurrence[86–88]. Sulfotransferase family 1E member 1 (*SULT1E1*) is a tumor suppressor gene, known to inhibit breast cancer cell growth by inducing apoptosis (arresting cell cycle progression) and inhibiting tumor cell migration and invasion[89]. Myosin heavy chain 8 (*MYH8*) is expressed in developing embryonic, neonatal, and perinatal skeletal muscle[90]. Small muscle protein X-Linked (*SMPX*) is typically expressed in striated muscle and is vital for differentiating human skeletal myoblasts to myotubes[91]. Myosin light chain 1 (*MLC1*) is expressed by fast skeletal muscle and is necessary for the proper formation and maintenance of myofibers and muscle function[92].

Table 3: List of genes identified by ST in WT#12 and WT#3.

**Gene name**	**Full gene name**	**GeneCardID**
CDKN1A	Cyclin dependent kinase inhibitor 1A	GC06P049517
CITED1	Cbp/p300 interacting transactivator with glu/asp rich carboxy-terminal domain 1	GC0XM072301
CLEC4M	C-type lectin domain family member 4 Member M	GC19P007763
COX6A2	Cytochrome oxidase subunit 6A2	GC16M031439
CTNNB1	Catenin beta 1	GC03P041236
DDX3Y	DEAD-box helicase 3 Y-linked	GC0YP012903
DNMT3A	DNA methyl transferase 3 alpha	GC02M025228
EBF3	EBF transcription factor 3	GC10M129835
FGF14	Fibroblast growth factor 14	GC13M101710
GPR39	G protein-coupled receptor 36	GC02P133082
KLK6	Kallikrein related peptidase 6	*GC19M050958*
LINC01833	Long intergenic non-coding RNA 1833	GC02M044921
MGAM	Maltase-glucoamylase	GC07P145339

MLC1	Modulator of VRAC current 1	GC22M050059
MYH8	Myosin heavy chain 8	GC17M010390
MYL1	Myosin light chain 1	GC02M210290
NTN1	Netrin-1	GC17P009021
p21WAF1/Cip1	See CDKN1A	
PAX3	Paired box 3	GC02M222199
SALL3	Spalt like transcription factor 3	GC18P078980
SIX3	Six homeobox 3	GC02P044941
SMPX	Small muscle protein X-linked	GC0XM021706
SULT1E1	Sulfotransferase family 1E member 1	GC04M069841
SYCE1	Synaptonemal complex central element protein 1	GC10M133553
TDGF1 (Cripto-1)	Teratocarcinoma-derived growth factor 1	GC03P046576
TET1	Tet methylcytosine dioxygenase 1	GC10P068560
TET2	Tet methylcytosine dioxygenase 2	GC04P105145
WNT1	Wnt family member 1	GC12P049053
WT1	WT1 transcription factor	GC11M032365
XIST	X inactive specific transcript	GC0XM073820

References

1. E. A. Clark and J. S. Brugge, *Science*, **1995**, *268*, 233.
2. T. H. Lin, A. E. Aplin, Y. Shen, Q. Chen, M. Schaller, L. Romer, I. Aukhil, and R. L. Juliano, *J Cell Biol*, **1997**, *136*, 1385.
3. K. Kurtzeborn, H. N. Kwon, and S. Kuure, *Int J Mol Sci*, **2019**, *20*.
4. L. S. Steelman, W. H. Chappell, S. L. Abrams, C. R. Kempf, J. Long, P. Laidler, S. Mijatovic, D. Maksimovic-Ivanic, F. Stivala, M. C. Mazzarino, M. Donia, P. Fagone, G. Malaponte, F. Nicoletti, M. Libra, M. Milella, A. Tafuri, A. Bonati, J. Bäsecke, L. Cocco, C. Evangelisti, A. M. Martelli, G. Montalto, M. Cervello, and J. A. McCubrey, *Aging (Albany NY)*, **2011**, *3*, 192.
5. X. Ma, H. Chen, and L. Chen, *Exp Hematol*, **2016**, *44*, 151.
6. H. Chen, R. Guo, Q. Zhang, H. Guo, M. Yang, Z. Wu, S. Gao, L. Liu, and L. Chen, *PNAS*, **2015**, *112*, E5936.
7. A. Ihermann-Hella, T. Hirashima, J. Kupari, K. Kurtzeborn, H. Li, H. N. Kwon, C. Cebrian, A. Soofi, A. Dapkunas, I. Miinalainen, G. R. Dressler, M. Matsuda, and S. Kuure, *Stem Cell Reports*, **2018**, *11*, 912.
8. N. Ahmed, C. Riley, G. Rice, and M. Quinn, *Clin Exp Metastasis*, **2005**, *22*, 391.
9. C.-F. Lai, L. Chaudhary, A. Fausto, L. R. Halstead, D. S. Ory, L. V. Avioli, and S.-L. Cheng, *Journal of Biological Chemistry*, **2001**, *276*, 14443.
10. E. M. Yazlovitskaya, H.-Y. Tseng, O. Viquez, T. Tu, G. Mernaugh, K. K. McKee, K. Riggins, V. Quaranta, A. Pathak, B. D. Carter, P. Yurchenco, A. Sonnenberg, R. T. Böttcher, A. Pozzi, and R. Zent, *Mol Biol Cell*, **2015**, *26*, 1857.
11. J. A. Kreidberg and J. M. Symons, *American Journal of Physiology-Renal Physiology*, **2000**, *279*, F233.
12. S. Mathew, X. Chen, A. Pozzi, and R. Zent, *Pediatr Nephrol*, **2012**, *27*, 891.
13. S. Chen, E. W. Brunskill, S. S. Potter, P. J. Dexheimer, N. Salomonis, B. J. Aronow, C. I. Hong, T. Zhang, and R. Kopan, *Developmental Cell*, **2015**, *35*, 49.
14. V. Subbiah, R. E. Brown, Y. Jiang, J. Buryanek, A. Hayes-Jordan, R. Kurzrock, and P. M. Anderson, *PLoS One*, **2013**, *8*, e68985.
15. J. Peringa, W. M. Molenaar, and W. Timens, *Virchows Arch*, **1994**, *425*, 113.
16. J. R. Hart and P. K. Vogt, *Oncotarget*, **2011**, *2*, 467.
17. E. E. Vincent, D. J. E. Elder, E. C. Thomas, L. Phillips, C. Morgan, J. Pawade, M. Sohail, M. T. May, M. R. Hetzel, and J. M. Tavaré, *Br J Cancer*, **2011**, *104*, 1755.
18. M. Laplante and D. M. Sabatini, *Journal of Cell Science*, **2009**, *122*, 3589.
19. D. Fantus, N. M. Rogers, F. Grahammer, T. B. Huber, and A. W. Thomson, *Nature Reviews Nephrology*, **2016**, *12*, 587.
20. N. O. Lindström, N. O. Carragher, and P. Hohenstein, *Stem Cell Reports*, **2015**, *4*, 551.
21. J. Liu, F. Edgington-Giordano, C. Dugas, A. Abrams, P. Katakam, R. Satou, and Z. Saifudeen, *J Am Soc Nephrol*, **2017**, *28*, 3323.
22. M. Granato, C. Rizzello, M. A. Romeo, S. Yadav, R. Santarelli, G. D'Orazi, A. Faggioni, and M. Cirone, *The International Journal of Biochemistry & Cell Biology*, **2016**, *79*, 393.
23. J. Zhu, J. Blenis, and J. Yuan, *Proc Natl Acad Sci U S A*, **2008**, *105*, 6584.
24. J. P. Alao, *Mol Cancer*, **2007**, *6*, 24.
25. F. Duquesne, M. Florent, G. Roué, X. Troussard, and B. Sola, *Cell Death & Differentiation*, **2001**, *8*, 51.
26. D. Resnitzky, M. Gossen, H. Bujard, and S. I. Reed, *Mol Cell Biol*, **1994**, *14*, 1669.

27. P. Moreno-Layseca and C. H. Streuli, *Matrix Biology*, **2014**, *34*, 144.
28. P. Deacon, C. W. Concodora, E. Chung, and J.-S. Park, *Scientific Reports*, **2019**, *9*, 15915.
29. K. M. Schmidt-Ott and J. Barasch, *Kidney International*, **2008**, *74*, 1004.
30. S. Plisov, M. Tsang, G. Shi, S. Boyle, K. Yoshino, S. L. Dunwoodie, I. B. Dawid, T. Shioda, A. O. Perantoni, and M. P. de Caestecker, *J Am Soc Nephrol*, **2005**, *16*, 1632.
31. N. O. Lindström, J. A. McMahon, J. Guo, T. Tran, Q. Guo, E. Rutledge, R. K. Parvez, G. Saribekyan, R. E. Schuler, C. Liao, A. D. Kim, A. Abdelhalim, S. W. Ruffins, M. E. Thornton, L. Baskin, B. Grubbs, C. Kesselman, and A. P. McMahon, *J. Am. Soc. Nephrol.*, **2018**, *29*, 785.
32. H. Wu and Y. E. Sun, *Pediatr Res*, **2006**, *59*, 21R.
33. S. Srivastava, R. K. Mishra, and J. Dhawan, *Organogenesis*, **2010**, *6*, 37.
34. L. Chen, J. Z. Clark, J. W. Nelson, B. Kaissling, D. H. Ellison, and M. A. Knepper, *JASN*, **2019**, *30*, 1358.
35. J. W. Hellinger, F. Schömel, J. V. Buse, C. Lenz, G. Bauerschmitz, G. Emons, and C. Gründker, *Sci Rep*, **2020**, *10*, 17889.
36. M. D. Young, T. J. Mitchell, F. A. Vieira Braga, M. G. B. Tran, B. J. Stewart, J. R. Ferdinand, G. Collord, R. A. Botting, D.-M. Popescu, K. W. Loudon, R. Vento-Tormo, E. Stephenson, A. Cagan, S. J. Farndon, M. Del Castillo Velasco-Herrera, C. Guzzo, N. Richoz, L. Mamanova, T. Aho, J. N. Armitage, A. C. P. Riddick, I. Mushtaq, S. Farrell, D. Rampling, J. Nicholson, A. Filby, J. Burge, S. Lisgo, P. H. Maxwell, S. Lindsay, A. Y. Warren, G. D. Stewart, N. Sebire, N. Coleman, M. Haniffa, S. A. Teichmann, M. Clatworthy, and S. Behjati, *Science*, **2018**, *361*, 594.
37. H. Li, W. R. Randall, and S.-J. Du, *FASEB J*, **2009**, *23*, 1988.
38. M. A. Benson, C. L. Tinsley, A. J. Waite, F. A. Carlisle, S. M. M. Sweet, E. Ehler, C. H. George, F. A. Lai, E. Martin-Rendon, and D. J. Blake, *Sci Rep*, **2017**, *7*, 6312.
39. P. Hohenstein, K. Pritchard-Jones, and J. Charlton, *Genes Dev.*, **2015**, *29*, 467.
40. D. Scieglinska, W. Piglowski, M. Chekan, A. Mazurek, and Z. Krawczyk, *Histochem Cell Biol*, **2011**, *135*, 337.
41. A. B. Ekici, D. Hilfinger, M. Jatzwauk, C. T. Thiel, D. Wenzel, I. Lorenz, E. Boltshauser, T. W. Goecke, G. Staatz, D. J. Morris-Rosendahl, H. Sticht, U. Hehr, A. Reis, and A. Rauch, *Mol Syndromol*, **2010**, *1*, 99.
42. H. Sariola, P. Ekblom, J. Rapola, A. Vaheri, and R. Timpl, *Am J Pathol*, **1985**, *118*, 96.
43. A. J. Garvin, F. Surrette, D. S. Hintz, M. T. Rudisill, M. A. Sens, and D. A. Sens, *Am J Pathol*, **1985**, *121*, 298.
44. B. Royer-Pokora, M. Beier, A. Brandt, C. Duhme, M. Busch, C. de Torres, H. Royer, and J. Mora, *Cancer Med*, **2018**, *7*, 1359.
45. L. Yan, J. Zhou, Y. Gao, S. Ghazal, L. Lu, S. Bellone, Y. Yang, N. Liu, X. Zhao, A. D. Santin, H. Taylor, and Y. Huang, *Oncogene*, **2015**, *34*, 3076.
46. M. A. Sens, A. J. Garvin, S. Drew, C. D. Smith, H. B. Othersen, and D. A. Sens, *Arch Pathol Lab Med*, **1984**, *108*, 58.
47. Y. Yamada, K. Namba, and T. Fujii, *Nat Commun*, **2020**, *11*, 153.
48. L. Luo, L. Chen, K. Ke, B. Zhao, L. Wang, C. Zhang, F. Wang, N. Liao, X. Zheng, X. Liu, Y. Wang, and J. Liu, *Oncology Letters*, **2020**, *19*, 1711.
49. L.-M. Tan, X. Li, C.-F. Qiu, T. Zhu, C.-P. Hu, J.-Y. Yin, W. Zhang, H.-H. Zhou, and Z.-Q. Liu, *J Cancer*, **2019**, *10*, 6374.

50. T. D. Treger, T. Chowdhury, K. Pritchard-Jones, and S. Behjati, *Nat Rev Nephrol*, **2019**, *15*, 240.
51. Y. Zhang, W. Li, Z. Lin, J. Hu, J. Wang, Y. Ren, B. Wei, Y. Fan, and Y. Yang, *Cancer Manag Res*, **2020**, *12*, 11157.
52. J. Chen, L. Hu, J. Chen, Q. Pan, H. Ding, G. Xu, P. Zhu, X. Wen, K. Huang, and Y. Wang, *Pathol Oncol Res*, **2016**, *22*, 609.
53. O. V. Lagutin, C. C. Zhu, D. Kobayashi, J. Topczewski, K. Shimamura, L. Puelles, H. R. C. Russell, P. J. McKinnon, L. Solnica-Krezel, and G. Oliver, *Genes Dev.*, **2003**, *17*, 368.
54. R. Kumar, S. Balasenthil, B. Manavathi, S. K. Rayala, and S. B. Pakala, *Cancer Res*, **2010**, *70*, 6649.
55. D. M. Ornitz and N. Itoh, *Wiley Interdiscip Rev Dev Biol*, **2015**, *4*, 215.
56. T. Su, L. Huang, N. Zhang, S. Peng, X. Li, G. Wei, E. Zhai, Z. Zeng, and L. Xu, *J Cancer*, **2020**, *11*, 819.
57. B. Dekel, S. Metsuyanim, K. M. Schmidt-Ott, E. Fridman, J. Jacob-Hirsch, A. Simon, J. Pinthus, Y. Mor, J. Barasch, N. Amariglio, Y. Reisner, N. Kaminski, and G. Rechavi, *Cancer Res*, **2006**, *66*, 6040.
58. S. Metsuyanim, O. Harari-Steinberg, E. Buzhor, D. Omer, N. Pode-Shakked, H. Ben-Hur, R. Halperin, D. Schneider, and B. Dekel, *PLoS One*, **2009**, *4*, e6709.
59. H. Ventura-Bixenshpaner, H. Asraf, M. Chakraborty, M. Elkabets, I. Sekler, K. M. Taylor, and M. Hershfinkel, *Scientific Reports*, **2018**, *8*, 8119.
60. V. K. Vincent-Chong, A. Anwar, L. P. Karen-Ng, S. C. Cheong, Y.-H. Yang, P. J. Pradeep, Z. A. A. Rahman, S. M. Ismail, Z. M. Zaini, N. Prepageran, T. G. Kallarakkal, A. Ramanathan, N. A. B. M. Mohayadi, N. S. B. M. Rosli, W. M. W. Mustafa, M. T. Abraham, K. K. Tay, and R. B. Zain, *PLoS One*, **2013**, *8*.
61. L. Sim, C. Willemsma, S. Mohan, H. Y. Naim, B. M. Pinto, and D. R. Rose, *J Biol Chem*, **2010**, *285*, 17763.
62. M. G. Vander Heiden, *Nat Rev Drug Discov*, **2011**, *10*, 671.
63. C. Bianco, M. C. Rangel, N. P. Castro, T. Nagaoka, K. Rollman, M. Gonzales, and D. S. Salomon, *Am J Pathol*, **2010**, *177*, 532.
64. L. Strizzi, C. Bianco, A. Raafat, W. Abdallah, C. Chang, D. Raafat, M. Hirota, S. Hamada, Y. Sun, N. Normanno, R. Callahan, L. Hinck, and D. Salomon, *Journal of Cell Science*, **2005**, *118*, 4633.
65. H. Na, X. Liu, X. Li, X. Zhang, Y. Wang, Z. Wang, M. Yuan, Y. Zhang, S. Ren, and Y. Zuo, *J Hematol Oncol*, **2017**, *10*, 28.
66. Y. Zhang, Q. Zhang, M. Zhang, M. Yuan, Z. Wang, J. Zhang, X. Zhou, Y. Zhang, F. Lin, H. Na, S. Ren, and Y. Zuo, *Mol Cancer*, **2017**, *16*, 78.
67. P. Linder, **2011**, 12.
68. G. M. Bol, M. Xie, and V. Raman, *Molecular Cancer*, **2015**, *14*, 188.
69. T.-C. Lin, *Int J Mol Sci*, **2019**, *21*.
70. C. N. Nguyen, K. V. A. Nguyen, S. Eom, Y.-J. Choi, L.-J. Kang, J. Lee, C. Kim, S. Lee, S.-G. Lee, and J.-H. Lee, *Phytomedicine*, **2019**, *65*, 153096.
71. T. Abbas and A. Dutta, *Nat Rev Cancer*, **2009**, *9*, 400.
72. B. Klucky, R. Mueller, I. Vogt, S. Teurich, B. Hartenstein, K. Breuhahn, C. Flechtenmacher, P. Angel, and J. Hess, *Cancer Res*, **2007**, *67*, 8198.
73. H. Chen, E. Sells, R. Pandey, E. R. Abril, C.-H. Hsu, R. S. Krouse, R. B. Nagle, G. Pampalakis, G. Sotiropoulou, and N. A. Ignatenko, *Oncotarget*, **2019**, *10*, 6062.

74. D. Liberg, M. Sigvardsson, and P. Åkerblad, *Mol Cell Biol*, **2002**, *22*, 8389.
75. E. J. Rodger, A. Chatterjee, P. A. Stockwell, and M. R. Eccles, *Clinical Epigenetics*, **2019**, *11*, 114.
76. F. Zhao, R. McCarrick-Walmsley, P. Akerblad, M. Sigvardsson, and T. Kadesch, *Mol Cell Biol*, **2003**, *23*, 3837.
77. L. Gómez-H, N. Felipe-Medina, M. Sánchez-Martín, O. R. Davies, I. Ramos, I. García-Tuñón, D. G. de Rooij, I. Dereli, A. Tóth, J. L. Barbero, R. Benavente, E. Llano, and A. M. Pendas, *Nature Communications*, **2016**, *7*, 13298.
78. A. Taguchi, A. D. Taylor, J. Rodriguez, M. Celiktaş, H. Liu, X. Ma, Q. Zhang, C.-H. Wong, A. Chin, L. Girard, C. Behrens, W. L. Lam, S. Lam, J. D. Minna, I. I. Wistuba, A. F. Gazdar, and S. M. Hanash, *Cancer Res*, **2014**, *74*, 4694.
79. X. Wu, D. Lv, M. Lei, C. Cai, Z. Zhao, M. Eftekhar, D. Gu, and Y. Liu, *Oncol Lett*, **2020**, *20*, 2906.
80. P.-A. Hueber, R. Fukuzawa, R. Elkares, L. Chu, M. Blumentkrantz, S.-J. He, M. R. Anaka, A. E. Reeve, M. Eccles, N. Jabado, D. M. Iglesias, and P. R. Goodyer, *Pediatr Dev Pathol*, **2009**, *12*, 347.
81. H.-B. Qiu, K. Yang, H.-Y. Yu, and M. Liu, *Eur Rev Med Pharmacol Sci*, **2019**, *23*, 8391.
82. Q.-Q. Zhu, C. Ma, Q. Wang, Y. Song, and T. Lv, *Tumour Biol*, **2016**, *37*, 185.
83. J. Meng, S. Chen, J.-X. Han, B. Qian, X.-R. Wang, W.-L. Zhong, Y. Qin, H. Zhang, W.-F. Gao, Y.-Y. Lei, W. Yang, L. Yang, C. Zhang, H.-J. Liu, Y.-R. Liu, H.-G. Zhou, T. Sun, and C. Yang, *Cancer Res*, **2018**, *78*, 4150.
84. N. P. Mastroyiannopoulos, A. A. Antoniou, A. Koutsoulidou, J. B. Uney, and L. A. Phylactou, *Biosci Rep*, **2013**, *33*.
85. K. Machida, *Antioxid Redox Signal*, **2018**, *28*, 1080.
86. K. Misawa, D. Mochizuki, A. Imai, Y. Misawa, S. Endo, M. Mima, H. Kawasaki, T. E. Carey, and T. Kanazawa, *Clinical Epigenetics*, **2017**, *9*, 64.
87. K. Misawa, Y. Misawa, A. Imai, D. Mochizuki, S. Endo, M. Mima, R. Ishikawa, H. Kawasaki, T. Yamatodani, and T. Kanazawa, *J Cancer*, **2018**, *9*, 941.
88. Y. Shikauchi, A. Saiura, T. Kubo, Y. Niwa, J. Yamamoto, Y. Murase, and H. Yoshikawa, *Mol Cell Biol*, **2009**, *29*, 1944.
89. Y. Xu, X. Lin, J. Xu, H. Jing, Y. Qin, and Y. Li, *J Cancer*, **2018**, *9*, 1078.
90. S. Schiaffino, A. C. Rossi, V. Smerdu, L. A. Leinwand, and C. Reggiani, *Skeletal Muscle*, **2015**, *5*, 22.
91. B. Ferrán, I. Martí-Pàmies, J. Alonso, R. Rodríguez-Calvo, S. Aguiló, F. Vidal, C. Rodríguez, and J. Martínez-González, *Scientific Reports*, **2016**, *6*, 25944.
92. U. Seidel and H. H. Arnold, *J Biol Chem*, **1989**, *264*, 16109.

Supplementary Figures

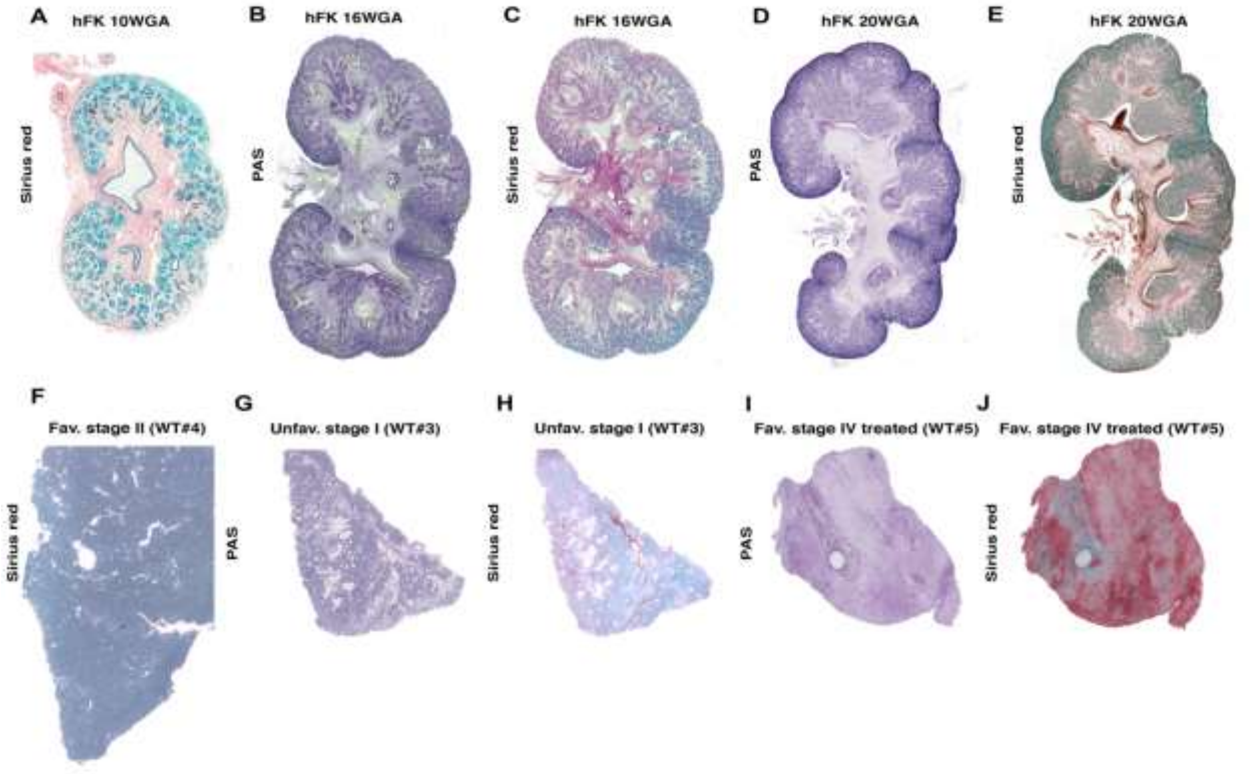

Supplementary Figure 1: Comparative immunohistochemistry: hFK across gestational ages and different WT subtypes.

A. Sirius red staining for collagen (red) fibers and cytoplasm (green) in hFK 10 WGA. **B-E**. Periodic Acid Schiff staining (PAS, glycogen, purple; **B, D**) and Sirius red staining (**C, E**) of hFK at 16 WGA and 20 WGA reveals increased complexity, organization, and collagen fiber deposition as hFK renal structures mature, including larger glomeruli, distinguishable renal compartments (medulla and cortex), and the renal pyramids. **F.** Sirius red staining visualizing collagen fibers (red) and cytoplasm (green) in favorable stage II WT#4. **G-J.** PAS staining (**G, I**) and Sirius red staining (**H, J**) of unfavorable stage I WT#3 and favorable chemo-treated, stage IV WT#5 showing wide structural heterogeneity between WT subtypes. Unfavorable WT#3 expresses more collagen fibers and contains tubule-like structures, while favorable WT#4 has multiple blastema foci. WT#5 (chemotherapy-treated favorable stage IV) appears fibrotic with only patches of cell clusters.

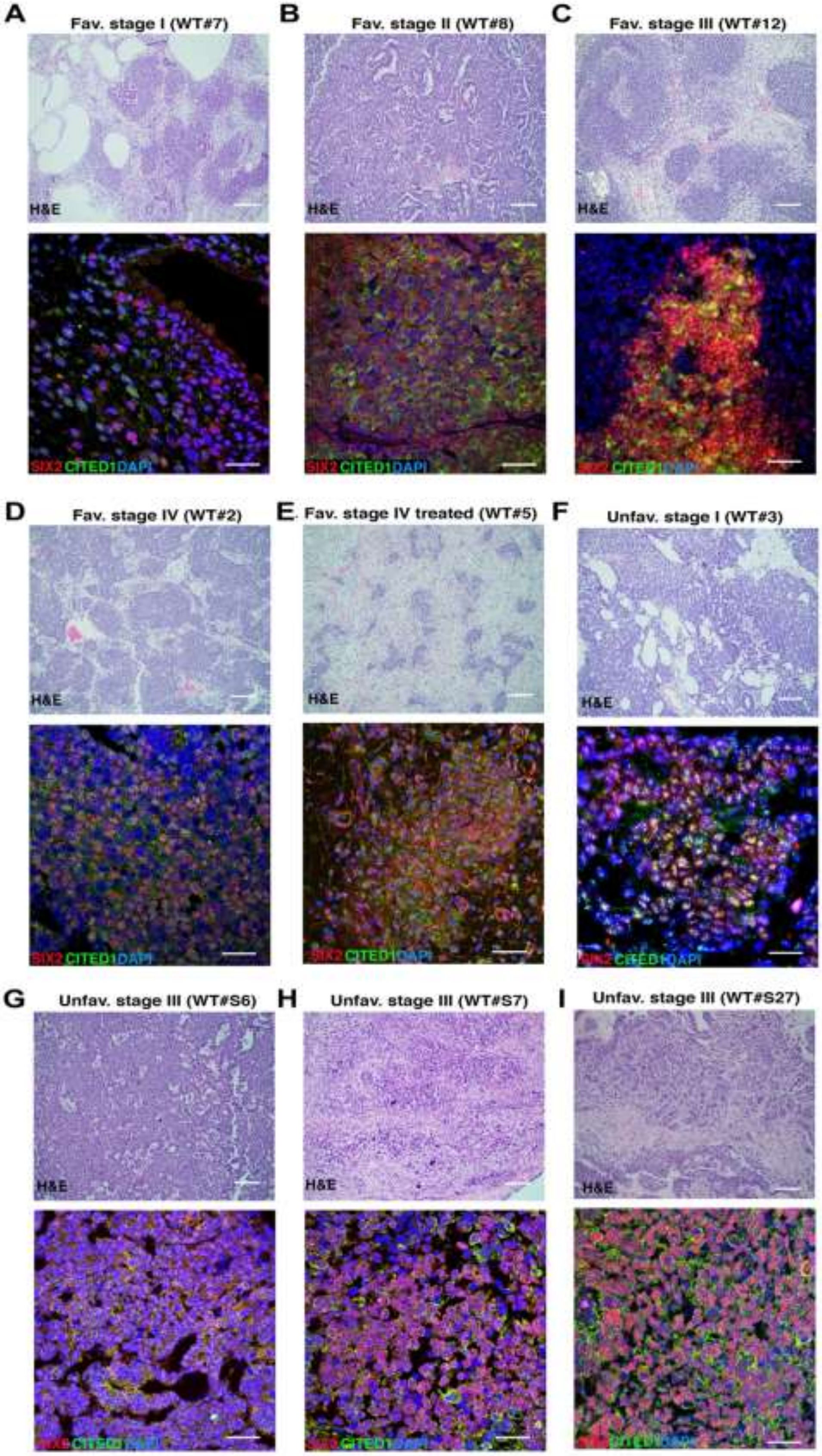

A
Fav. stage I (WT#7)
B
Fav. stage II (WT#8)
C
Fav. stage III (WT#12)
D
Fav. stage IV (WT#2)
E
Fav. stage IV treated (WT#5)
F
Unfav. stage I (WT#3)
G
Unfav. stage III (WT#S6)
H
Unfav. stage III (WT#S7)
I
Unfav. stage III (WT#S27)
H&E
SIX2 CITED1 DAPI

Supplementary Figure 2: Immunohistochemistry and identification of SIX2+CITED1+ cells in WT subtypes.

A-I. Top panel: H&E staining. Bottom panel: SIX2 (red) and CITED1 (green) immunofluorescence staining of WT favorable stage I (**A**, WT#7), stage II (**B**, WT#8), stage III (**C**, WT#12), and stage IV (**D**, WT#2), favorable chemotherapy-treated stage IV (**E**, WT#5), unfavorable stage I (**F**, WT#3), unfavorable stage III (**G**, WT#S6), unfavorable stage III (**H**, WT#S7), and unfavorable stage III (**I**, WT#S27). Nuclei stained with DAPI (blue). Scale bar = 50µm. Distribution and expression of SIX2 and CITED1 vary between different WT locations and subtypes.

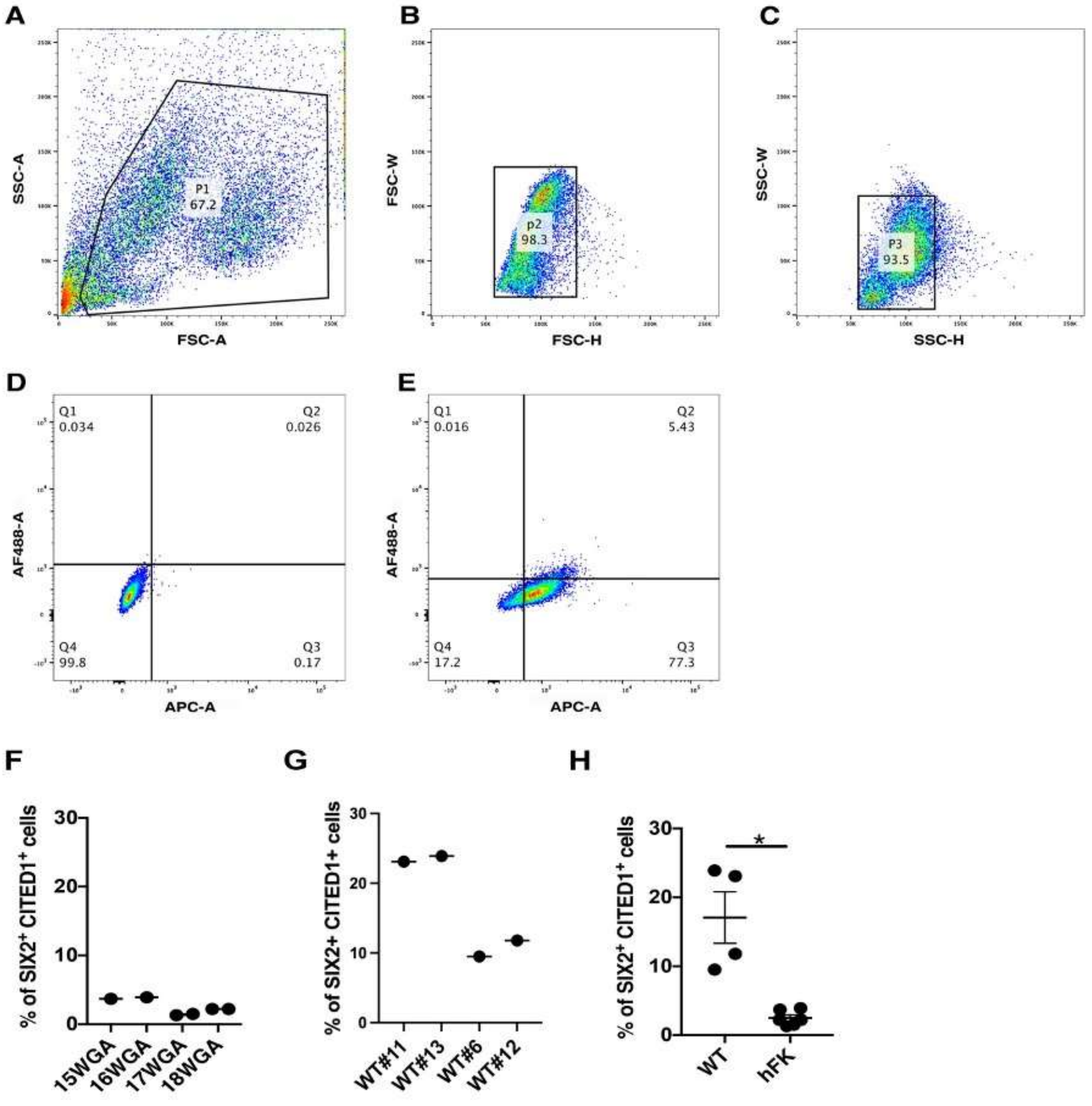

Supplementary Figure 3. Flow cytometry gating strategy and selection.

A-E. Representative flow cytometry dot-plots of a single cell suspension from a WT sample. Unstained and single-positive controls performed area scaling, excluded autofluorescence, and performed fluorochrome compensation when needed. Cells were first gated based on forward scatter (FSC-A), and side scatter (SSC-A) (**A**, P1) to exclude dead cells and cellular debris from the analysis. Further gating was performed to remove duplets based on FSC-H/FSC-W (**B,** P2) and SSC-H/SSC-W (**C,** P3). Quadrant gating was then drawn to exclude all events occurring in unstained cells for each channel (Alexa Fluor-488, APC, PE). The same gating strategy was used to analyze unstained samples (**D**) and stained samples (**E**). AF488 marks CITED1+ cells, APC marks SIX2+ cells. **F.** Flow cytometry analysis of the percentage of SIX2+CITED1+ cells in hFK (n=6, between 15-18 WGA). **G.** Flow cytometry analysis of the percentage of SIX2+CITED1+ in different WT subtypes: favorable stage II (WT#11 and #13), favorable stage III (WT#6 and #12). **H.** Flow cytometry comparison of SIX2+CITED1+ cells as a percentage of the total cell population in different WT and hFK samples. *p<0.05; means ± SEM.

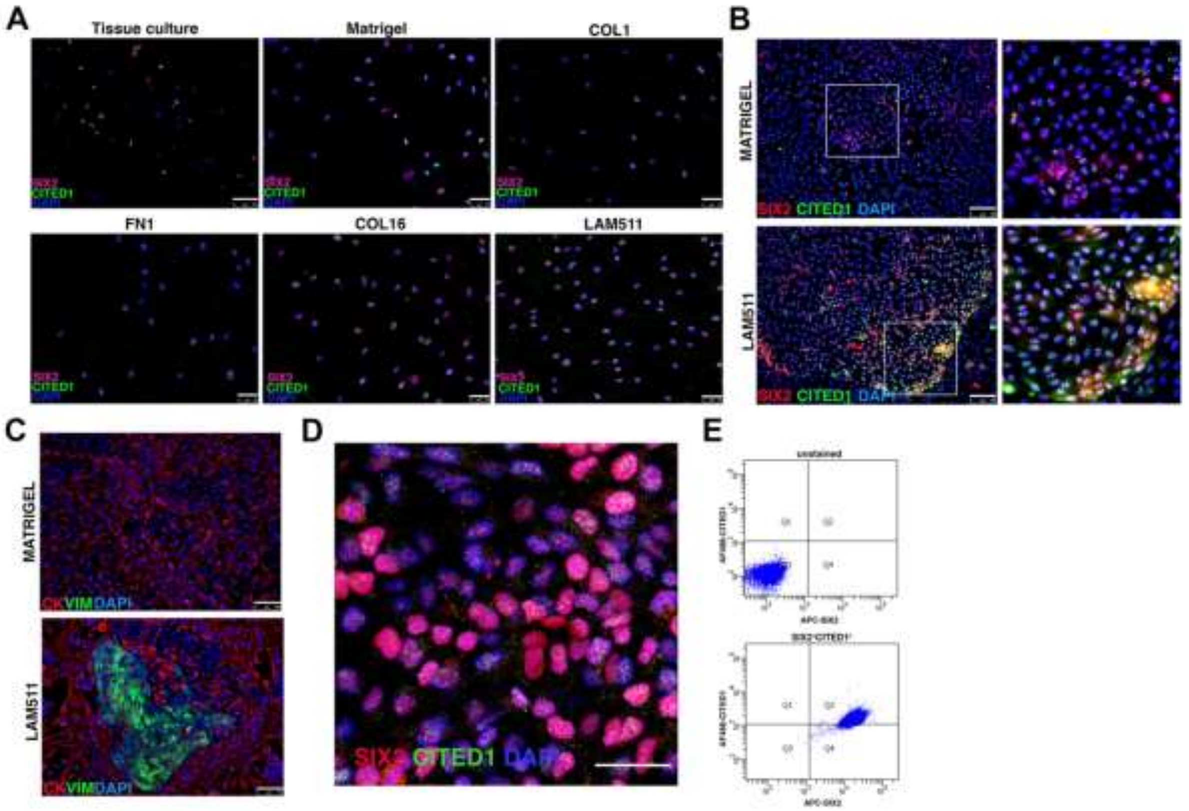

Supplementary Figure 4. Expression of SIX2 and CITED1 in hFK SIX2+CITED1+ cells cultured on different substrates and culture of WT SIX2+CITED1+ cells.

A. Representative immunostaining for CITED1 (green) and SIX2 (red) in SIX2+CITED1+ cells from hFK (17 WGA) cultured on plastic (no substrate), matrigel, COL1, fibronectin (FN1), COL16 or laminin511 for 5 days. Nuclei stained with DAPI (blue). Scale bar=25µm. **B**. Representative immunostaining for CITED1 (green) and SIX2 (red) in SIX2+CITED1+ cells from hFK (17 WGA) cultured on matrigel or laminin511 for 28 days. Nuclei stained with DAPI (blue). Scale bar=100 µm. High power images of the quadrant on the right panel. **C**. Representative immunostaining for cytokeratin (CK, red) and vimentin (VIM, green) in SIX2+CITED1+ cells from hFK (17 WGA) cultured on matrigel or laminin511 for 28 days. Nuclei stained with DAPI (blue). Scale bar=100 µm. LAM511 provides a surface that maintains SIX2+CITED1+ expression during culture expansion. **D.** Representative immunostaining for CITED1 (green) and SIX2 (red) of SIX2+CITED1+ cells from WT#8 (favorable stage II) after 6 passages in culture. Nuclei stained with DAPI (blue). Scale bar = 50 µm. **E.** Flow cytometry analysis of SIX2+CITED1+ cells as percentage from WT#8 (favorable Stage II) after 6 passages. Detection of SIX2 (APC) and CITED1 (AF488) expression shows that 92% of the cells are SIX2+ and 86% are SIX2+CITED1+. An unstained sample is also shown as a control (top panel).

A

Tumor Code	Cells Injected	Location	Condition	Cell numbers	Generation	Treatment	Xenografts/ Injection
WT#8	SIX2+CITED1+	Subcutaneous	Cultured p6-12	6.00E+06	1st	None	20/20
WT#8	SIX2+CITED1+	Subcutaneous	Cultured p6-12	6.00E+06	1st	anti-ITGB1	6/6
WT#8	SIX2+CITED1+	Subcutaneous	Cultured p6-12	6.00E+06	1st	anti-ITGB4	10/10
WT#8	SIX2+CITED1+	Subcutaneous	Cultured p6-12	6.00E+06	1st	Vincristine	4/4
WT#8	SIX2+CITED1+	Subcutaneous	Freshly Isolated	5.00E+05	1st	None	1/1
WT#8	SIX2+CITED1+	Subcutaneous	Freshly Isolated	2.00E+06	1st	None	1/1
WT#13	SIX2+CITED1+	Subcutaneous	Freshly Isolated	5.00E+05	1st	None	1/1
WT#13	SIX2+CITED1+	Subcutaneous	Freshly Isolated	2.00E+06	1st	None	1/1
WT#11	SIX2+CITED1+	Subcutaneous	Freshly Isolated	5.00E+05	1st	None	1/1
WT#8	SIX2+CITED1+	Intrarenal	Freshly Isolated	1.00E+03	1st	None	4/4
WT#8	SIX2+CITED1+	Intrarenal	Freshly Isolated	1.00E+04	1st	None	4/4

B

Tumor Code	Cells injected	Location	Condition	Cell numbers	Generation	Treatment	Xenografts/ Injection
WT#8	SIX2+CITED1+	Intrarenal	Freshly Isolated	1.00E+03	2nd	None	4/4
WT#8	SIX2+CITED1+	Intrarenal	Freshly Isolated	1.00E+04	2nd	None	4/4
WT#8	SIX2+CITED1+	Subcutaneous	Freshly Isolated	1.00E+04	2nd	None	1/4
WT#8	SIX2+CITED1+	Subcutaneous	Freshly Isolated	1.00E+03	3rd	None	1/4

Supplementary Figure 5. Table of xenotransplantation experiments.

A. Table of tumors generated using SIX2+CITED1+ cells isolated from a primary patient sample (1st generation). **B.** Table of tumors generated using SIX2+CITED1+ cells isolated from xenografts generated using SIX2+CITED1+ cells either from a primary patient (2nd generation) or another xenograft generated with SIX2+CITED1+ cells from a primary patient (3rd).

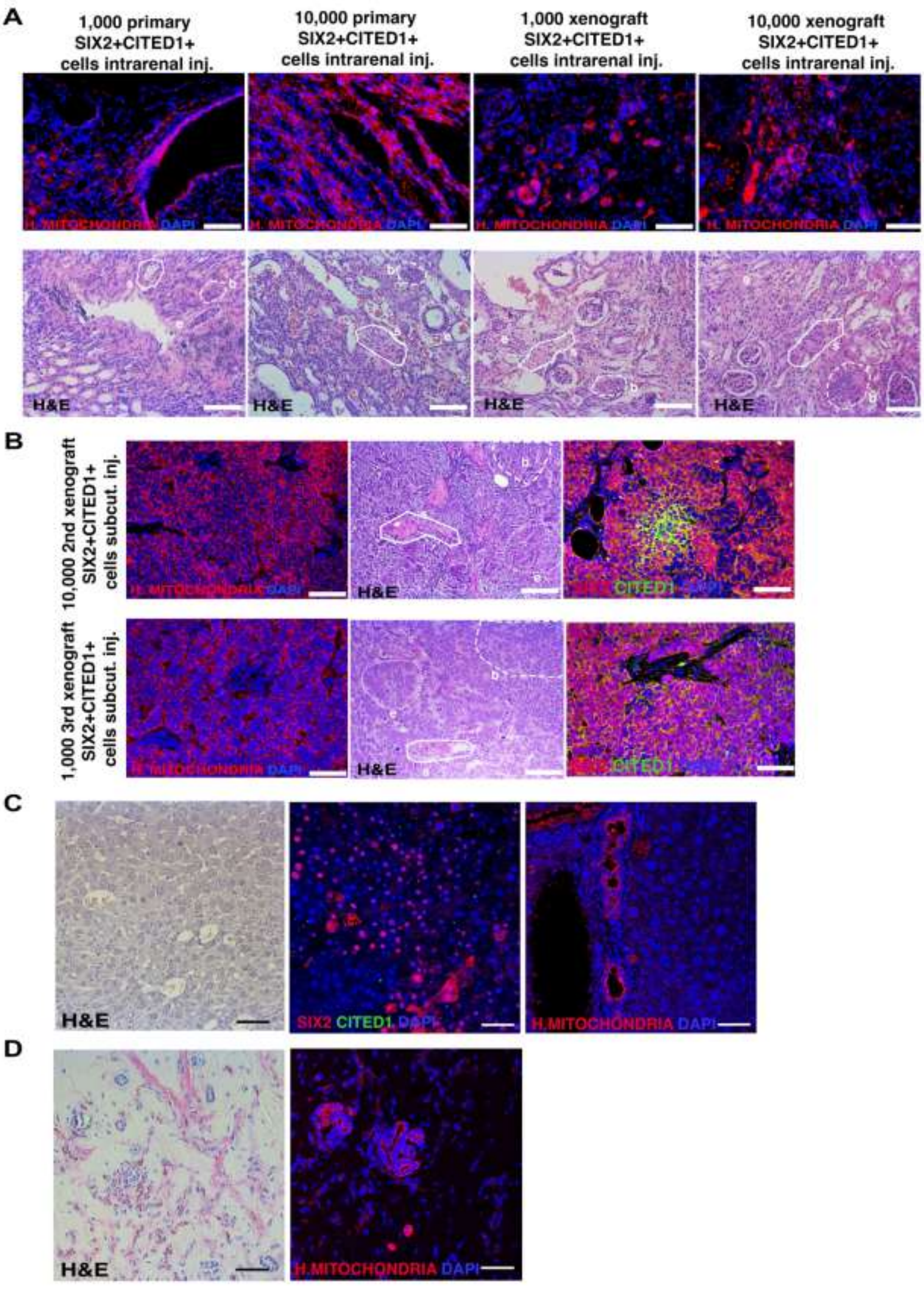

A
1,000 primary SIX2+CITED1+ cells intrarenal inj.
10,000 primary SIX2+CITED1+ cells intrarenal inj.
1,000 xenograft SIX2+CITED1+ cells intrarenal inj.
10,000 xenograft SIX2+CITED1+ cells intrarenal inj.
H. MITOCHONDRIA DAPI
H&E
B
10,000 2nd xenograft SIX2+CITED1+ cells subcut. inj.
1,000 3rd xenograft SIX2+CITED1+ cells subcut. inj.
H. MITOCHONDRIA DAPI
H&E
SIX2 CITED1 DAPI
C
H&E
SIX2 CITED1 DAPI
H.MITOCHONDRIA DAPI
D
H&E
H.MITOCHONDRIA DAPI

Supplementary Figure 6. SIX2+CITED1+ cells in culture before transplantation, transcriptomic expression of drug resistance markers, and xenograft histology.

A. Representative immunofluorescence staining of human mitochondria (red, top panel) and H&E staining (bottom panel) of intrarenal injections from freshly isolated SIX2+CITED1+ from WT#8 (favorable stage II) and from freshly isolated SIX2+CITED1+ cells from xenografts generated with WT#8 (favorable stage II) SIX2+CITED1+ cells at different dilutions 1,000 and 10,000. Nuclei stained with DAPI (blue); Scale bar = 50 µm. **B.** Representative immunofluorescence staining of human mitochondria (red) immunofluorescence staining, a representative H&E staining, and SIX2 (red) and CITED1 (green) immunofluorescence staining of subcutaneous injections of freshly isolated SIX2+CITED1+ from 2nd generation and 3rd generation xenografts generated with WT#8 (favorable stage II) SIX2+CITED1+ cells at a dilutions 10,000 and 1,000 cells respectively. **C.** Representative H&E staining, SIX2 (red) and CITED1 (green) and human mitochondria (red) immunofluorescence staining of mouse liver, showing metastasis of WT SIX2+CITED1+ xenograft. The metastasis expressed SIX2, but CITED1 was not detectable. Nuclei are stained in DAPI (blue). Scale bar = 50 µm. **D.** Representative H&E staining and human mitochondria (red) immunofluorescence staining of the xenograft generated from freshly isolated hFK SIX2+CITED1+ cells (17.4 WGA). Nuclei are stained in DAPI (blue). Scale bar = 50 µm.

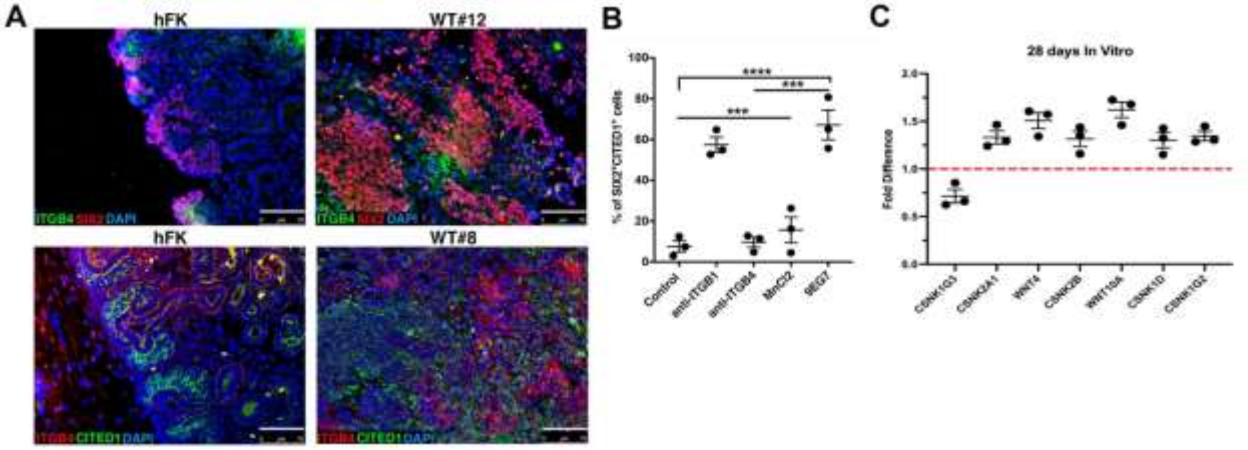

Supplementary Figure 7. Expression of ITGβ1 and ITGβ4 in hFK and WT samples and WNT signaling studies in vitro.

A. Representative immunofluorescence staining showing the distribution of ITGβ4 (green) and SIX2 (red) in hFK (10 WGA, left upper panel) and WT (WT#12: favorable stage III, right upper panel) and for ITGβ4 (red) and CITED1 (green) in hFK (10 WGA, left lower panel) and WT (WT#8: favorable stage II, right lower panel). Nuclei stained with DAPI (blue). Scale bar=75 µm.

B. Percentage of SIX2+CITED1+ cells from hFK (17.4 WGA) detected by flow cytometry after 28 days of culture without treatment (n=3, CTRL) or with either anti-ITGβ1 (n=3, ITGβ1), anti-ITGβ4 (n=3, ITGβ1), manganese (II) chloride for manganese-induced integrin affinity ($MnCl_{2,}$ n=3) or neutralizing antibody against the active form of ITGβ1 (9EG7, n=3). ***p<0.001, ****p<0.0001; means ± SEM. **C.** RT2 PCR Array of the WNT Signaling pathway on RNA extracted from 28-day in vitro experiment shows significantly changed genes in hFK SIX2+CITED1+ cells treated with anti-ITGβ4 (n=3) versus CTRL (n=3): *CSNK1G3, CSNK2A1, WNT4, CSNK2B, WNT10A, CSNK1D, and CSNK1G2*. p<0.05.

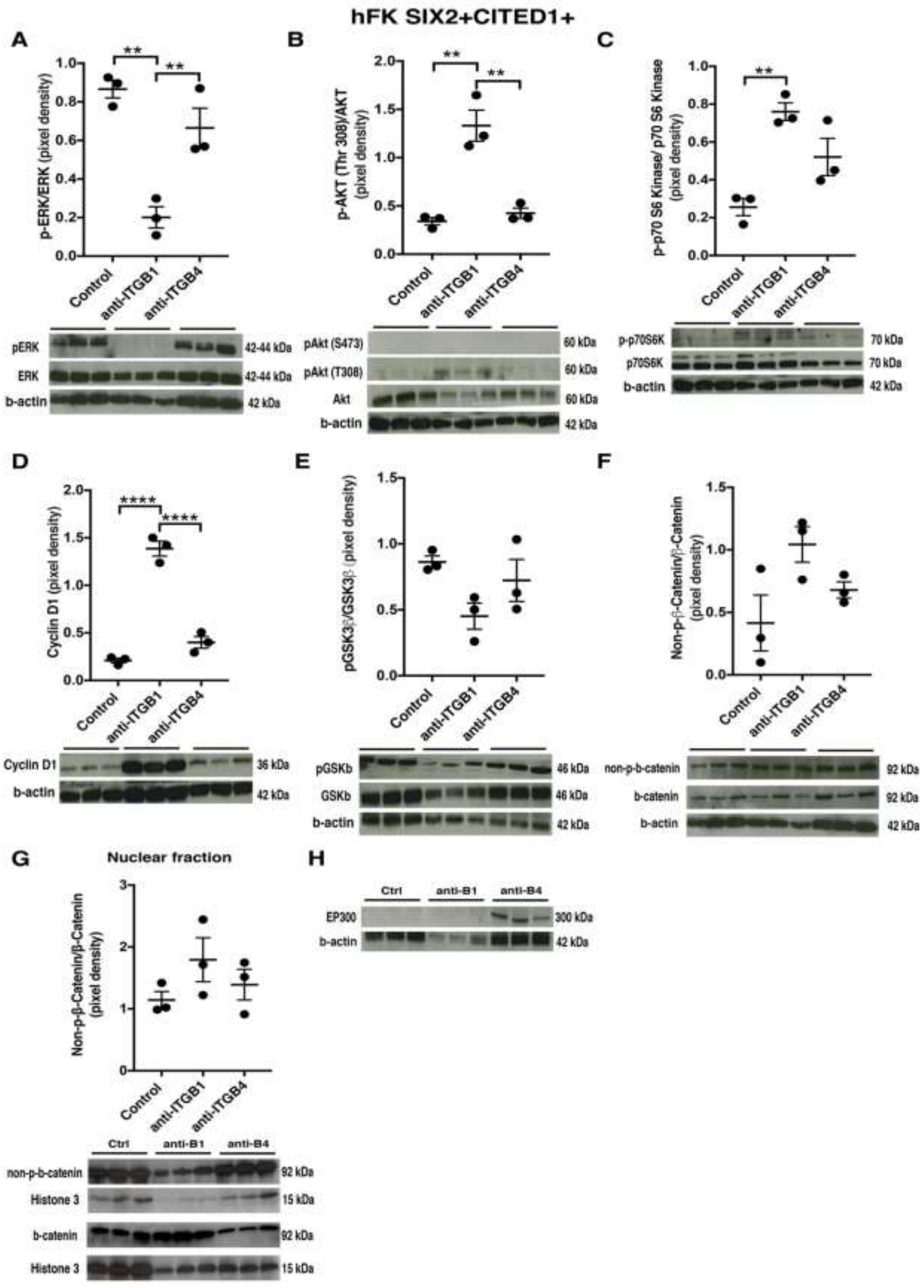
hFK SIX2+CITED1+
A
p-ERK/ERK (pixel density)
Control
anti-ITGB1
anti-ITGB4
pERK
ERK
b-actin
42-44 kDa
42 kDa
B
p-AKT (Thr 308)/AKT (pixel density)
pAkt (S473)
pAkt (T308)
Akt
60 kDa
C
p-p70 S6 Kinase/ p70 S6 Kinase (pixel density)
p-p70S6K
p70S6K
70 kDa
D
Cyclin D1 (pixel density)
Cyclin D1
36 kDa
E
pGSK3β/GSK3β (pixel density)
pGSKb
GSKb
46 kDa
F
Non-p-β-Catenin/β-Catenin (pixel density)
non-p-b-catenin
b-catenin
92 kDa
G
Nuclear fraction
Ctrl
anti-B1
anti-B4
Histone 3
15 kDa
H
EP300
300 kDa

Supplementary Figure 8. ITGβ1 and ITGβ4 control cell signaling in SIX2+CITED1+ cells in vitro.

A-H. Densitometric analysis of pERK/ERK ratio (**A**), pAKT(S473)/AKT and pAKT(T308)/AKT ratio (**B**), p-p706SK/p70S6K ratio (**C**), cyclin D1 (**D**), pGSKβ/GSKβ ratio (**E**), non-phosphorylated β-catenin and β-catenin, total fraction (**F**), non-phosphorylated β-catenin and β-catenin nuclear fraction (**G**), and EP300 (**H**, note: EP300 was not detected in untreated SIX2+CITED1+ cells or cells cultured with anti-ITGβ1) after 28 days of culture in cells cultured without anti-integrin treatment (n=3, CTRL) and with either anti-ITGβ1 (n=3) or anti-ITGβ4 (n=3). β-actin was used as housekeeping protein control for a-h, and histone 3 for the nuclear fraction control in g. WB bands are presented below the graph. *p<0.05; **p<0.01, ****p<0.0001; mean ± SEM.

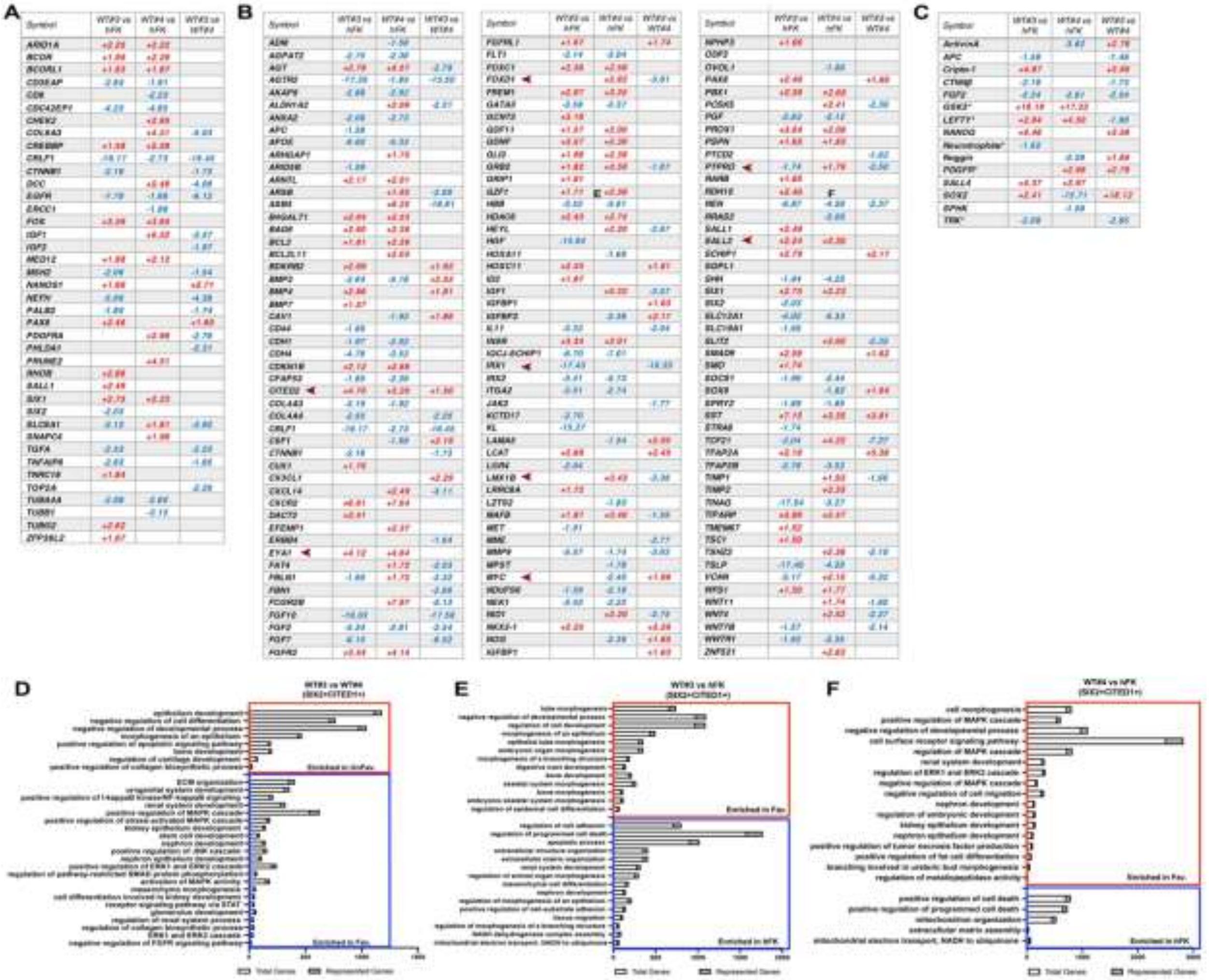

Supplementary Figure 9. Ingenuity Pathways Analysis and Gene Ontology (GO) analysis.

A. Ingenuity Pathway Analysis (IPA) of Wilms Tumor specific genes in SIX2+CITED1+ cells derived from WT#3 (unfavorable stage I), hFK (17, 17.2, and 17.5 WGA) and WT#4 (favorable stage III). Table of significantly upregulated and downregulated genes IPA of WT related diseased genes. **B.** IPA of nephrogenic development specific genes in SIX2+CITED1+ cells derived from WT#3 (unfavorable stage I), hFK (17, 17.2, and 17.5 WGA) and WT#4 (favorable stage III). Table shows significantly upregulated and downregulated genes in the nephrogenic development pathway. Genes of interest are marked with red arrows. **C.** IPA of WT pluripotency specific genes in SIX2+CITED1+ cells derived from WT#3 (unfavorable stage I), hFK (17, 17.2, and 17.5 WGA) and WT#4 (favorable stage III). Table of significantly upregulated and downregulated genes. **D-F.** GO comparisons of biological pathways (up-regulated in red boxes; down-regulated in blue boxes) in **D**. WT#3 (unfavorable stage I) vs WT#4 (favorable stage III); The bar graph shows GO sets enriched in WT#3 SIX2+CITED1+ (red quadrant) or enriched in WT#4 SIX2+CITED1+ (blue quadrant). **E**. WT#3 vs hFK (averaged RPKM from 17, 17.2, and 17.5 WGA); **F**. WT#4 vs hFK (averaged RPKM from 17, 17.2, and 17.5 WGA).

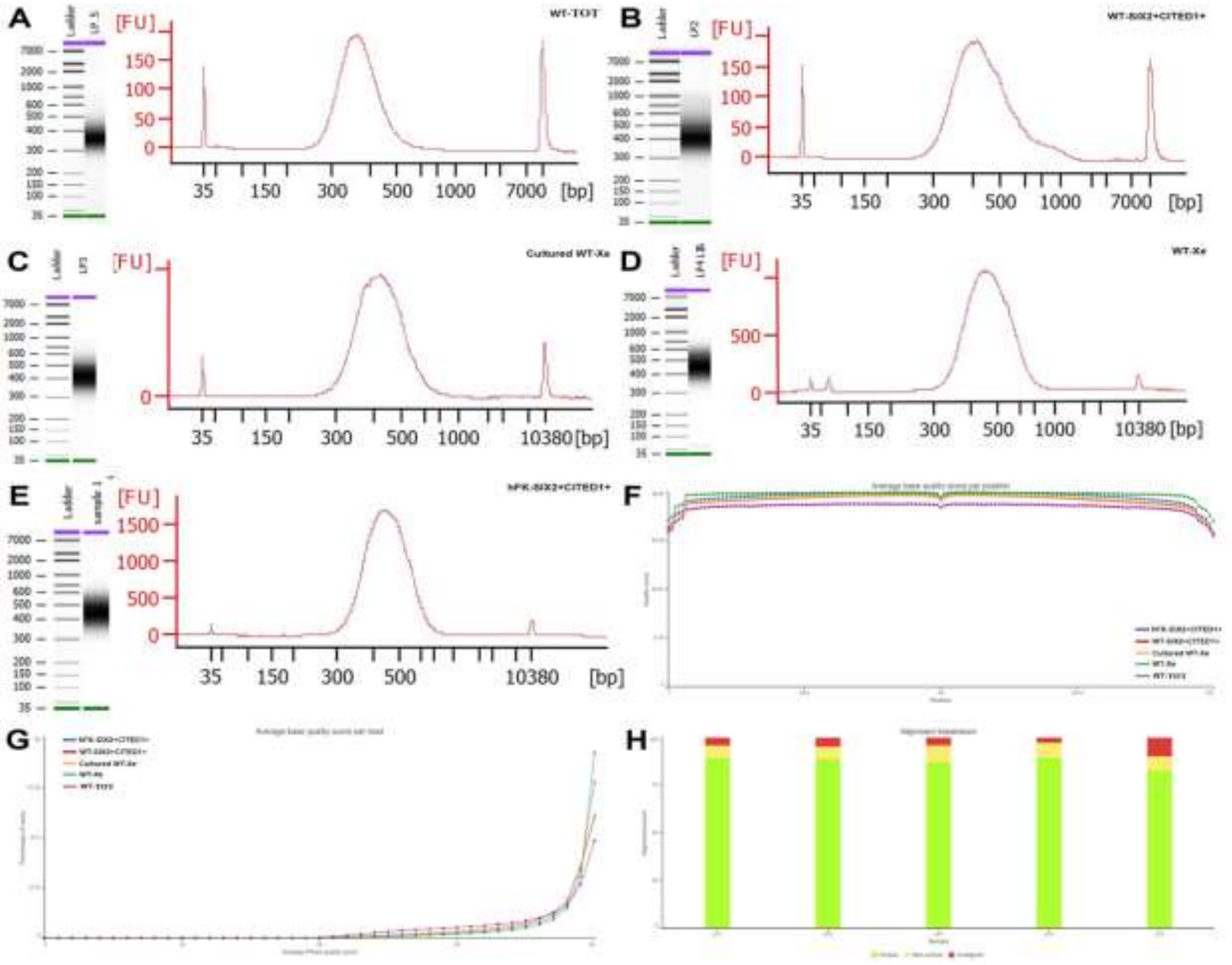

Supplementary Figure 10. scRNA-seq: RNA quality and read alignment.

A-E. Images showing RNA quality control for the different samples for scRNA-seq analysis (**A**); total digested WT cells (WT-TOT) (**B**); WT SIX2+CITED1+ cells, **C**); Xenografts generated with in vitro expanded WT SIX2+CITED1+ cells (WT-Xe cultured) **D**); Xenografts generated with freshly isolated WT SIX2+CITED1+ cells (WT-Xe fresh isolated) **E**); and hFK SIX2+CITED1+ cells show a distinct cDNA peak just below 1,000 bp in all samples, with no amplification in a negative water control in column F2. **F-G.** RNA-Seq QA/QC of Phred quality scores shows an average score >30 in all base positions, and a Phred score >30 for the vast majority of reads. **H.** The percentage of total alignments (green and yellow) was ~90% per sample, with ~80% of all reads aligned uniquely (green).

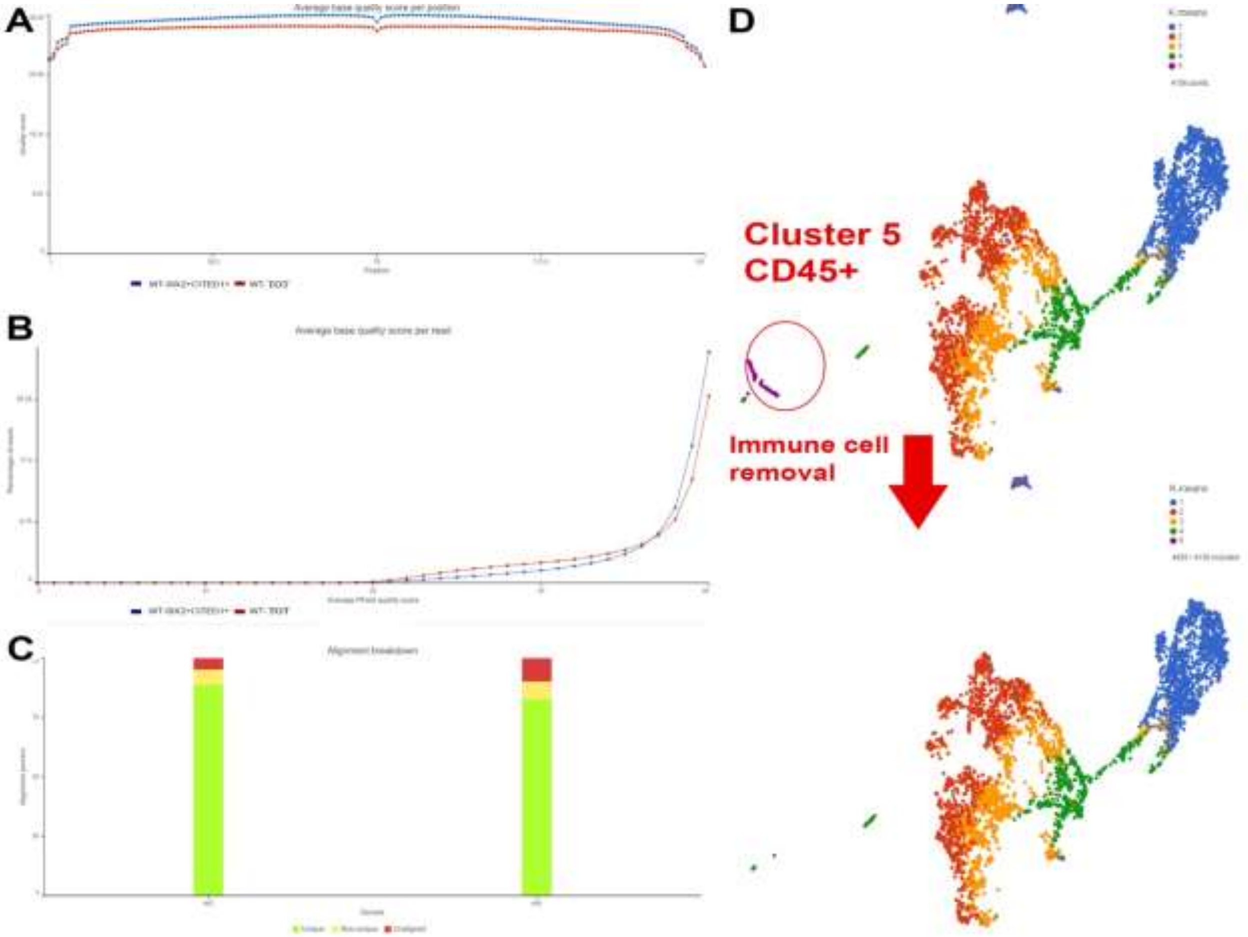

Supplementary Figure 11. scRNA-seq: removal of immune cells from dataset and Principal component analysis (PCA).

A-B. RNA-Seq QA/QC of Phred quality scores shows an average score >30 in all base positions, and a Phred score >30 for the vast majority of reads. **C.** The percentage of total alignments (green and yellow) was ~90% per sample, with ~80% of all reads aligned uniquely (green) **D.** Following k-means clustering (n=5), the cluster expressing the immune marker CD45 (195 cells, cluster 5) was filtered out to avoid confounding results, and the remaining cells were processed by graph-based clustering as described in Figure 3.

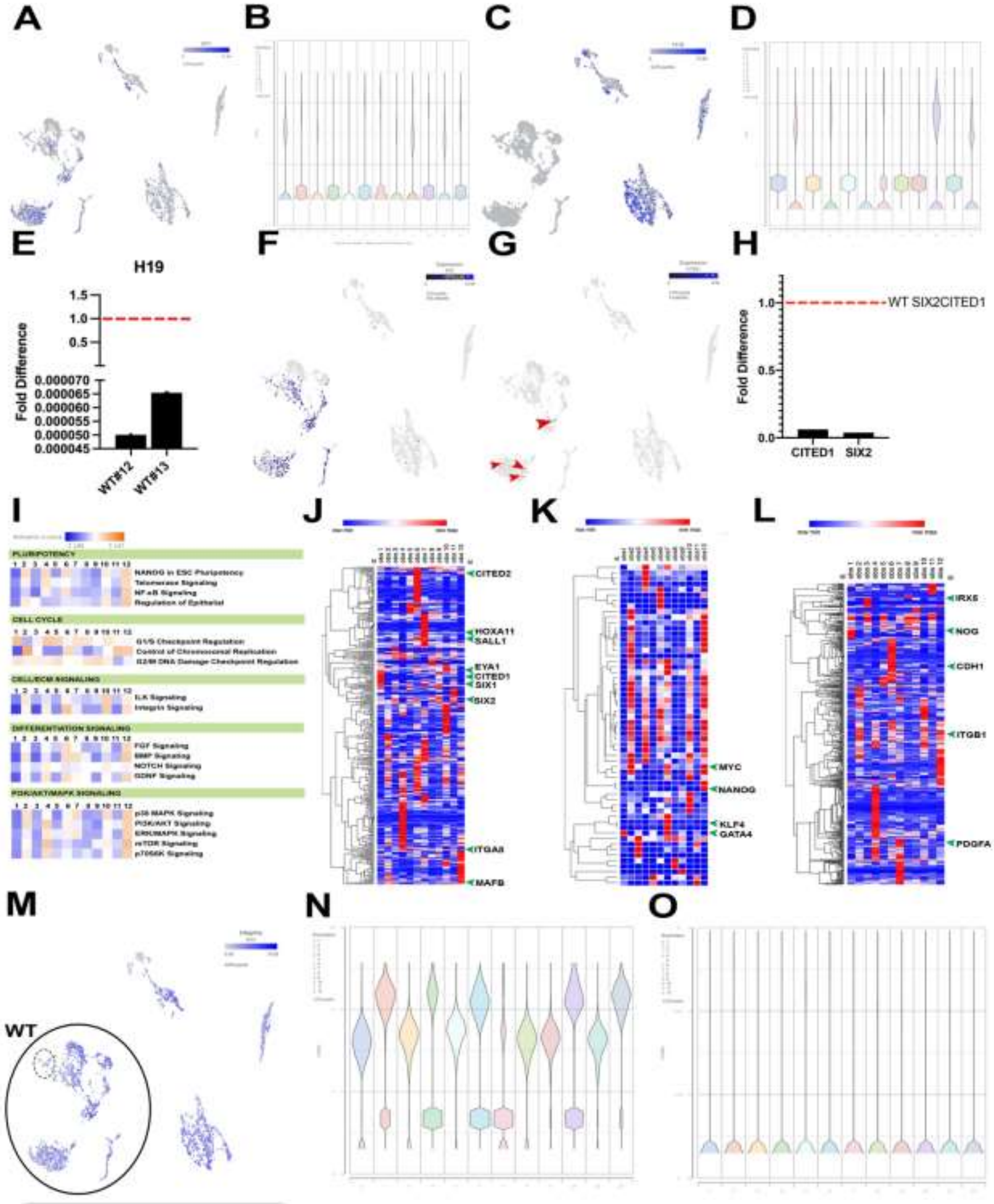

Supplementary Figure 12. Transcriptional profiling of hFK and WT SIX2+CITED1+ cells.

A. Uniform Manifold Approximation and Projection (UMAP) embedding colored for WT1 expression. WT SIX2+CITED1+ cell clusters markedly overexpress WT1 compared to hFK SIX2+CITED1+ cell clusters. **B.** Violin plots showing expression of WT1 across the clusters. **C.**

UMAP embedding colored for H19 expression. WT SIX2+CITED1+ cell clusters are completely devoid of H19 compared to the hFK SIX2+CITED1+ cell clusters. **D.** Violin plots showing the expression of H19 across the clusters. **E.** qPCR analysis showing fold differences of H19 expression in WT SIX2+CITED1+ cells (WT#12 favorable stage III and WT#13favorable stage II) relative to hFK SIX2+CITED1+ cells (defined as 1-fold) showing marked reduction in WT cells. **F-G.** UMAP embedding colored for expression of *SIX2* (**F**) and *CITED1* (**G**, red arrows) identifying cells expressing *SIX2 or/and CITED1*. **H.** qPCR analysis showing fold differences of hFK SIX2+CITED1+ cells for expression of SIX2+ and CITED1+ relative to WT SIX2+CITED1+ cell expression (defined as 1-fold). **I**. Ingenuity Pathway Analysis (IPA) identified canonical pathways for pluripotency, cell cycle, cell-ECM signaling, differentiation, and PI3K/Akt/MAPK pathways as most significantly associated with DE genes between different clusters. **J-L**. Heatmaps showing gene expression profiles of all clusters relative to nephrogenesis (**J**), pluripotency and self-renewal (**K**) and renal commitment, specification, differentiation (**L**). Genes of interest are highlighted by green arrowheads. Full list of genes found in SupplementaryDataset#3. **M.** UMAP embedding colored for expression of integrin α and β chains in SIX2+CITED1+ cells from hFK and WT. **N.** Violin plots showing the expression of ITGβ1 across clusters in SIX2+CITED1+ cells from hFK and WT. **O.** Violin plots showing the expression of ITGβ4 across the clusters in SIX2+CITED1+ cells from hFK and WT.

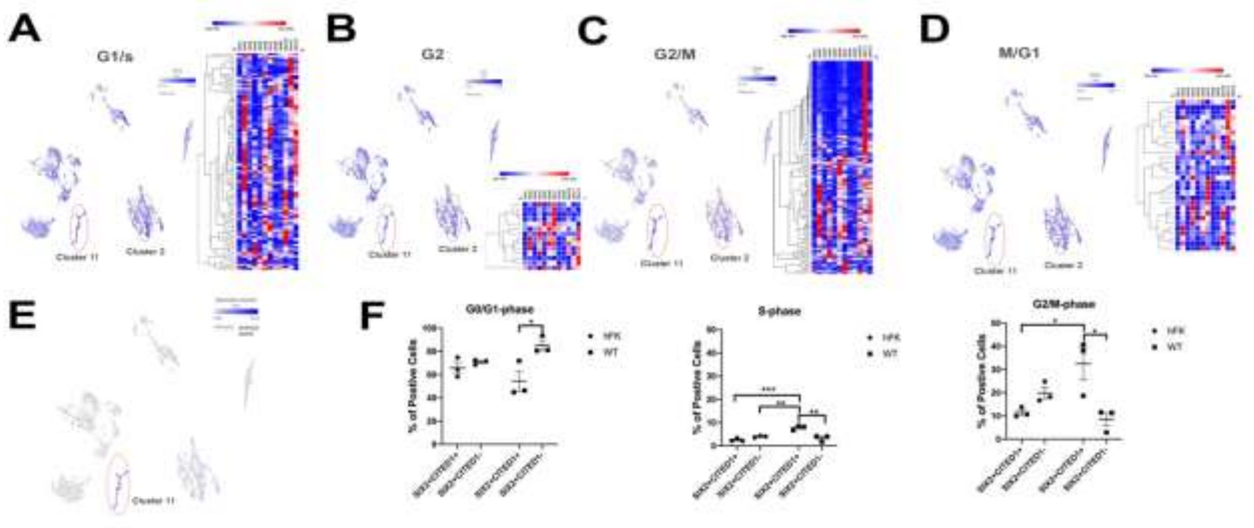

Supplementary Figure 13. Cell cycle in hFK and WT.

A-D. Uniform Manifold Approximation and Projection (UMAP) embedding spot-colored for genes playing a role during G1/S (**A**), G2 (**B**), G2/M (**C**), and M/G1 (**D**) phases of the cell cycle and heatmaps depicting the same genes, showing expression across clusters. Cluster 11 exhibits

marked overexpression of G2/M phase genes compared to other clusters. **E.** UMAP embedding colored for expression of genes involved in symmetric division. WT SIX2+CITED1+ cell cluster 11 exhibits marked overexpression of genes involved in symmetric (*ASPM* and *SAPCD2*) division compared to the other WT SIX2+CITED1+ and hFK SIX2+CITED1+ cell clusters. **F**. Graphs showing the % of SIX2+CITED1+ and SIX2+CITED1- cells from hFK (n=3, 17.4, 17.5, and 18.1 WGA) and WT (n=3, favorable stage II, n=2; favorable stage III, n=1) at G1/G0, S, and G2/M phases of the cell cycle. *p<0.05; **p<0.01, ****p<0.001; mean ± SEM.

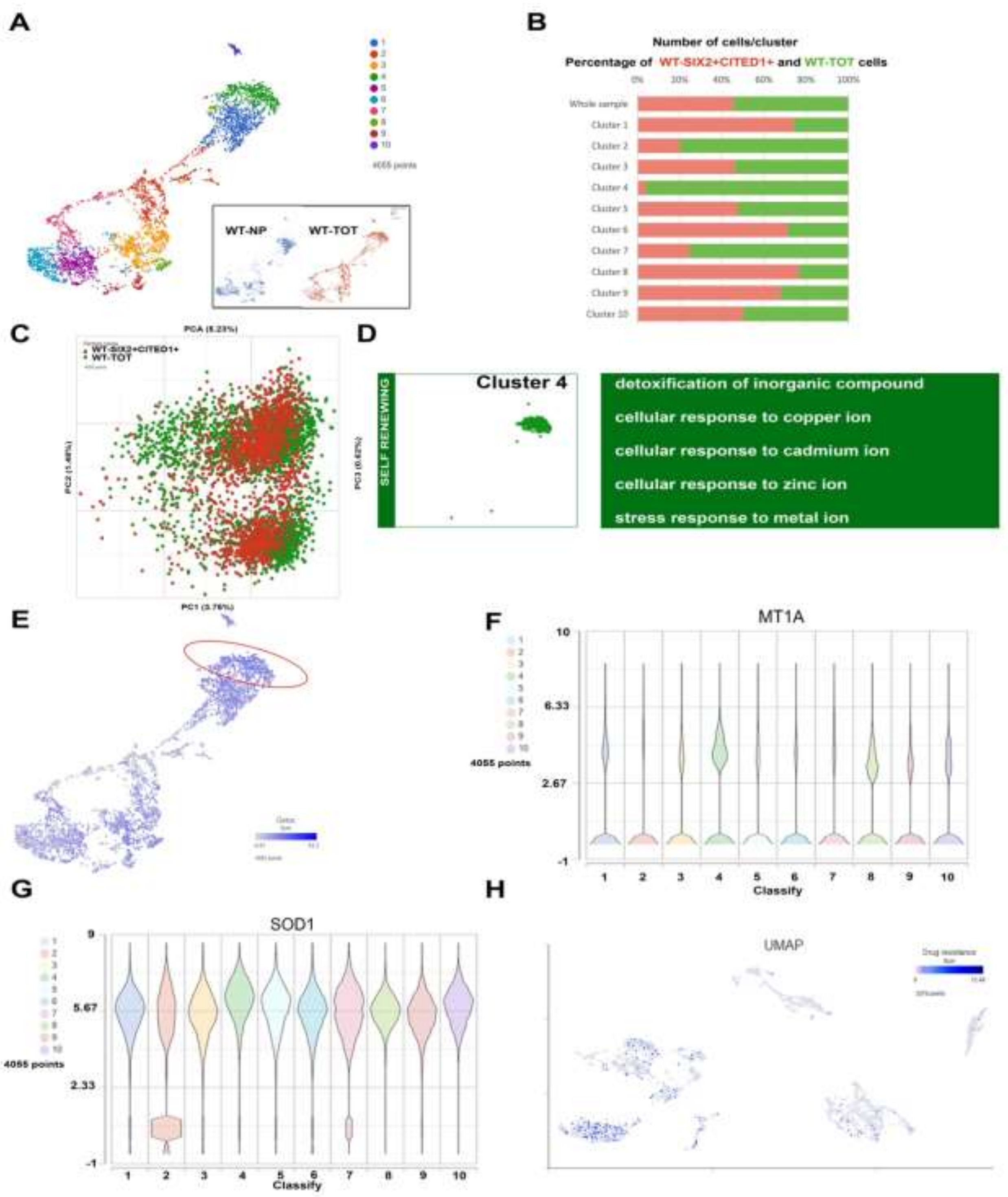

Supplementary Figure 14. Principal component (PCA) and integration analysis of WT SIX2+CITED1+ and WT-TOT cells.

A. Uniform Manifold Approximation and Projection (UMAP) of 4,055 droplet-based scRNA-seq profiles from the integration of SIX2+CITED1+ cells from WT#8 (favorable stage II) and WT of

origin total population (WT-TOT), colored by clusters generated by unsupervised assignment. Lower right smaller panel: UMAP colored by the sample of origin (SIX2+CITED1+ cells from WT in blue; WT-TOT in red). **B**. Fraction of cells (% of cells; *x*-axis; SIX2+CITED1+ cells from WT, red; WT-TOT, green) in each cluster (*y*-axis). **C**. PCA displaying results of expression-level data from WT SIX2+CITED1+ cells (red dots) and WT-TOT cells (green dots) along PC1 and PC2, which describe 3.75% and 1.48% of the variability, respectively, within the data set. **D**. Gene Ontology (GO) analysis for cluster 4 showed enrichment of gene sets related to detoxification of inorganic compounds including copper, cadmium, and zinc. P<0.05. Upregulated DE genes were used for the comparison. **E**. UMAP embedding colored for genes involved in detoxifying inorganic compounds confirms enrichment in cluster 4 (circled in red, grey: lower expression, dark blue, higher expression). **F-G**. Violin plots showing expression of metallothionine (*MT1X*, **F**) and superoxide dismutase 1 (*SOD1*, **G**) across clusters. **H.** UMAP embedding colored for expression of genes involved in drug resistance (*ABCB1, ABCG2, ABCB5, ABCC1, MDR3, MRP1, LRP*). WT SIX2+CITED1+ clusters exhibit marked overexpression of drug resistance genes compared to hFK SIX2+CITED1+ clusters.

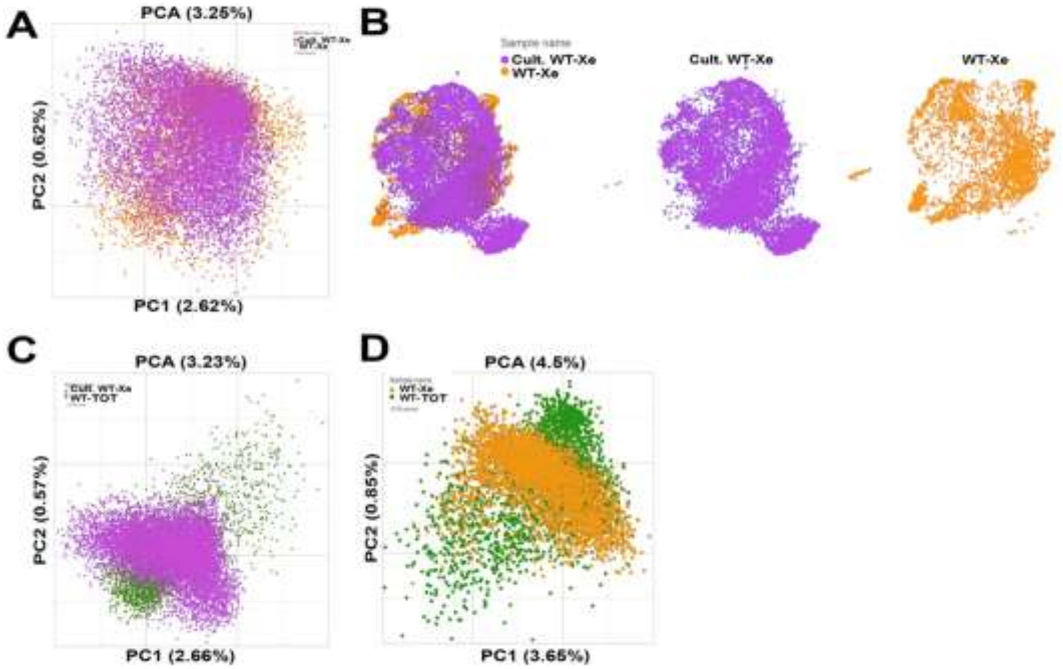

Supplementary Figure 15: Principal component (PCA) and integration analysis of WT SIX2+CITED1+, WT-Xe fresh, WT-Xe cultured and WT-TOT cells.

A. Uniform Manifold Approximation and Projection (UMAP) of droplet-based scRNA-seq profiles from the integration of WT-Xe fresh cells (orange dots) and WT-Xe cultured cells (purple dots,

generated with SIX2+CITED1+ cells cultured for 6 passages) confirming similarity (high overlap) of data from the two populations. **B.** PCA displaying WT-Xe cultured cells (purple dots, generated with SIX2+CITED1+ cells previously cultured for 6 passages) and WT-TOT (green dots) cells along PC1 and PC2, which describe 2.66% and 0.57% of the variability, respectively, within the data set. **C.** UMAP of 11370 droplet-based scRNA-seq profiles from the integration of WT-Xe cultured cells (purple dots, generated with SIX2+CITED1+ cells previously cultured for 6 passages), and WT-TOT cells (green dots) showing separation of the two samples, with some cells of WT-TOT and WT-Xe cultured cells clustering together. **D.** PC analysis displaying WT-Xe cells (orange dots) and WT-TOT (green dots) cells along PC1 and PC2, which describe 3.65% and 0.85% of the variability, respectively, within the data set.

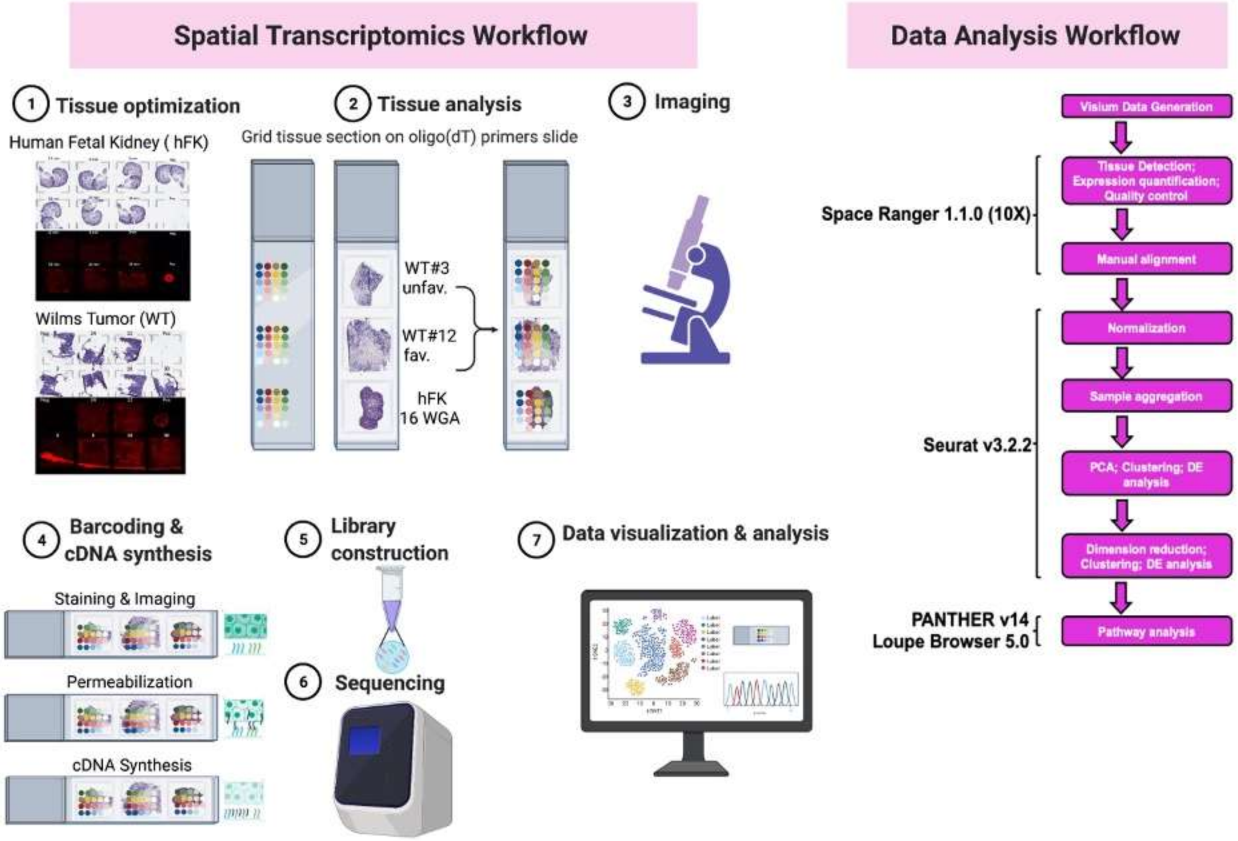

Supplementary Figure 16: Schematic representation of Spatial Transcriptomics (ST) protocol and analysis.

Schematic representation of the workflow and analysis for the ST Visium 10x Genomics. Created using BioRender.com.

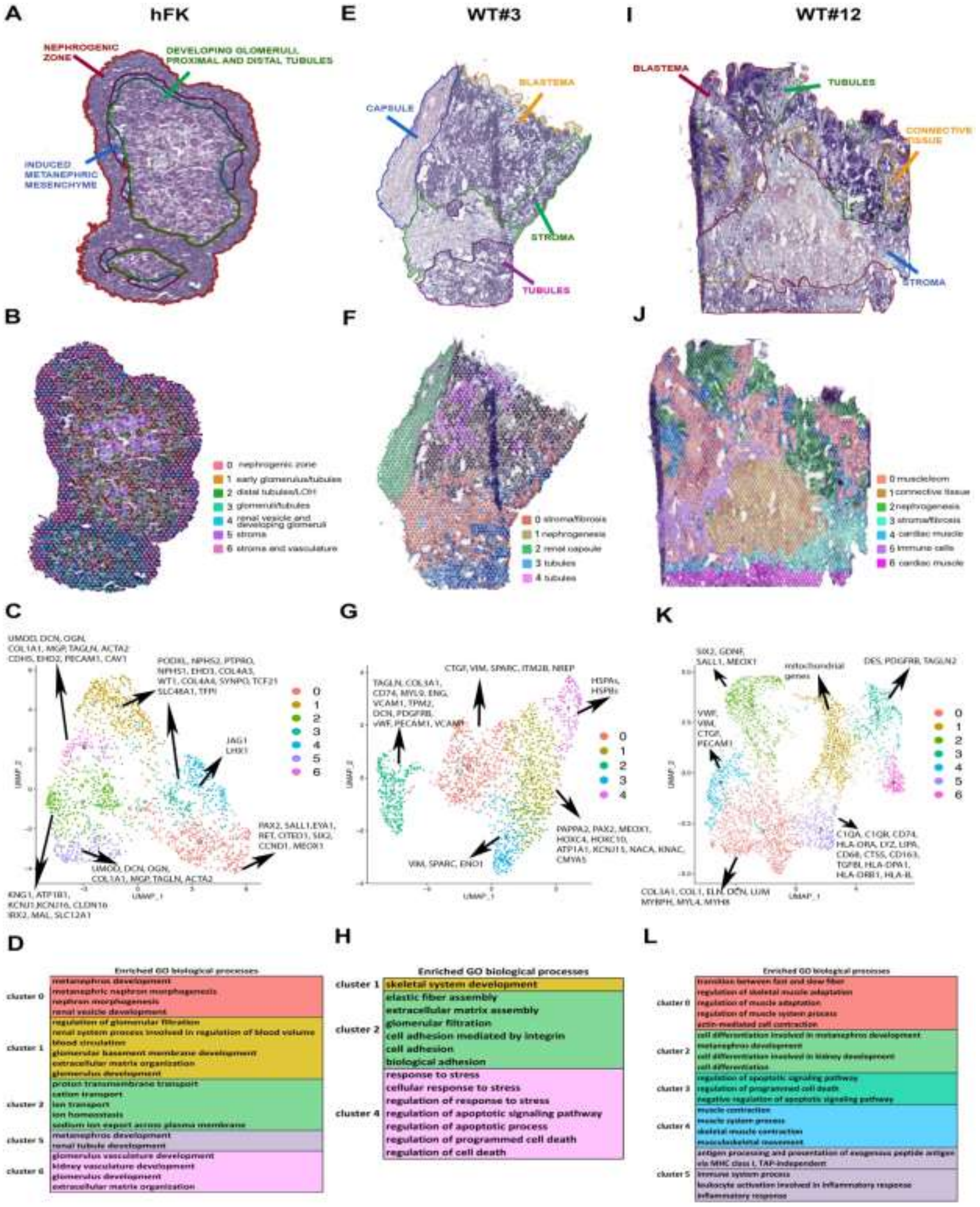

Supplementary Figure 17: Spatial Transcriptomic (ST) analysis of hFK, WT#3 and #4.

A-D. ST analysis of hFK (16.6 WGA). **A.** Histological identification of the hFK nephrogenic niche, red; developing renal structures, green; induced mesenchyme, blue. **B**. ST analysis

identified 7 clusters by unsupervised clustering. Gene expression signature of each cluster in the figure. (See Supplementary Discussion #2) **C.** Uniform Manifold Approximation and Projection (UMAP) of 1774 spot-based ST from hFK; color-coded clusters generated by unsupervised assignment. Specific cluster genes are reported. **D**. Gene Ontology (GO) analysis for selected clusters revealed a developmentally normal nephrogenic signature in different clusters. P<0.05. Upregulated DE genes were used for each comparison**. E-H**. ST analysis of WT#3, unfavorable stage I. **E.** Histology (capsule, blastema, stroma, tubules) of the unfavorable WT. **F**. ST analysis identified 5 clusters by unsupervised clustering, with distinct transcript-level signatures. (See Supplementary Discussion #2)**. G.** UMAP of 1773 spot-based ST from unfavorable WT, color-coded clusters generated by unsupervised assignment. Specific cluster genes are reported. **H**. GO analysis for selected clusters showed an aberrant differentiation pattern with co-expressed renal and muscle development genes. P<0.05. Upregulated DE genes were used for each comparison. **I-L**. ST analysis of WT#12; favorable stage III; **I.** Histology (blastema, tubules, connective tissue, stroma) of the favorable WT. **J**. ST analysis identified 7 clusters by unsupervised clustering; the specific transcriptomic signature of each cluster was reported in the figure (see Supplementary Discussion # 1)**. K**. UMAP of 3735 spot-based ST from unfavorable WT, color-coded clusters generated by unsupervised assignment. Specific cluster genes are reported. **L**. GO analysis for selected clusters confirmed deviant nephrogenesis characterized by renal maturation, apoptosis, and muscle differentiation genes. P<0.05. Upregulated DE genes were used for each comparison.

Cluster	GO biological processes (enriched, based on upreg. genes)
Cluster 0	actin-myosin filament sliding (GO:0033275)
	musculoskeletal movement (GO:0050881)
	extracellular matrix organization (GO:0030198)
	striated muscle cell differentiation (GO:0051146)
	muscle structure development (GO:0061061)
	circulatory system development (GO:0072359)
	heart development (GO:0007507)
	vasculature development (GO:0001944)
	tissue development (GO:0009888)
Cluster 1	muscle filament sliding (GO:0030049)
	actin-myosin filament sliding (GO:0033275)
	rRNA metabolic process (GO:0016072)
	cellular nitrogen compound catabolic process (GO:0044270)
	organic cyclic compound catabolic process (GO:1901361)
	extracellular matrix organization (GO:0030198)
	macromolecule catabolic process (GO:0009057)
	macromolecule biosynthetic process (GO:0009059)
Cluster 2	mesenchymal to epithelial transition (GO:0060231)
	metanephric nephron morphogenesis (GO:0072273)
	metanephros morphogenesis (GO:0003338)
Cluster 3	glomerulus development (GO:0032835)
	metanephros development (GO:0001656)
	nephron epithelium development (GO:0072009)
	mesonephros development (GO:0001823)
	angiogenesis (GO:0001525)
Cluster 4	metanephric nephron morphogenesis (GO:0072273)
	branching involved in ureteric bud morphogenesis (GO:0001658)
	ureteric bud development (GO:0001657)
	mesonephric tubule development (GO:0072164)
	kidney epithelium development (GO:0072073)
	skeletal system development (GO:0001501)
	heart development (GO:0007507)
Cluster 5	glomerular epithelium development (GO:0072010)
	negative regulation of stem cell differentiation (GO:2000737)
	positive regulation of G2/M transition of mitotic cell cycle (GO:0010971)
	positive regulation of cell cycle G2/M phase transition (GO:1902751)
	regulation of morphogenesis of a branching structure (GO:0060688)
	tight junction organization (GO:0120193)
	vasculogenesis (GO:0001570)
	mitochondrial ATP synthesis coupled electron transport (GO:0042775)
	integrin-mediated signaling pathway (GO:0007229)
	Notch signaling pathway (GO:0007219)
	renal system process (GO:0003014)
Cluster 6	cell cycle phase transition (GO:0044770)
	mitotic M phase (GO:0000087)
	M phase (GO:0000279)
	cell cycle process (GO:0022402)
	mitotic cell cycle (GO:0000278)
Cluster 7	interferon-gamma-mediated signaling pathway (GO:0060333)
	regulation of humoral immune response (GO:0002920)
	regulation of complement activation (GO:0030449)
	B cell mediated immunity (GO:0019724)
	platelet degranulation (GO:0002576)
	regulation of T cell proliferation (GO:0042129)
	lymphocyte mediated immunity (GO:0002449)
	positive regulation of T cell activation (GO:0050870)
	adaptive immune response (GO:0002250)
	angiogenesis (GO:0001525)
	regulation of T cell activation (GO:0050863)
Cluster 8	muscle filament sliding (GO:0030049)
	skeletal muscle contraction (GO:0003009)
	striated muscle contraction (GO:0006941)
	muscle system process (GO:0003012)
	heart process (GO:0003015)
	muscle organ development (GO:0007517)
	extracellular matrix organization (GO:0030198)
	ossification (GO:0001503)
	Wnt signaling pathway (GO:0016055)
	cell-cell signaling by wnt (GO:0198738)
	circulatory system development (GO:0072359)

Cluster	GO biological processes (Not enriched, based on downreg. genes)
Cluster 0	glomerulus development (GO:0032835)
	metanephros development (GO:0001656)
	ureteric bud development (GO:0001657)
	mesonephric epithelium development (GO:0072163)
	nephron development (GO:0072006)
	nephron tubule development (GO:0072080)
	kidney development (GO:0001822)
	renal system development (GO:0072001)
Cluster 1	glomerulus development (GO:0032835)
	mesonephros development (GO:0001823)
	ureteric bud morphogenesis (GO:0060675)
	kidney epithelium development (GO:0072073)
	nephron development (GO:0072006)
	nephron tubule development (GO:0072080)
	kidney development (GO:0001822)
	renal system development (GO:0072001)
	epithelial tube morphogenesis (GO:0060562)
Cluster 2	muscle filament sliding (GO:0030049)
	actin-myosin filament sliding (GO:0033275)
	mitochondrial ATP synthesis coupled electron transport (GO:0042775)
	ATP synthesis coupled electron transport (GO:0042773)
	ATP metabolic process (GO:0046034)
	muscle cell development (GO:0055001)
	cell cycle phase (GO:0022403)
	mitotic cell cycle phase (GO:0098763)
Cluster 3	muscle filament sliding (GO:0030049)
	skeletal muscle contraction (GO:0003009)
	muscle cell development (GO:0055001)
	skeletal muscle organ development (GO:0060538)
Cluster 4	mitochondrial ATP synthesis coupled electron transport (GO:0042775)
	ATP synthesis coupled electron transport (GO:0042773)
	oxidative phosphorylation (GO:0006119)
	ATP metabolic process (GO:0046034)
	cell cycle phase (GO:0022403)
	mitotic cell cycle phase (GO:0098763)
	regulation of cell cycle phase transition (GO:1901987)
	blood vessel morphogenesis (GO:0048514)
	regulation of mitotic cell cycle (GO:0007346)
	heart development (GO:0007507)
	circulatory system development (GO:0072359)
	mitotic cell cycle (GO:0000278)
Cluster 5	muscle filament sliding (GO:0030049)
	actin-myosin filament sliding (GO:0033275)
	striated muscle contraction (GO:0006941)
	tissue development (GO:0009888)
Cluster 6	nephron development (GO:0072006)
	renal system development (GO:0072001)
	glomerulus development (GO:0032835)
	ureteric bud morphogenesis (GO:0060675)
	mesonephros development (GO:0001823)
	ATP synthesis coupled electron transport (GO:0042773)
	ATP metabolic process (GO:0046034)
Cluster 7	actin-myosin filament sliding (GO:0033275)
	muscle filament sliding (GO:0030049)
	mitochondrial ATP synthesis coupled proton transport (GO:0042776)
	ATP synthesis coupled proton transport (GO:0015986)
	NADH dehydrogenase complex assembly (GO:0010257)
	S phase (GO:0051320)
	mitotic S phase (GO:0000084)
	interleukin-12-mediated signaling pathway (GO:0035722)
	G1 phase (GO:0051318)
Cluster 8	glomerulus development (GO:0032835)
	nephron development (GO:0072006)
	mesonephros development (GO:0001823)
	kidney development (GO:0001822)
	Notch signaling pathway (GO:0007219)

Supplementary Figure 18: Gene Ontology (GO) analysis of the integrated data from Spatial Transcriptomics (ST).

GO analysis for each cluster was identified in the integrated data of the ST collected from hFK and WT samples. Left: GO terms are upregulated in each cluster. Right: downregulated GO terms for each cluster. P<0.05.

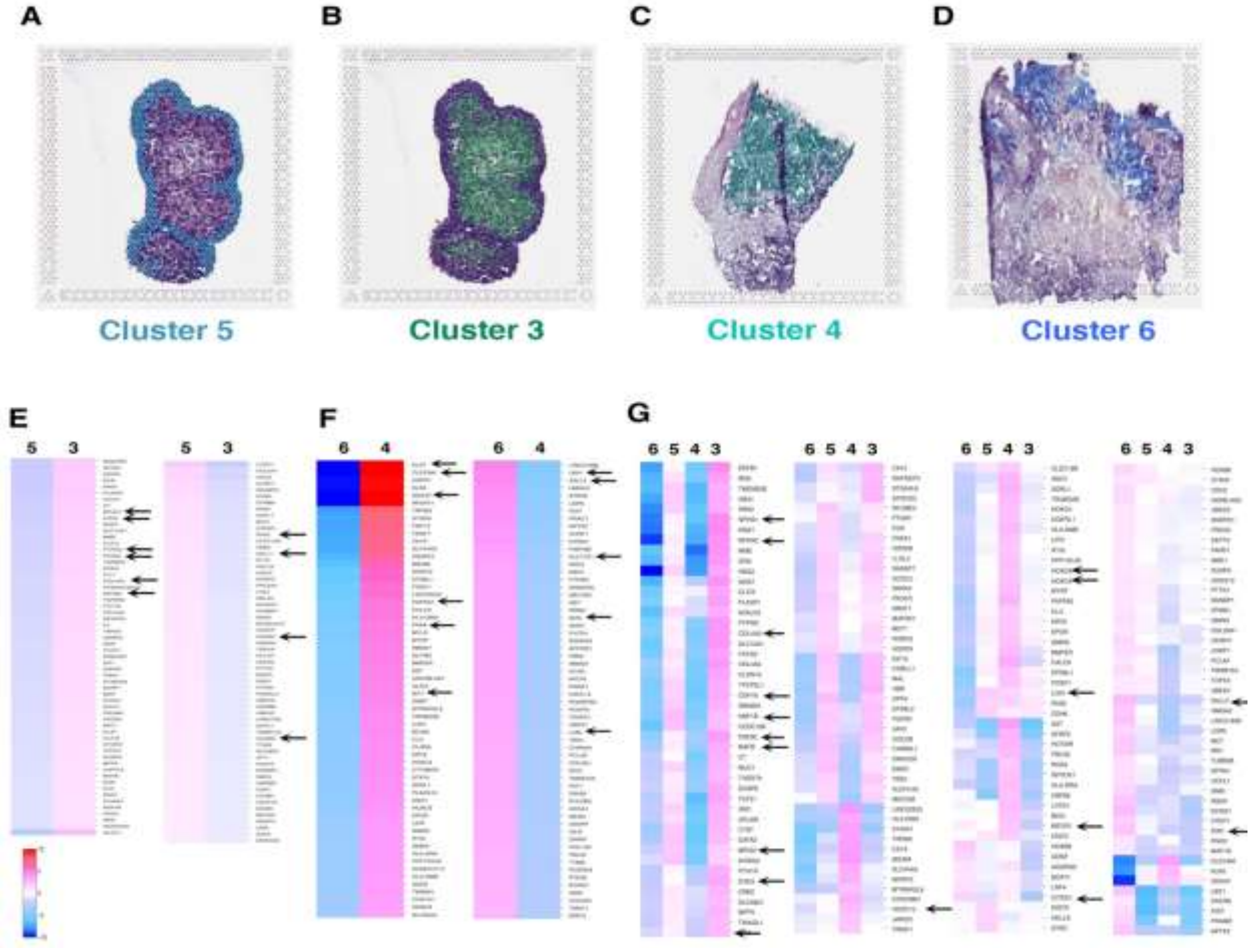

Supplementary Figure 19: Clusters 3, 4, 5, and 6 analysis of Spatial Transcriptomics (ST) integration data.

A-D. Spatial visualization of cluster 5 (hFK, **A**), cluster 3 (hFK, **B**), cluster 4 (WT#3, **C**), and cluster 6 (WT#12, **D**) obtained by unsupervised clustering of ST data performed on integrated data from hFK (16.6 WGA), WT#12 (favorable stage III) and WT#3 (unfavorable stage I)**.** Cluster 3 represents the nephrogenic differentiation steps, cluster 5 represents the cap mesenchyme, while clusters 4 and 6 come from blastema in WT#3 and #12, respectively. Cluster localizations are color-coded with the label below each image. **E**. Heatmap of the DE genes between clusters 3 and 5 from the integration analysis. Cluster 3 is more skewed toward

nephrogenic differentiation, while cluster 5 reflects a more uncommitted NP state. **F**. Heatmap of the DE genes between clusters 4 and 6 from the integration analysis showing higher early kidney development signature in cluster 4. **G**. Heatmap of DE genes among clusters 3, 4, 5, and 6 from the integration analysis showing that cluster 6 displays a high expression of myogenic genes (*MYOG*, *MYL1*, *MYH3*, *MYL4)* compared to cluster 5, and of *CITED1*, *SIX2*, *SALL3* compared to cluster 3, suggesting both a muscle differentiation-prone and uncommitted state compared to cluster 3. For **E-G**: genes of interest are marked by a black arrow.

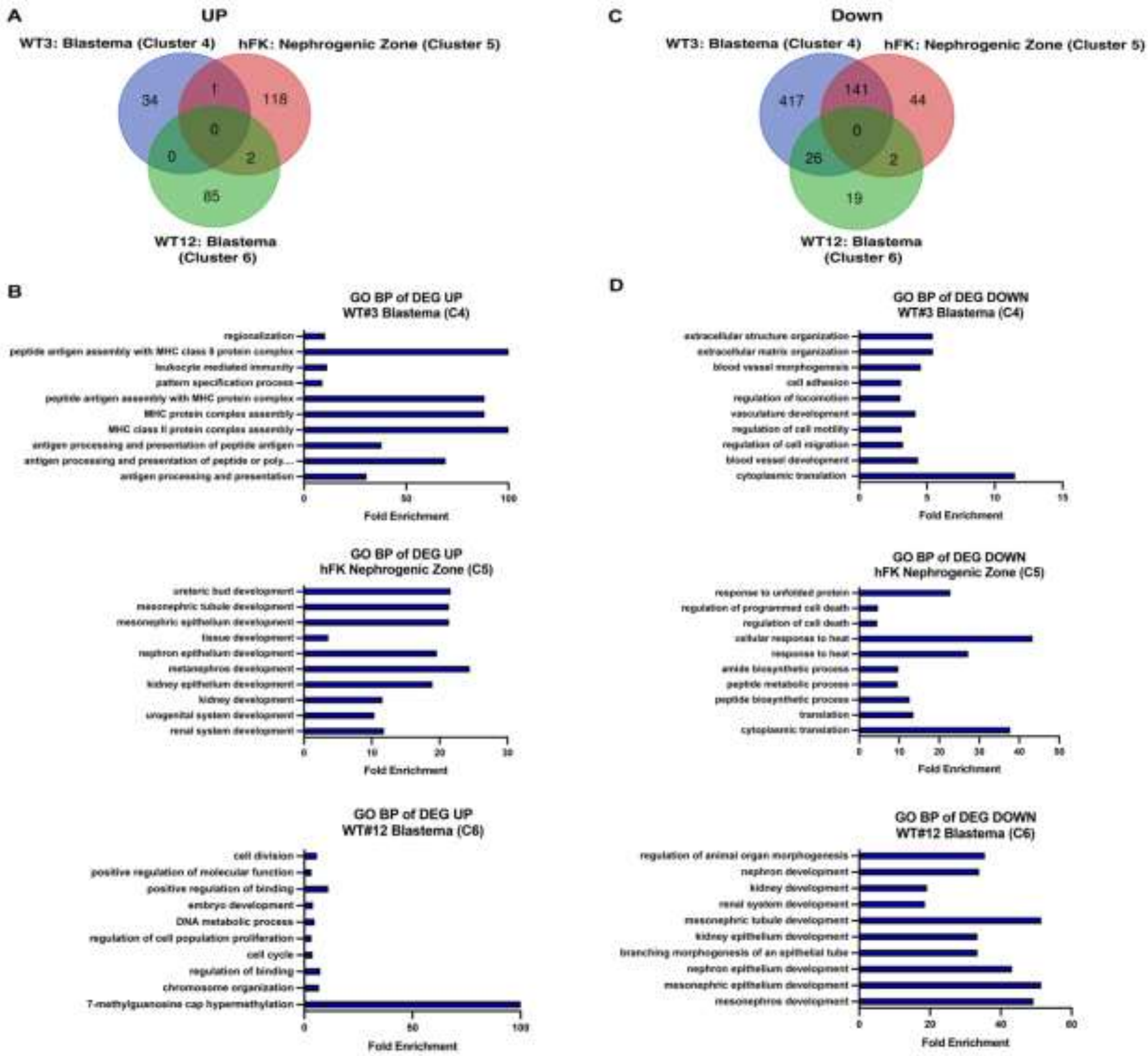

Supplementary Figure 20: Venn diagram of differentially expressed genes (DEG) analysis of Spatial Transcriptomics (ST) integration data.

A. Venn diagram of upregulated DEG in hFK (red) nephrogenic zone cluster 5 and WT#3 (blue) and 12 (green) blastema clusters 4 and 6, respectively. Only DEG with average log fold change

>0.5 or <-0.5 and adjusted p-value <0.05 were included. P<0.05. **B.** Analysis of the top-upregulated Gene Ontology (GO) biological process of DEG only found in hFK nephrogenic zone (cluster 5, 118 genes), WT#3 blastema (cluster 4, 34 genes), and WT#12 blastema (cluster 6, 85 genes). P<0.05. **C.** Venn diagram of downregulated DEG in hFK (red) nephrogenic zone cluster 5 and WT#3 (blue) and 12 (green) blastema clusters 4 and 6, respectively. Only DEG with average log fold change >0.5 or <-0.5 and adjusted p-value <0.05 were included. P<0.05. **D.** Analysis of the top-downregulated GO biological process of DEG only found in hFK nephrogenic zone (cluster 5, 44 genes), WT#3 blastema (cluster 4, 417 genes), and WT#12 blastema (cluster 6, 12 genes), P<0.05.

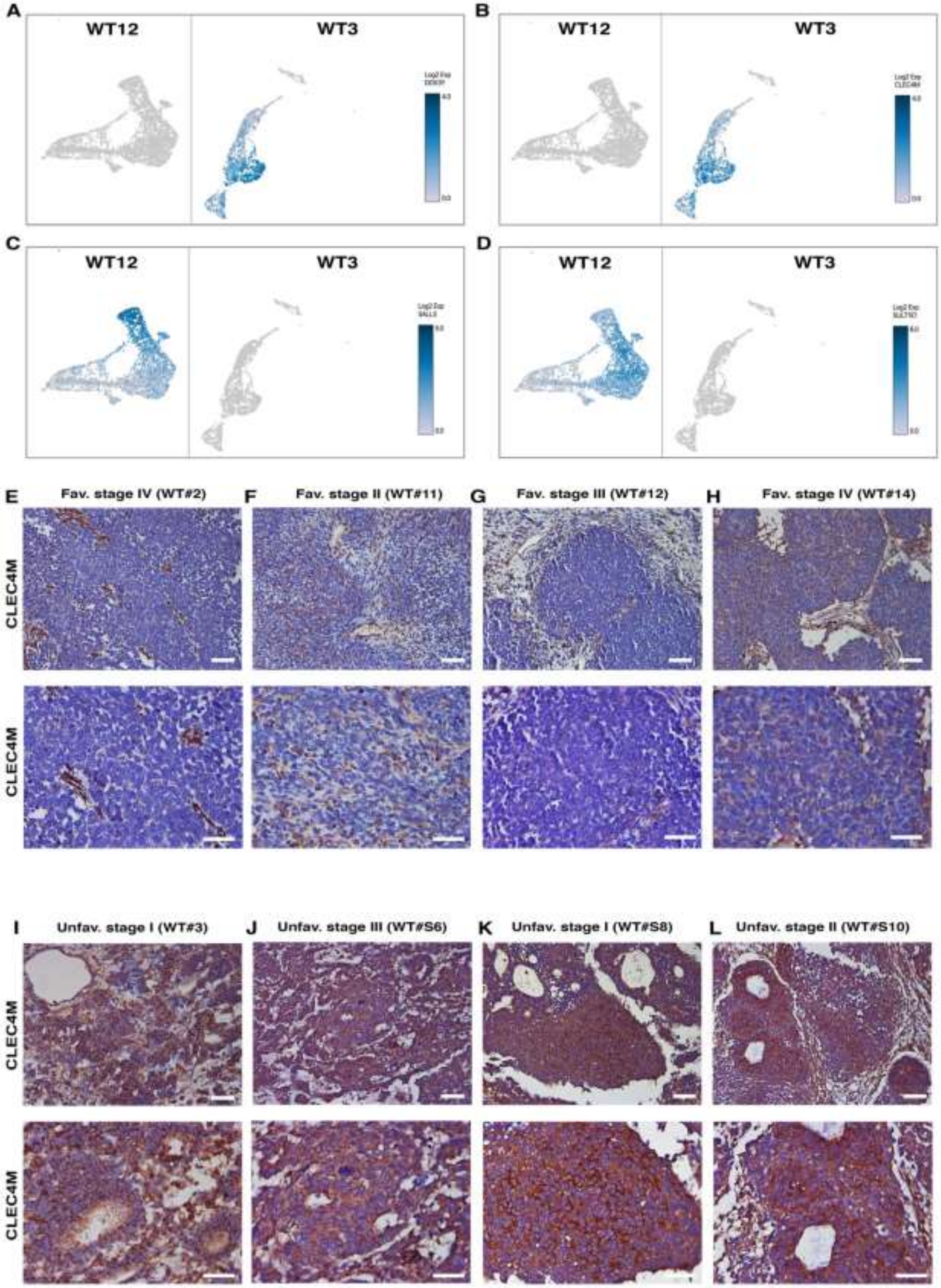
A
WT12
WT3
B
WT12
WT3
C
WT12
WT3
D
WT12
WT3
E
Fav. stage IV (WT#2)
F
Fav. stage II (WT#11)
G
Fav. stage III (WT#12)
H
Fav. stage IV (WT#14)
CLEC4M
CLEC4M
I
Unfav. stage I (WT#3)
J
Unfav. stage III (WT#S6)
K
Unfav. stage I (WT#S8)
L
Unfav. stage II (WT#S10)
CLEC4M
CLEC4M

Supplementary Figure 21: Specific gene expression patterns in unfavorable and favorable WTs identified by Spatial Transcriptomics (ST).

A-D. Uniform Manifold Approximation and Projection (UMAP) embedding spot colored for expression of *DDX3Y* (**A**), *CLEC4M* (**B**), *SALL3* (**C**), *SULT1E1* (**D**) from the integrated data analysis showing expression of *DDX3Y* and *CLEC4M* in unfavorable WT and *SALL3* and *SULT1E1* predominantly in favorable WT. **E-L.** Representative immunohistochemical staining for CLEC4M (dark brown) in WT favorable (stage IV, WT#2; stage II, WT#11; stage III, WT#12, stage IV, WT#14), and in WT unfavorable (stage I, WT#3, stage III, WT#S6; stage I, WT#S8; stage II, WT#S10) showing higher expression of CLEC4M within the blastema of unfavorable WT. In the favorable WT, CLEC4M was slightly expressed only in the vasculature and stroma. Nuclei stained with hematoxylin (blue). Scale bar=50 µm.

A

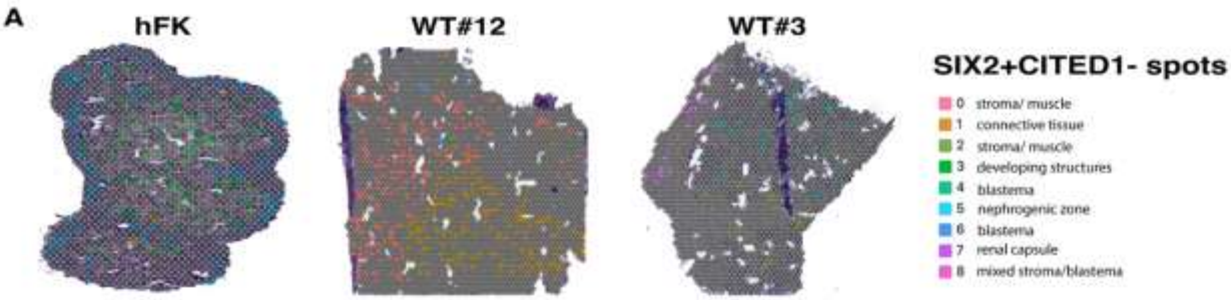

B

Cluster	0	1	2	3	4	5	6	7	8	SIX2+CITED1-	Total Spots	%
hFK	0	0	11	147	1	108	0	0	0	267	1774	15.05
WT12	246	151	20	7	1	0	23	0	2	450	3735	12.05
WT3	0	0	15	0	22	1	0	21	0	59	1766	3.34

C

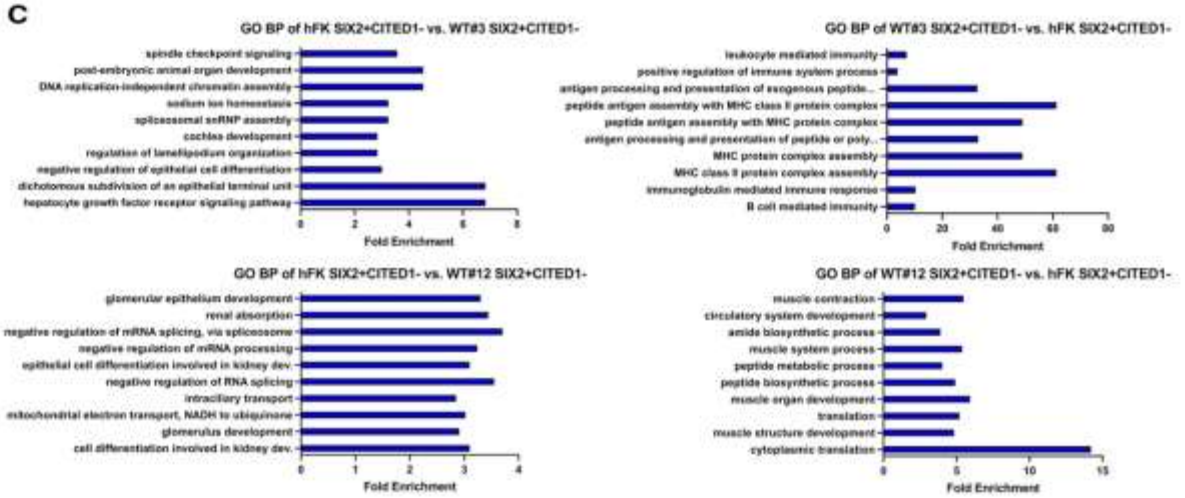

D

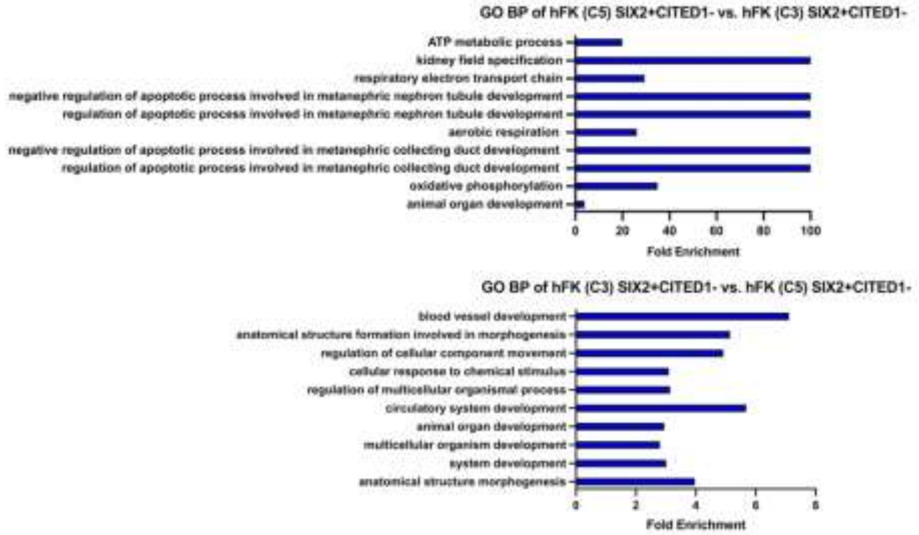

Supplementary Figure 22: Spatial Transcriptomics (ST) analysis of *SIX2*+ and *CITED1*- spots of integrated data from hFK and WT samples

A. Clusters color-coded by unsupervised assignment. ST visualization of *SIX2+CITED1-* spots on hFK, WT#12, and WT#3 obtained by unsupervised clustering of ST performed on integrated data. In the hFK, spots identified as SIX2+CITED1- are far from the nephrogenic zone. In WT#3 and WT#12, spots were identified to spread throughout the tumor, not specifically in the blastema. **B.** Calculation of ST spots *SIX2+CITED1-* in hFK, WT#3, and WT#12. The percentage of *SIX2+CITED1-* spots compared to the total number of spots per cluster (right side column) is shown. **C.** Analysis of the top Gene Ontology (GO) biological process of WT#3 compared to hFK. P<0.05; upregulated DE genes were used for each comparison. **D.** Analysis of the top GO biological process of hFK Cluster 5 (nephrogenic zone) compared to hFK Cluster 3 (developing structures). P<0.05; upregulated DE genes were used for each comparison.

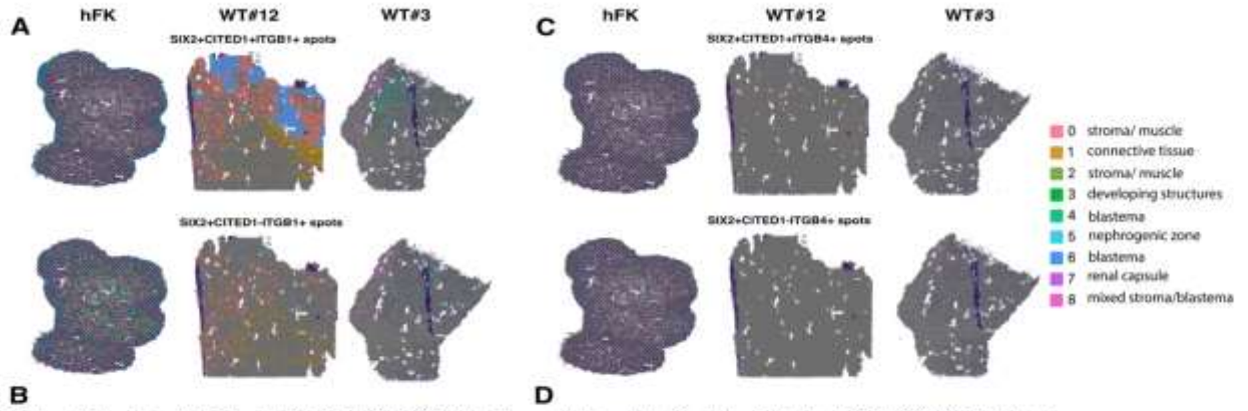

B

Cluster	0	1	2	3	4	5	6	7	8	SIX2+CITED1+ITGB1+	SIX2+CITED1+	%
hFK	0	0	4	28	0	119	0	0	0	151	152	99.3
WT#12	738	208	32	12	23	2	564	0	2	1581	1617	97.8
WT#3	0	0	13	0	78	0	0	34	0	125	133	94.0

Cluster	0	1	2	3	4	5	6	7	8	SIX2+CITED1-ITGB1+	SIX2+CITED1-	%
hFK	0	0	9	145	0	105	0	0	0	259	267	97.0
WT#12	241	116	16	6	1	0	23	0	2	405	450	90.0
WT#3	0	0	9	0	20	1	0	20	0	50	59	84.7

D

Cluster	0	1	2	3	4	5	6	7	8	SIX2+CITED1+ITGB4+	SIX2+CITED1+	%
hFK	0	0	0	1	0	23	0	0	0	24	152	15.8
WT#12	24	8	0	0	1	0	15	0	0	48	1617	3.0
WT#3	0	0	0	0	2	0	0	2	0	4	133	3.0

Cluster	0	1	2	3	4	5	6	7	8	SIX2+CITED1-ITGB4+	SIX2+CITED1-	%
hFK	0	0	0	17	0	18	0	0	0	35	267	13.1
WT#12	7	2	0	0	0	0	1	0	0	10	450	2.2
WT#3	0	0	0	0	0	1	0	0	0	1	59	1.7

Supplementary Figure 23: Spatial Transcriptomics (ST) of *SIX2, CITED1, ITGβ1*, and *ITGβ4* spots of integrated data from hFK and WT samples

A. Clusters color-coded by unsupervised assignment. ST visualization of *SIX2+CITED1+ITGβ1+* and *SIX2+CITED1+ITGβ1-* spots on hFK, WT#12, and WT#3 obtained by unsupervised clustering of ST performed on integrated data. In the hFK *SIX2+CITED1+ITGβ1+*, spots are in the nephrogenic zone (identified by cap mesenchyme), while hFK *SIX2+CITED1-ITGβ1+* are far from the nephrogenic zone (identified around developing structures such as the renal vesicles). **B.** Calculating ST spots expressing *SIX2+CITED1+ITGβ1+* and *SIX2+CITED1-ITGβ1+* in hFK, and WT#3 and WT#12. The percentage of *SIX2+CITED1+ITGβ1+* spots compared to the total number of spots per cluster (right side column) is shown. **C.** Clusters color-coded by unsupervised assignment. ST

visualization of *SIX2+CITED1+ITGβ4+* and *SIX2+CITED1+ITGβ4-* spots on hFK, WT#12, and WT#3 obtained by unsupervised clustering of ST performed on integrated data. **D.** Calculating ST spots expressing *SIX2+CITED1+ITGβ4+* and *SIX2+CITED1-ITGβ4+* in hFK, and WT#3 and WT#12. The percentage of *SIX2+CITED1+ITGβ4+* and *SIX2+CITED1-ITGβ4+* spots compared to the total number of spots per cluster (right side column) are shown.

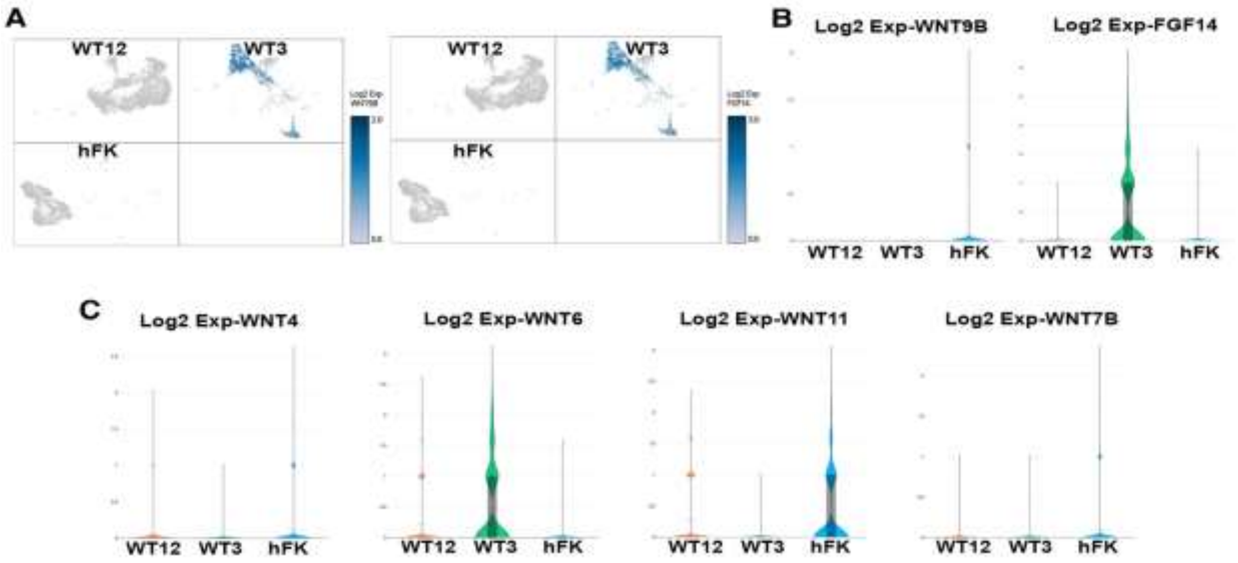

Supplementary Figure 24: Spatial Transcriptomics (ST) analysis of integrated data reveals different patterns of WNT and FGF expression in hFK and WT samples.

A. Uniform Manifold Approximation and Projection (UMAP) embedding spot-colored for expression of *WNT9b* and *FGF14* from the integrated data analysis. *WNT9b* expression was detected only in hFK and *FGF14* only in unfavorable WT. **B**. Violin plot for *WNT9b* and *FGF14*. Higher expression is detected in hFK and unfavorable WT, respectively. **C.** Violin plots confirming higher expression of *WNT4*, *WNT11* and *WNT7b* in hFK compared to WT samples; an exception to this pattern is *WNT6*, predominantly expressed in unfavorable WT.

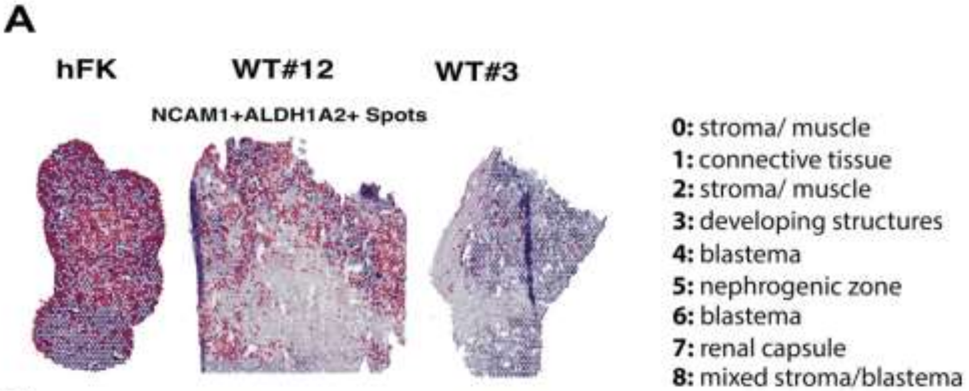

B

Cluster	0	1	2	3	4	5	6	7	8	NCAM1+ALDH1A2+	Total Spots	%
hFK	0	0	24	487	0	350	0	0	0	861	1774	48.53
WT12	677	96	18	9	14	0	240	0	1	1055	3735	28.25
WT3	0	0	11	0	28	0	0	11	0	50	1766	2.83

Supplementary Figure 25: Spatial Transcriptomics (ST) analysis of NCAM1 and ALDH1A2 spots of integrated data from hFK and WT samples

a. ST visualization of *NCAM1+ALDH1A2+* spots (red) on hFK, WT#12, and WT#3 obtained by unsupervised clustering of ST performed on integrated data. **B.** Calculation of ST spots with the expression of *NCAM1* and *ALDH1A2* in hFK, WT#12, and WT#3. In the hFK *NCAM1+ALDH1A2+* spots (48.53% of total spots) are expressed in cluster 3 (developing structures), followed by cluster 5 (nephrogenic zone). In WT#3, *NCAM1+ALDH1A2+* spots (2.83% of total spots) are largely identified in cluster 4 (blastema). In WT#12, *NCAM1+ALDH1A2+* spots (28.25% of total spots) are predominately present within cluster 0 (stroma muscle) and with a lesser degree in cluster 6 (blastema) and 1 (connective tissue).
